# A hundred species, mostly new—first assessment of ribbon worm diversity and distribution in Oman

**DOI:** 10.7717/peerj.19438

**Published:** 2025-05-28

**Authors:** Svetlana Maslakova, Irina Cherneva, Ethan Kahn, Audrey Wong, Gustav Paulay

**Affiliations:** 1 Oregon Institute of Marine Biology, Department of Biology, University of Oregon, Charleston, OR, United States of America; 2 Department of Invertebrate Zoology, Faculty of Biology, Moscow State University, Moscow, Russia; 3 Florida Museum of Natural History, University of Florida, Gainesville, FL, United States of America

**Keywords:** Taxonomy, Nemertea, Distribution, Species diversity, Temporary names, Arabia, Oman

## Abstract

**Background:**

Biodiversity is a key characteristic of any ecosystem but remains largely undescribed for most marine animals. Ribbon worms (phylum Nemertea), a diverse but poorly sampled phylum ubiquitous in the world’s oceans, are a case in point. Aside from their function as predators in marine communities, nemerteans are biomedically relevant because they produce diverse toxins, and some impact bivalve, decapod, and glass eel fisheries. Identification of nemerteans is challenging because many species look alike. The task is further complicated by many descriptions being based on preserved specimens, and therefore lacking characters of external appearance of live specimens. Characters of internal anatomy form the basis of traditional systematics but are more recently shown to be of little use in distinguishing between closely related species. This makes DNA data essential in species descriptions, and assessments of diversity and distribution.

**Methods:**

In a first modern survey of the phylum in Arabian waters, we collected nemerteans from a variety of habitats, focusing sampling on hard-bottom substrata, especially coral reefs. Specimens were triple-documented with photos, morphological vouchers, and DNA barcodes. Species delineation was based on morphology and Cytochrome Oxidase I sequences. Sequences and associated data are deposited in public databases, and vouchers at the Florida Museum of Natural History.

**Results:**

We documented 107 nemertean species in Oman, where none were previously known. This doubles the number of genetically characterized nemertean species for the entire Indo-West Pacific—a testament to how poorly sampled the phylum is in the most biodiverse marine region of the world. As many as 98% of the species were undescribed, and 93% are not documented outside Arabia. Half of the species were rare, and most—cryptic. Undescribed species were assigned unique alphanumeric temporary names for tracking in the literature and public databases. Estimates of source diversity suggest that future surveys might uncover an additional ∼200 species by including other locations and types of habitats, particularly soft bottoms, and the water column. Little overlap was observed between species found in the northern (Gulf of Oman) and southern (Sea of Arabia) regions, and many that occurred in both areas showed evidence of genetic differentiation corresponding to the major biogeographic break at R’as-al-Hadd.

**Conclusions:**

The high diversity, novelty, and distinctiveness of this fauna underscore the importance of sampling the most biodiverse and least studied tropical marine regions of the world. The large amount of cryptic and undescribed diversity highlights the critical role of DNA barcodes and rapid approaches to species descriptions.

## Introduction

Our knowledge of biodiversity is highly heterogeneous in time, space, and across taxa. There are over two billion occurrence records of birds in the Global Biodiversity Information Facility (GBIF, 2024), most of them identified to species, compared with ∼172K ribbon worms, a group that is likely of at least comparable species diversity, but ∼108K of the latter are not even identified to class. There are ∼160 million records from UK, compared with ∼0.5 million records from comparable sized Oman (GBIF, 2024). In this article we explore the biodiversity of an area and taxon of intermediate knowledge, document a macroinvertebrate phylum in an area not far from Europe, and demonstrate how little we know about life on Earth.

Ribbon worms (Nemertea) are a phylum of marine predatory worms with ∼1,350 accepted species (Kajihara et al., 2008; Norenburg et al., 2024). The group is characterized by an eversible proboscis used to capture and subdue prey, as well as to escape predation (Moor & Gibson, 1981; Maslakova & Norenburg, 2008a). These worms are found in most benthic marine habitats worldwide, and some have adapted to life in freshwater, terrestrial or pelagic marine environments (Gibson, 1972). Although not often numerous, large, or easy to find, because of their cryptic habits, nemerteans are ecologically important as predators (Mcdermott & Roe, 1985; Thiel & Kruse, 2001), biomedically relevant as producers of a wide array of toxins used for predation and defense (*e.g.*, Asakawa, Ito & Kajihara, 2013; Göransson et al., 2019; Verdes et al., 2022; Sonoda et al., 2023; Kem et al., 2024), and some are of importance to crustacean, bivalve, and glass eel fisheries as predators, parasites, or nuisance (Kuris & Wickham, 1987; Kuris et al., 1991; Bourque, Miron & Landry, 2001; Park et al., 2019; Dhanji-Rapkova et al., 2024).

Despite their importance, diversity, and ubiquitous presence in marine ecosystems, nemerteans remain among the lesser-known phyla of animals, with only a handful of active taxonomic experts in the world to identify and describe species. For example, out of 308 nemertean species described in the past decade, 151 are attributable to six authors, corresponding to only three research groups, led by Drs. Hiroshi Kajihara in Japan, Alexei V. Chernyshev in Russia, and Svetlana Maslakova in the USA (Norenburg et al., 2024). Species identification is hindered by the relative morphological simplicity of the group. External features, such as color and shape of the body, often do not survive preservation, while characters of internal anatomy, gleaned from serial histological sections (the basis of traditional systematics), do not differentiate between closely related species (*e.g.*, Maslakova & Norenburg, 2008b; Sundberg & Strand, 2010; Sundberg, Thuroczy Vodoti & Strand, 2010; Sundberg et al., 2016; Chen et al., 2010). Widespread morphological crypsis (*e.g.*, Leasi & Norenburg, 2014; Hao et al., 2015; Hiebert & Maslakova, 2015; Mendes et al., 2021; Liu, Kajihara & Sun, 2022; also see below), as well as the presence of polymorphic species (*e.g.*, Abato et al., 2024) make genetic data critical for species descriptions, delineation, and assessments of biodiversity and geographic distribution.

Global study of nemerteans parallels that of other poorly known taxa, with most of the research in the low-diversity faunas of Europe and North America, and the least in high-diversity tropics. Almost 29% (346 of 1,199 species) of recognized marine benthic nemerteans were described from the temperate North-East Atlantic (Europe), with another 60 species recorded in the North-West Atlantic (North America). In contrast, only 18% (217 species) are recorded from the largest and most diverse marine biogeographic region in the world, the tropical Indo-West Pacific (IWP), extending from the Red Sea and East Africa to Polynesia (Gibson, 1995, and S. Maslakova & G. Paulay, 2025, unpublished compilation). Furthermore, only 106 (9%) of the 1,230 putative nemertean species that are genetically characterized (approximated here from BOLD BINs) originated from the IWP prior to our recent sampling efforts in Arabia and Guam. In comparison, there are 167 nemertean BINs in the Southwestern Caribbean alone (Maslakova et al., 2022, and S. Maslakova, 2025, unpublished). Yet, in well-studied taxa like fishes and corals, the IWP is 2–10 times as diverse as the tropical West Atlantic, the second most diverse region (Paulay, 1997), and the tropics are 2–10 times more diverse than temperate and polar regions in well-studied groups like bivalves, brittle stars, and fishes (Stöhr, O’Hara & Thuy, 2012; Jablonski et al., 2013; Stuart-Smith et al., 2013).

While the IWP is biogeographically recognizable because of many taxa that span much of its extent, others show substantial variation and regional endemism. The Arabian region is notably divergent, with numerous endemics, but until recently received limited attention in most taxa (Di Battista et al., 2016; DiBattista et al., 2016). Oman, with a >1,600 km coastline, is among the least-known and most interesting areas. The boundary between the Gulf of Oman (northern Oman) and the Arabian Sea (southern Oman) at the northwestern tip of the Arabian Peninsula (R’as-al-Hadd) is “one of the sharpest biotic transitions known in marine biogeography” (Schils & Wilson, 2006), separating distinct marine ecoregions with substantially different oceanographic regimes (Wilson, 2000; Spalding et al., 2007; Belanger et al., 2012).

Our knowledge of nemerteans varies substantially across the vast IWP region. Very few historical (not accompanied by DNA barcoding) and no recent studies focus on the nemertean fauna of the waters surrounding the Arabian Peninsula, and those that exist are limited to the Red Sea (Ehrenberg, 1828; Joubin, 1893; Joubin, 1902; Gibson, 1974), the Gulf of Aden (Joubin, 1904), and the Persian/Arabian Gulf (Senz, 2000; Senz, 2001). The nemerteans of Oman (Gulf of Oman and Arabian Sea) are almost entirely unknown. This undoubtedly reflects a lack of sampling, rather than a lack of diversity. A study by McLachlan et al. (1998) on the ecology of sandy beaches of Oman reports significant numbers of “Nemertea spp.” among the fauna inhabiting intertidal soft sediments, but none have been identified to a lower taxonomic category or genetically characterized until our recent work there (Cherneva et al., 2023).

Three recent benthic marine invertebrate surveys across Oman (in January 2020, January, February, and November 2022) revealed a large, distinct, and almost entirely undescribed nemertean fauna. This study summarizes the results of these surveys, based on expert morphological identification of specimens by SM and IC, and analysis of Cytochrome Oxidase I (COI) sequence data.

## Materials & Methods

### Specimen collecting, preservation, and storage

**Figure 1:**
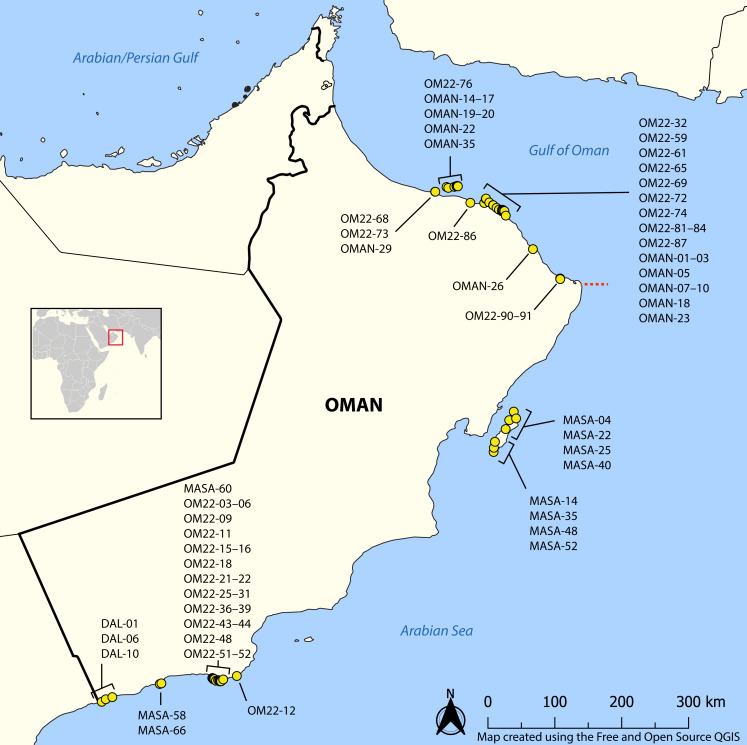
Map showing sampling locations of barcoded nemertean specimens. Red square corresponds to the enlarged area. Red dashed line shows the location of R’as-al-Hadd biogeographic boundary separating the fauna of the Gulf of Oman in the north from that of the Arabian Sea in the south. See BOLD dataset DS-NOMAN and Florida Museum of Natural History collection database for GPS coordinates, depth, and habitat data associated with each station. Map on inset has been modified from https://commons.wikimedia.org/wiki/File:BlankMap-World_gray.svg to crop and add labels under GNU Free Documentation license.

We collected 581 specimens of nemerteans from a variety of intertidal and shallow subtidal habitats in northern and southern Oman during the 2020 and 2022 Bioblitz expeditions (Fig. 1). Nemerteans were collected intertidally by hand from rocky shores and sand flats, and by snorkeling and SCUBA diving in the shallow subtidal (hard and soft bottoms). Worms were extracted from mass samples of consolidated substrata (coral rubble, algal holdfasts and mats, and vermetid-coralline and oyster reefs) by deoxygenation, referred to as “sweating” (Kirsteuer, 1967). Sampling was concentrated in the Gulf of Oman (Suwadi Island to Sur), north of the R’as-al-Hadd boundary, and in the Arabian Sea (Masirah Island and Dhofar), south of R’as-al-Hadd. Specimens were collected and exported to the United States with permission from the Environment Authority of Oman (permit 6210/10/151). Each specimen was assigned a unique field number (BOMAN-#####), representative individuals of each morphospecies were photographed live (including stylets for hoplonemerteans), tissue subsampled and preserved for DNA extraction in 95% ethanol, and the anterior end or bulk of the worm preserved as a morphological voucher. Specimens serving as morphological vouchers were relaxed in 7.5% MgCl_2_, then preserved in 10% buffered formalin (made up in filtered seawater). Detailed collecting information for all specimens can be found in the invertebrate zoology database of the Florida Museum of Natural History, University of Florida (UF Nemertea), where all vouchers and remaining tissue samples are deposited, as well as the BOLD dataset DS-NOMAN for sequenced specimens (see also Table S1).

### DNA barcoding

DNA barcoding was carried out at the Oregon Institute of Marine Biology (OIMB) for the majority of specimens, and at the Laboratory of Analytical Biology (National Museum of Natural History, Smithsonian Institution) for a smaller fraction (see Lasley et al. (2023) for SI lab protocols). At OIMB, DNA was extracted from 316 individuals using the DNEasy Blood and Tissue Kit (Qiagen). A 658–698 bp region of COI was amplified using a combination of universal metazoan COI primers LCO1498 and HCO2198 (Folmer et al., 1994), degenerate jgLCO1498 and jgHCO2198 (Geller et al., 2013), and a nemertean-specific reverse primer CO1-DR 5′GAGAAATAATACCAAAACCAGG-3′  (Cherneva et al., 2023) located just downstream of HCO2198. The combination of LCO1498 and CO1-DR primers amplifies a 698 bp region, which includes the 658 bp Folmer region. PCRs were carried out as described in Cherneva et al. (2023). PCR products that produced single bright bands of expected size were purified using SW Wizard Gel and PCR Purification System (Promega). Purified PCR products were sequenced in both directions using PCR primers at Sequetech Inc. (Mountain View, CA) or Eurofins Genomics (Louisville, KY). Sequences with <50% high-quality bases were discarded. High-quality sequences were trimmed to remove low quality end-regions and primers. Forward and reverse strands were assembled into contigs and proofread against each other in Geneious Prime 2022.1 (Biomatters). Bases with total Phred quality scores of <20 (>1% probability of erroneous base call) were converted to “N” in consensus sequences. Consensus sequences were translated using Invertebrate Mitochondrial genetic code, checked for stop codons, and BLASTed against the NCBI database. Sequences whose top BLAST matches were not from the phylum Nemertea were removed from the analysis as putative contaminants (possible prey sequences). Sequences whose top BLAST match was a nemertean, but from a different class (compared to morphological assessment), were treated as sample processing errors and removed from subsequent analyses, unless morphological identification was ambiguous (*e.g.*, tubulanid palaeonemertean *vs.* hubrechtellid pilidiophoran). To further check sequences for contamination or sample processing errors, sequences were aligned using the MAFFT plug-in with default parameters. Alignment was checked by eye. A Neighbor-Joining tree was constructed in Geneious Prime, using the Tamura-Nei genetic distance model. Clade composition was checked for taxonomic consistency, and suspect sequences were flagged and removed from subsequent analysis (File S1). Sequences and traces are deposited in BOLD (DS-NOMAN) and GenBank (see Table S1 for Field numbers, BOLD Process IDs, Florida Museum of Natural History and GenBank accession numbers, as well as collecting sites).

### Figure preparation

Specimen photographs have been minimally processed in Photoshop 2025 (Adobe) to crop, adjust brightness, contrast, or hue (where needed), or remove background using “select subject” function in Photoshop or “remove background” function in Preview (Apple). Figure plates were assembled in Illustrator 2025 (Adobe).

### Species delimitation and geographic distribution

A total of 299 COI sequences were aligned using MUSCLE (Edgar, 2004). The alignment (698 bp long) was checked by eye to ensure it did not include any gaps. Species delimitation was carried out using Assigning Species by Automatic Partitioning (ASAP, Puillandre, Brouillet & Achaz, 2021), using K-80 Kimura distances (ti/tv=2.0). Subsets or molecular operational taxonomic units (MOTUs) assigned by ASAP were checked against BOLD BINs, as an alternative (less conservative) criterion for species delineation. Because the ASAP delineation threshold depends on the dataset composition, while BIN delineation is more stable and universal, and because most of the BINs corresponded to reciprocally monophyletic and region-specific lineages, we used BINs to determine geographic distribution outside Oman by examining all “non-unique” BOLD BINs (those that contain records from outside this project in BOLD). The 7.5% distance threshold in ASAP analysis roughly corresponds to the minimum divergence (9%) seen between closely related, morphologically distinct, sympatric lineages of Nemertea in this dataset (*e.g.*, within the genus *Tetrastemma*, see below).

Temporary names consisting of a higher-level taxon ID (genus, family, or higher) and an alpha-numeric code (SMOM###) were assigned to MOTUs (putative species) that could not be matched to any described species (Tables S1–S3). The use of temporary names is becoming an accepted practice in nemertean systematics (*e.g.*, Kajihara, Ganaha & Kohtsuka, 2022; Ellison et al., 2024).

### Phylogenetic analyses

To identify prospective Oman-restricted clades, we placed a selection of representative sequences (one per BIN) from Oman in the context of all available nemertean BINs (from BOLD), plus additional sequences from GenBank, and our unpublished sequences from the Caribbean, Panama Bight, Red Sea, Guam, and Moorea. After eliminating likely contaminants (possible prey), we aligned sequences using MAFFT in Geneious Prime. The resulting alignment was 1,577 bp long and contained 1,303 sequences, each representing a unique nemertean lineage (Table S4). A Neighbor-Joining tree was constructed in Geneious Prime using the Tamura-Nei genetic distance model. Based on this preliminary analysis, we identified putatively Oman-restricted clades within the genera *Carinoma* and *Zygonemertes* and Arabian-restricted clades in several other genera, including *Gorgonorhynchus* and *Tetrastemma*.

We conducted separate phylogenetic analyses for the genera *Carinoma*, *Gorgonorhynchus, Tetrastemma*, and *Zygonemertes* to show putative recent radiations within Oman, or more broadly Arabia. These analyses included previously published representative congeneric sequences from the rest of the world (one sequence per BIN). GenBank Accession numbers of all previously published sequences are shown on relevant tree figures (see below).

For *Carinoma*, we included previously published congeneric sequences, plus two sequences identified as Nemertea sp. which appear to be *Carinoma* (KJ592725 from California, USA and MG421956 from Manitoba, Canada), and excluded one previously published sequence from Norway (KP697714) which is highly divergent and appears to be misidentified as *Carinoma*. *Tubulanus* sp. SMOM037 and *Cephalothrix* sp. SMOM036 from the Oman dataset were used as outgroups.

For *Gorgonorhynchus,* we used representative congeneric sequences from BOLD, including our unpublished sequences from the Red Sea (see BOLD dataset DS-GORW24), and a sequence from Bahamas (HQ848632) misidentified as Polystilifera sp. SA-2011 in GenBank (Andrade et al., 2012), which belongs to *Gorgonorhynchus*. *Polydendrorhynchus zhanjiangensis* (KC603702) and *Notospermus* sp. SMOM055 were used as outgroups.

Reference sequences for *Tetrastemma* included previously published and our unpublished sequences from the Red Sea, which appear to belong to the genus as redefined by Chernyshev et al. (2021), see BOLD dataset DS-TSTW24. Additionally, we included sequences of *Tetrastemma polyakovae* (ON021857), *Testrastemma strandae* (ON021856), and *Tetrastemma sundbergi* (ON021855) published by Sagorny et al. (2022) and *Tetrastemma cupido* (OK414013) published by Hookabe, Kohtsuka & Kajihara (2021), which are not currently in BOLD. Oerstediina gen. sp. SMOM025 and sp. SMOM028 from Oman served as outgroups.

For *Zygonemertes*, we included representative previously published congeneric sequences. Oerstediina gen. sp. SMOM025 and Oerstediina gen. sp. SMOM028 from Oman served as outgroups.

Sequences were aligned in Geneious Prime 2022.1.1, using MAFFT with default parameters, and the alignments were trimmed to the 658 bp between the Folmer primers. Bayesian Inference analyses were run using a Geneious Prime plugin for Mr. Bayes with GTR+I+G model, with a chain length of 500,000, burn-in of 10,000, and otherwise default parameters.

### Estimates of source diversity

Unsampled diversity was estimated based on the Chao1 index (Park et al., 2019), which predicts species richness based on the prevalence of singletons (species with an abundance of 1) and doubletons (species with an abundance of 2). Classical version of the Chao1 is calculated as S+F_1_^2^/2F_2_, where S is the number of observed species, F_1_ is the number of singletons, and F_2_ is the number of doubletons (Chao, 1984). We used a bias-corrected version of Chao1, calculated as S + F_1_(F_1_-1)/2(F_2_+1), which is now widely preferred (Deng, Umbach & Neufeld, 2024). Because these estimates rely on the homogeneity of sampling, we also calculated Chao1 index separately by type of habitat, coarsely divided into “hard bottom”, which included specimens obtained from consolidated substrata: coral rubble, vermetid-coralline assemblages, algal mats and holdfasts, and barnacle-oyster conglomerates, as well as those found under rocks on coarse sand, shell hash, or gravel, and “soft bottom”, which included truly infaunal worms found in sand or mud (dug up intertidally with a shovel or yabby pump, or scooped and sieved from sand underwater, while SCUBA diving).

## Results

### Habitats

**Figure 2:**
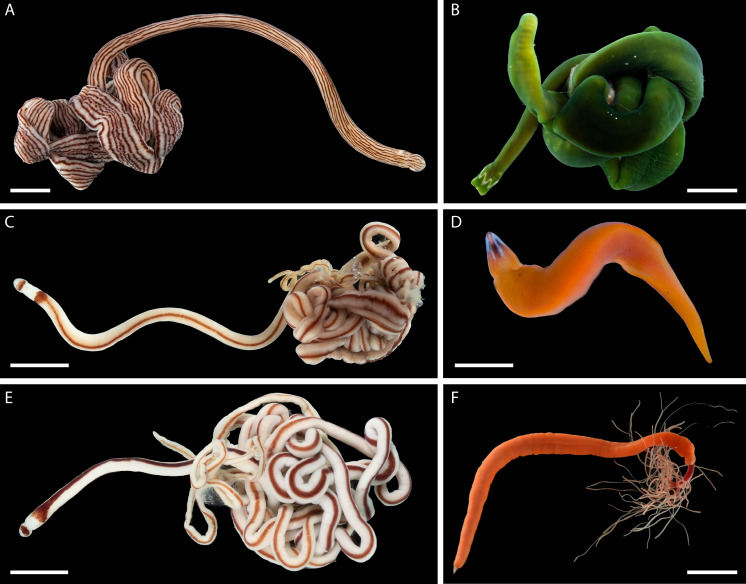
Large, common, and conspicuous nemerteans of Oman. (A) *Baseodiscus* cf. *insignis*, common in Dhofar, also found in Red Sea; individual BOMAN-09070. Sequences from the type locality (Zanzibar) are lacking to confirm identification. (B) *Notospermus* sp. SMOM055 (an undescribed Arabian look-alike of *Notospermus tricuspidatus*), common in Oman, also known from Red Sea; individual BOMAN-06419. (C) *Baseodiscus* sp. SMOM101, individual BOMAN-06733—an undescribed look-alike of *Baseodiscus hemprichi* (E); the former only known from Dhofar, where the two species co-occur, while the latter has a wide Indo-West Pacific distribution. (D) *Drepanophorus* sp. SMOM022, an undescribed reptant polystiliferan, common in Oman, also known from Red Sea, with look-alikes in other parts of IWP; individual BOMAN-02724. (E) *Baseodiscus hemprichii*, individual BOMAN-08268 from Dhofar. (F) *Gorgonorhynchus* sp. SMOM045, an uncatalogued individual from Masirah Island (lineage F on Fig. 8); note the everted dichotomously branched proboscis, characteristic of this genus. Scale bars: A—6 mm; B–C, E–F—1 cm; D—2 mm.

**Figure 3:**
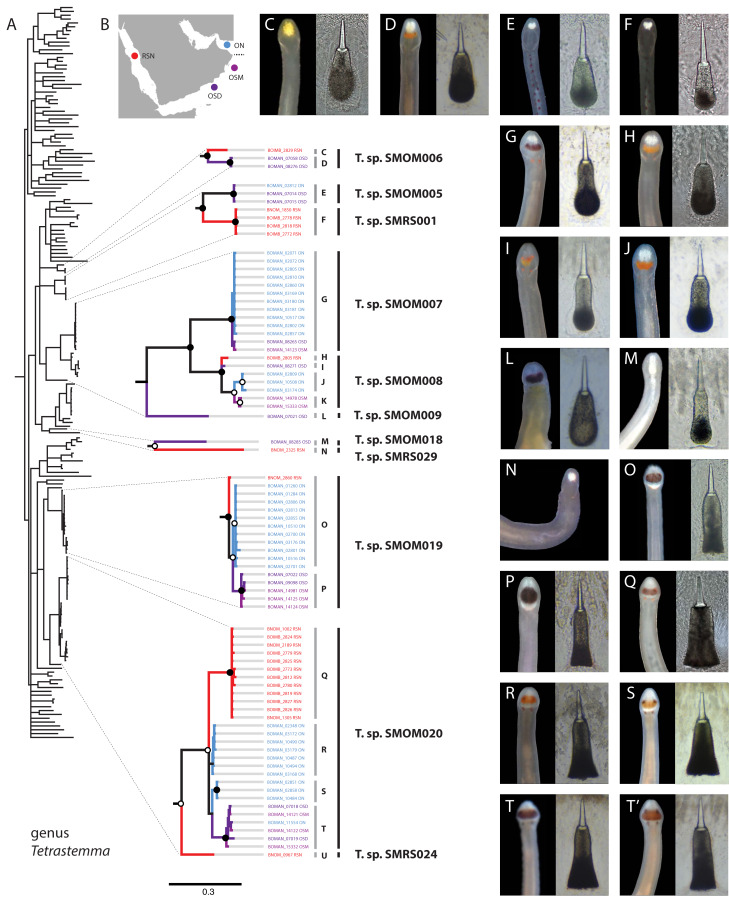
*Tetrastemma* spp. from Arabia in a global context. (A) Bayesian Inference tree based on the Cytochrome Oxidase I, including reference sequences. Well-supported Arabian clades are magnified. Fully supported clades are indicated by solid circles, those with posterior probability of 0.9 or higher—with open circles, clade support below 0.9 is not shown. Color of the terminals on the tree corresponds to sampling regions on the map (B). OSD—Dhofar, Southern Oman. OSM—Masirah Island, Southern Oman. ON—Northern Oman. RSN—Northern Red Sea. The dotted line on B shows the location of R’as al-Hadd, a major biogeographic boundary. Grey vertical lines correspond to BOLD BINs (identified by letters C-U), with correspondingly labeled panels of photographs of heads and central stylets of representative live specimens on the right (C-T’). Black vertical lines indicate MOTUs with corresponding temporary names. No photographs were available for BINs K and U, and no stylets for N. Panel T’ depicts a possible hybrid between the northern and southern lineages of *Tetrastemma* sp. SMOM020 (individual BOMAN-11554 from Northern Oman with a Southern Oman mtDNA (BIN T). Individuals of *T*. sp. SMOM020 from Northern Oman (BINs R and S) have lighter colored cephalic patch than those from Southern Oman (BIN T) and Red Sea (BIN Q), while the putative hybrid has an intermediate phenotype (T’).

**Figure 4:**
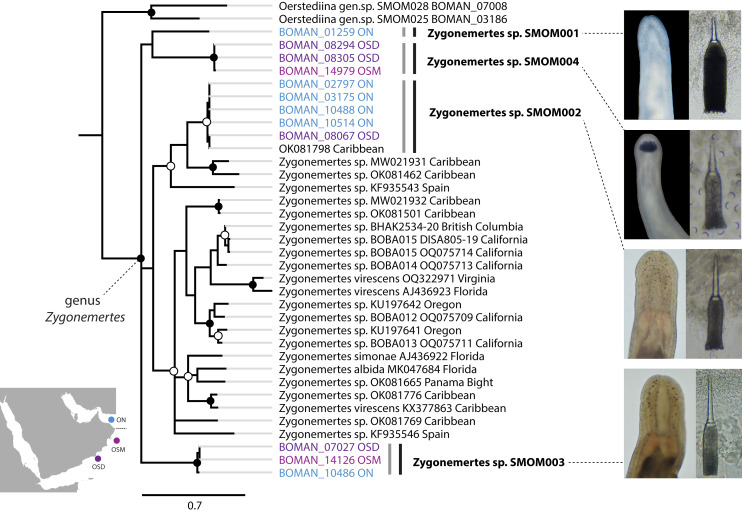
*Zygonemertes* spp. from Oman in a global context. Bayesian Inference tree based on the Cytochrome Oxidase I sequence data. Fully supported clades are indicated by solid circles, those with posterior probability of 0.9 or higher—with open circles, clade support below 0.9 is not shown. Sequences obtained in this study are color coded by sampling location as shown on the map (blue for Northern Oman, ON; purple for Dhofar in Southern Oman, OSD; magenta for Masirah Island in Southern Oman, OSM). Sequences from other parts of the world included for reference and outgroups are in black. Grey vertical lines correspond to BOLD BINs, and black vertical lines—to MOTUs, with corresponding temporary names in bold font. Representative photos of each Omani *Zygonemertes* MOTU are shown on the right. Note, that all Omani *Zygonemertes* spp. have truncated bases of central stylet, and sickle-like epidermal spicules (illustrated for *Zygonemertes* sp. SMOM004 in which stylet was photographed through the body wall), which characterize the genus *Zygonemertes*, as defined here. All four Omani *Zygonemertes* are white, and not all have ocelli posterior to the cerebral ganglia. *Zygonemertes* sp. SMOM001 has relatively long basis compared to length of central stylet, *Zygonemertes* sp. SMOM004 is distinguished by having a pigmented dark blue cephalic patch, while *Zygonemertes* sp. SMOM003 has fluted stylets.

**Figure 5:**
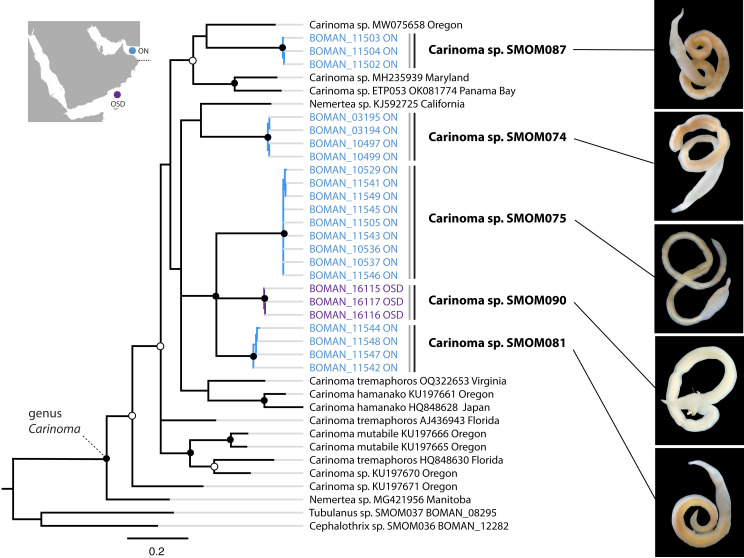
*Carinoma* spp. from Oman in a global context. Bayesian inference tree based on the Cytochrome Oxidase I sequence data. Fully supported clades are indicated by solid circles, those with posterior probability of 0.9 or higher—with open circles, clade support below 0.9 is not shown. Sequences obtained in this study are color coded by sampling location as shown on the map (blue for Northern Oman, ON; purple for Dhofar in Southern Oman, OSD). Sequences from other parts of the world included for reference and outgroups are in black. Grey vertical lines correspond to BOLD BINs, and black vertical lines—to MOTUs, with corresponding temporary names in bold font. Representative photos of each Omani *Carinoma* MOTU demonstrate morphological uniformity of *Carinoma* spp. Two of the reference sequences identified as “Nemertea sp.” appear to be unidentified *Carinoma* spp.

Nemerteans were found in most types of the habitats sampled, including subtidal coral rubble, intertidal and subtidal soft sediments, algal mats, intertidal oyster reefs, and shallow subtidal vermetid-coralline reefs, with the majority (∼60%) collected by “sweating” coral rubble. A few nemertean species in Oman are common, large (many centimeters long), conspicuous, and can be found intertidally and in the shallow subtidal under boulders or large pieces of coral rubble (Fig. 2); however, most are small (1–2 cm long or smaller), and represent part of the cryptobiota of habitats such as coral rubble, *e.g.*, *Tetrastemma* spp. (Fig. 3) and *Zygonemertes* spp. (Fig. 4) or soft sediment, *e.g.*, *Carinoma* spp. (Fig. 5). Almost 15% of the diversity (16 putative species) was represented by small, four-eyed species from the genus *Tetrastemma* (Table S2, Fig. 3).

### Diversity

Overall, we documented representatives of all three classes—the Palaeonemertea (17 species), Pilidiophora (44 species), and Hoplonemertea (45 species), all seven orders (Archinemertea, Carinomiformes, Tubulaniformes, Hubrechtiiformes, Heteronemertea, Monostilifera, and Polystilifera), and 31 genera (see Table S2 for a list of species and classification).

We successfully obtained COI barcodes from 299 individuals (151 from northern Oman and 148 from southern Oman). ASAP analysis identified a barcoding gap between 6–8% sequence divergence, and the best partition (at 7.5% K80 distance threshold) assigned specimens into 102 MOTUs. Of the 299 sequences, 296 have been assigned to 108 BINs in BOLD. Of the remaining three, only one represented a unique MOTU (*Tubulanus* sp. SMOM078) and would have certainly been placed into a unique BIN. See Table S1 for MOTU and BIN placements of all sequenced specimens.

Assignments into BINs were largely concordant with ASAP delineation into MOTUs, with five exceptions: *Tetrastemma* sp. SMOM019, *Siphonenteron* sp. SMOM059, and Lineidae gen. sp. SMOM092 were each subdivided into two BINs, while *Tetrastemma* spp. SMOM008 and SMOM020 were each subdivided into three BINs (Tables S1, S3, and Fig. 3).

**Figure 6:**
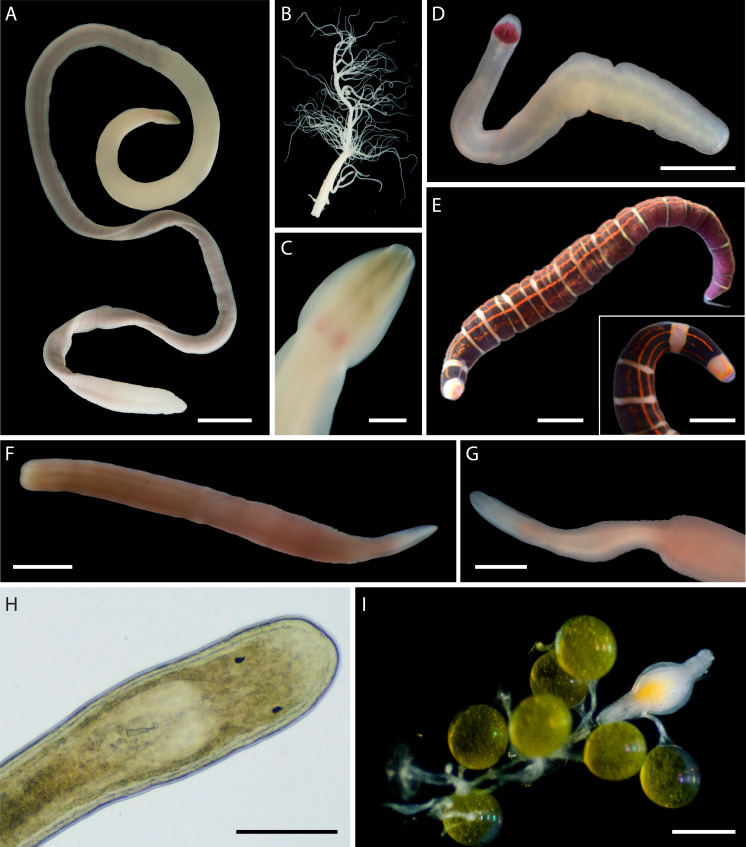
Distinct but not sequenced nemertean morphospecies from Oman. (A–C) *Polydendrorhynchus* sp. SMOM105, individual BOMAN-13795. (A) Entire specimen. (B) Expelled proboscis. (C) Dorsal view of head, showing pink cerebral ganglia. (D) *Tetrastemma* sp. SMOM107, individual BOMAN-09080. (E) ‘*Micrura’* sp. SMOM103 (*callima* species complex), individual BOMAN-08056. Inset shows dorsal view of head. (F–G) Lineidae gen. sp. SMOM104, individual BOMAN-11510. (H–I) *Carcinonemertes* sp. SMOM106 from an egg mass of *Leptodius exaratus*. (H) Dorsal view of head in transmitted light, compressed under coverglass, individual BOMAN-03188. (I) Individual BOMAN-03191, among the eggs of *L. exaratus*. Scale bars: A—1 cm; C—2 mm; D, I—0.5 mm; E and inset—1 mm; F, G—1.5 mm; H—0.25 mm.

In addition to the 102 MOTUs, we encountered at least five distinct morphospecies (Fig. 6), for which we were unable to obtain COI sequences. These unsequenced morphospecies were also assigned temporary alphanumeric names for tracking in the literature and public databases (Tables S2, S3, Fig. 6). This brings the known nemertean diversity of Oman to at least 107 putative species (including ASAP-delineated MOTUs and unsequenced morphospecies) or 114 prospective BINs (including 108 delineated BINs, 1 individual in a unique MOTU unplaced in a BIN, and 5 unsequenced morphospecies).

### Geographic distribution and differentiation

**Figure 7:**
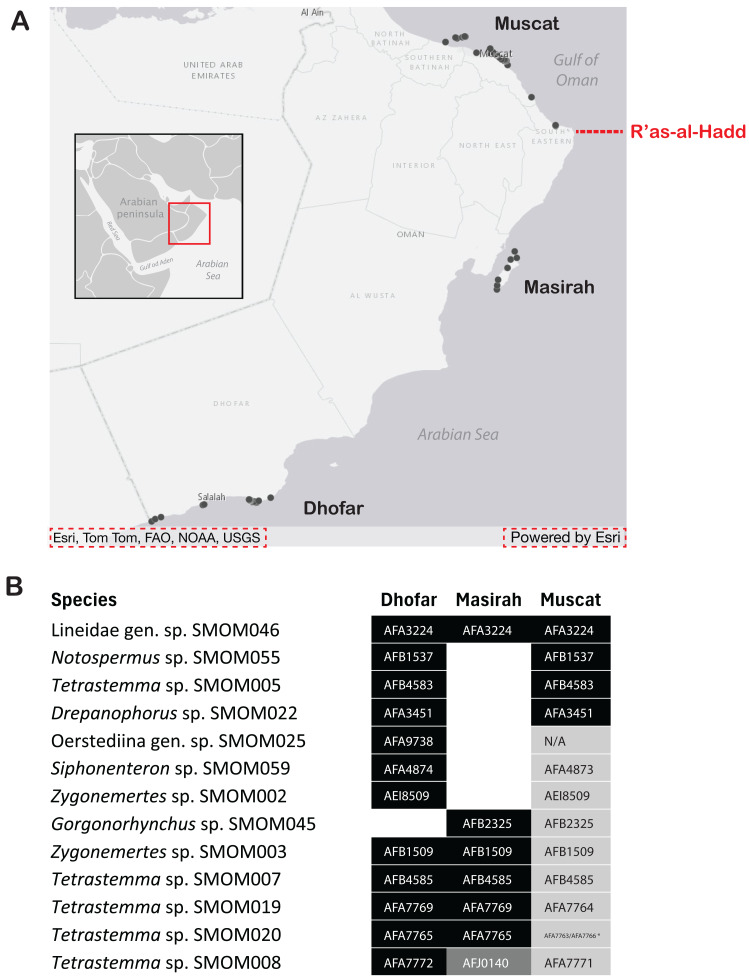
Geographic differentiation of nemertean species across the R’as-al-Hadd boundary. (A) Map of sampling locations, showing the location of the R’as-al-Hadd boundary (red dashed line) with respect to the three main sampling regions—Muscat (north of R’as-al-Hadd), Masirah Island and Dhofar (south of R’as-al-Hadd). (B) Thirteen of 107 nemertean species which occurred both in northern and southern Oman, showing genetic differentiation by region. White indicates lack of data. Shades of grey indicate reciprocally monophyletic clades below species level (numbers correspond to BOLD BINs). Oerstediina SMOM025 individual from Dhofar (BOMAN-8048) shows genetic differentiation from the individuals in Muscat large enough to be in a different BIN, but the sequence is not placed into a BIN (hence the N/A instead of BIN number). An asterisk (*) indicates an exception to the pattern of genetic differentiation by region within *Tetrastemma* sp. SMOM020: a single individual from Muscat (BOMAN-11554) is placed within an otherwise southern (Dhofar/Masirah) BIN (BOLD:AFA765). Map on inset has been modified from https://commons.wikimedia.org/wiki/File:BlankMap-World_gray.svg to crop and add labels under GNU Free Documentation License.

**Figure 8:**
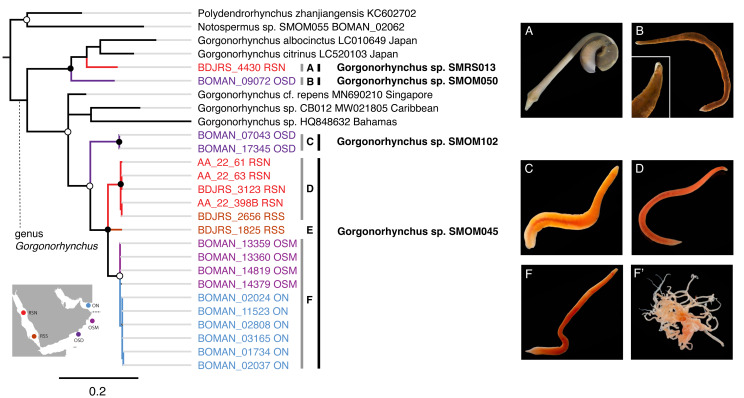
*Gorgonorhynchus* spp. from Arabia in a global context. Bayesian Inference tree based on the Cytochrome Oxidase I data, including reference sequences (in black). Fully supported clades are indicated by solid circles, those with posterior probability of >0.9—with open circles; clade support below 0.9 is not shown. Arabian clades are colored, with color matching sampling regions shown on the map: blue for Northern Oman, ON; purple for Dhofar in Southern Oman, OSD; magenta for Masirah Island in Southern Oman, OSM, scarlet red for Northern Red Sea, RSN; darker red for Southern Red Sea, RSS. Grey vertical lines correspond to BOLD BINs (A–D, F). Lineage E is not placed into a BIN. Correspondingly labeled panels of photographs of representative live specimens are on the right (A–F). No photographs were available for lineage E. (F’) proboscis of an individual from lineage F. Black vertical lines indicate MOTUs with corresponding temporary names. Note that reference sequence *Gorgonorhynchus* sp. HQ848632 from the Bahamas appears misidentified as belonging to a reptant polystiliferan hoplonemertean in the original publication (Andrade et al., 2012). GenBank accession numbers are shown for previous published sequences from other studies. Sequences from this study are shown with their field numbers and sampling region (see Table S1 for GenBank accession numbers).

We found little overlap between the fauna of northern and southern Oman—13 out of 107 putative species (12%) occurred in both regions (Table S3, Fig. 7). Excluding 54 singletons, since they cannot be found in more than one region, there was a 24% overlap between northern and southern species. A total of 57 putative species were found in northern Oman and 63 in southern Oman. Of the 13 MOTUs found in both northern and southern Oman, four contained multiple BINs segregated by geography (Fig. 7B): *Siphonenteron* sp. SMOM059, *Tetrastemma* sp. SMOM019, *Tetrastemma* sp. SMOM020, and *Tetrastemma* sp. SMOM008. Only nine of 114 prospective BINs (8%), as defined above, were found in both regions (Table S3), and four of these contained reciprocally monophyletic northern and southern lineages: *Tetrastemma* sp. SMOM007 (Fig. 3), *Zygonemertes* spp. SMOM002 and SMOM003 (Fig. 4), and *Gorgonorhynchus* sp. SMOM045 (Fig. 8). See also Fig. 7B, where reciprocally monophyletic lineages (including those within BINs) are indicated by different shades of grey. Additionally, Oerstediina sp. SMOM025 exhibited this pattern, albeit the southern variant was represented by a single sequence and was not placed in a BIN (Table S3, Fig. 7B). Thus, only four putative species (Lineidae gen. sp. SMOM046, *Notospermus* sp. SMOM055, *Tetrastemma* sp. SMOM005, and *Drepanophorus* sp. SMOM022) were shared by both regions without apparent differentiation (Fig. 7B).

Within the five multi-BIN MOTUs in the dataset, BINs corresponded to geographically segregated lineages (North-South), except for Lineidae gen. sp. SMOM092 (both BINs in southern Oman) and one individual of *Tetrastemma* sp. SMOM020 (BOMAN-11554) which was found in northern Oman, but was a part of an otherwise southern BIN (Fig. 3). Given its intermediate phenotype (Fig. 3T’) between the northern (Figs. 3R, 3S) and southern clades (Fig. 3T), this individual could represent a hybrid, with a northern father, and a southern mother, or simply reflect phenotypic plasticity.

Four cryptic species complexes (and thus likely close relatives) exhibited a pattern of geographic differentiation where one lineage had a range extending beyond Oman, while its sibling was restricted to Dhofar. One such case included *Baseodiscus* sp. SMOM101, collected only in Dhofar (Fig. 2C, Table S3), where it co-occurred with its look-alike and sibling, *B. hemprichii* (Fig. 2E), which has a wide distribution in the IWP (Table 1). Another case included a pair of newly delineated MOTUs from the *Gorgonorhynchus repens* species complex (Fig. 8). *Gorgonorhynchus* sp. SMOM045 is among the most common macroscopic nemerteans in Arabian waters, and we collected multiple specimens from Northern Oman, Masirah Island (southern Oman), and Red Sea (Tables S1, S3, Table 1, Figs. 8D, 8F), while its sibling, *Gorgonorhynchus* sp. SMOM102 was only found in Dhofar (Fig. 8C, Tables S1, S3). A third case comprised the circumtropical *Diplomma serpentinum* collected only in northern Oman (Tables S1, S3, and Table 1), and its look-alike, *Diplomma* cf. *albimarginatum* (SMOM027) only known from Dhofar (Tables S1, S3), although, as its name suggests, it may represent a previously described species from Madagascar (Kirsteuer, 1965). *Siphonenteron* sp. SMOM059 included two BINS, one of which (BOLD:AFA4873) is found in northern Oman and Vietnam (Tables S1, S3, and Table 1), while the other (BOLD:AFA4874) is only known in Dhofar (Tables S1, S3).

**Table 1:** Nemertean BOLD BINs in Oman with confirmed distribution in other parts of the world.

**BIN**	**Species**	**Distribution outside Oman**	**Sources**
ACA9932	*Bilucernus caputornatus*	wide IWP, WA	Ikenaga et al. (2025)
ACQ1696	*Diplomma serpentinum*	wide IWP distribution, WA, EP	Kajihara et al. (2011), Maslakova et al. (2022)
ACQ5911	*Cephalothrix* sp. SMOM035	wide IWP distribution, WA	Leasi & Norenburg (2014), Chernyshev & Polyakova (2021), Norenburg et al. (2024) (unpublished record in GenBank)
ADW6007	*Baseodiscus hemprichii*	wide IWP distribution	Kajihara & Hookabe (2019); Kajihara, Abukawa & Chernyshev, (2022)
AEI8509	*Zygonemertes* sp. SMOM002	Guam and WA	Maslakova et al. (2022)
AFA3451	*Drepanophorus* sp. SMOM022	Red Sea	S. Maslakova, 2025, unpublished data
AFA4481	Lineidae gen. sp. SMOM058	Guam	S. Maslakova, 2025, unpublished data
AFA4873	*Siphonenteron* sp. SMOM059^a^	Vietnam	Chernyshev et al. (2017)
AFA7764	*Tetrastemma* sp. SMOM019	Red Sea	S. Maslakova, 2025, unpublished data
AFB0485	Lineidae gen. sp. SMOM067	Guam	S. Maslakova, 2025, unpublished data
AFB1537	*Notospermus* sp. SMOM055	Red Sea	S. Maslakova, 2025, unpublished data
AFB1538	*Notospermus* sp. SMOM056	Red Sea	S. Maslakova, 2025, unpublished data
AFJ0636	*Baseodiscus* cf. *insignis*	Red Sea	S. Maslakova, 2025, unpublished data

**Notes.**

EP tropical East Pacific IWP Indo-West Pacific WA tropical West Atlantic

a *Siphonenteron* sp. SMOM059 (BOLD:AFA4873) is nearly identical (99.7%) to a sequence of “*Siphonenteron* cf. *bilineatum*” (KY561816) from Vietnam, which is not currently in BOLD.

On the phylogeny of *Carinoma*, a genus of infaunal palaeonemerteans whose species are mostly morphologically indistinguishable (except *Carinoma caraibicum* Stiasni-Wijnhoff, 1925), three out of five Omani MOTUs formed a well-supported clade (Fig. 5). Two of these, *Carinoma* sp. SMOM075 and *Carinoma* sp. SMOM081 were sympatric in northern Oman, while *Carinoma* sp. SMOM090 was found only in Dhofar. However, the divergences among these lineages are comparable to those from species in other geographic regions (12%), and it is possible that better sampling of other parts of the world will reveal that each Omani MOTU has a sister lineage elsewhere, rather than representing local differentiation in Oman. Similarly, in the genus *Zygonemertes*, two of the four Omani MOTUs, *Zygonemertes* sp. SMOM001 (found in Northern Oman) and SMOM004 (found in Southern Oman), represent sister lineages among current global samples of the genus, but the divergences are deep, and the support for this clade is low (Fig. 4). Thus, these lineages of *Carinoma* and *Zygonemertes* appear unlikely to represent local differentiation.

### Estimates of source diversity

Half of the 107 putative species were represented by a single individual (singletons), and 13 by only two (doubletons) (Table S3). Based on these numbers, the bias-corrected Chao1 estimated a source diversity of 209 species.

Given that such estimates are sensitive to uneven sampling across habitats, we also calculated Chao1 by habitat, coarsely divided into “hard bottom” (which included “sweats” of mass samples of coral rubble, vermetid-coralline reefs, algal mats and holdfasts, and barnacle-oyster assemblage, as well as animals found under rocks), and “soft bottom” (which included truly infaunal worms found within sand or mud, dug up intertidally with a shovel or yabby pump, or sieved from sand while SCUBA diving). Classified in this way, we documented 70 species (29 singletons) from hard bottom, and 37 (25 singletons) from soft bottom (Table S3). The Chao1 formula estimated 107 hard-bottom and 97 soft-bottom species (204 total).

### Undescribed and cryptic diversity

Only six of the 107 putative species (6%) could be confidently assigned to previously described species (Table S2), and three of those were recently described from Oman by our group—*Tetranemertes paulay*, *Tetranemertes arabica*, and *Tetranemertes unistriata* (Cherneva et al., 2023). The remaining three described species, *Diplomma serpentinum* (Stimpson, 1955)*, Baseodiscus hemprichii* (Ehrenberg, 1831), and *Bilucernus caputornatus* (Takakura, 1898) have wide distribution in tropical waters (Table 1).

Seven other putative species resemble, and could potentially represent, previously described, morphologically recognizable species (*i.e.,* not part of genera where most species are cryptic) from other parts of the IWP, but sequences from the type regions are not available for comparison, and evidence of cryptic species exists for some of those (indicated by an asterisk): *Baseodiscus insignis* Punnet and Cooper, 1909 from Zanzibar; *Tetranemertes rubrolineata (Kirsteuer, 1965), *Nipponnemertes madagascarensis* (Kirsteuer, 1965), and *Diplomma albimarginatum* (Kirsteuer, 1965) from Madagascar; *Tubulanus aureus (Joubin, 1904) and *Cerebratulus krempfi Joubin, 1904 from Djibouti; and *Eousia verticivaria Gibson, 1990 from Hong Kong. Thus, at least 95 (89%), but, possibly, as many as 105 (98%) of the nemertean species in Oman were undescribed until our recent work there.

Noteworthily, 92 (86%) of the putative species are cryptic, *i.e.,* have at least one known look-alike (Table S3). Many of these belong to genera notorious for morphological uniformity (*e.g.*, *Carinoma*, Fig. 5), where most species cannot be differentiated without genetic data. However, we found cryptic lineages even among what were previously thought to be morphologically distinct species. These include three of the best known, largest, most distinctive, and most frequently identified IWP nemerteans: *Baseodiscus hemprichii* (Ehrenberg, 1831)*, Notospermus tricuspidatus* (Quoy & Gaimard, 1833), and *Gorgonorhynchus repens* Dakin and Fordham, 1931. The first (Fig. 2E), a species with a striking color pattern, and wide IWP distribution (Table 1), was found to have a co-occurring undescribed look-alike, *Baseodiscus* sp. SMOM101 in Dhofar (Fig. 2C). The second was discovered to have a cryptic Arabian lineage (*Notospermus* sp. SMOM055, Fig. 2B), so far only known from Oman and the Red Sea, and deeply divergent from *N. tricuspidatus* from the type locality Guam (Jung, Maslakova & Lasley, 2025). Finally, a common Arabian species resembling *Gorgonorhynchus repens* Dakin and Fordham, 1931, was found to contain two distinct lineages in Oman, and two additional lineages in the Red Sea (Fig. 8), related to but distinct from the *Gorgonorhynchus* cf. *repens* previously reported from Japan and Singapore (Hookabe et al., 2021; Ip et al., 2019). Sequences from the type locality (New South Wales, Australia) are not available to test if any of these correspond to the true *G. repens*.

### Endemicity

Of the 114 putative BINs only 13 (11%) are currently known to occur outside Oman (Table 1). Those include the three species that were described prior to our recent work there—*Bilucernus caputornatus*, *Diplomma serpentinum*, and *Baseodiscus hemprichii*,—all of which have wide IWP distribution, some also occurring in the tropical West Atlantic and East Pacific. Two other species, *Cephalothrix* sp. SMOM035 and *Zygonemertes* sp. SMOM002, show a wide distribution in warm waters. The other eight are so far only known from a single other location within the IWP, and five of those are restricted to Arabian waters (Oman and Red Sea). Notably, only five of 13 have been reported by others, while the majority were documented by our recent sampling efforts (Maslakova et al., 2022; Ellison & Maslakova, 2023; Kahn, Wong & Maslakova, 2024; Jung, Maslakova & Lasley, 2025, and S. Maslakova, 2025, unpublished).

## Discussion

### Species diversity

This first sampling effort of nemerteans in Oman revealed 107 putative species, but the actual diversity is likely considerably higher, given the limited geographic, habitat, and depth coverage, and that half the species were rare (represented by a single specimen). Sampling was uneven along the coast and across habitats, with only two areas (Muscat and Dhofar) deeply sampled by a nemertean specialist (SM). We focused sampling on subtidal, hard bottom coral communities, particularly by extracting animals from dead coral rubble, and devoted much less effort to soft-bottom habitats which also harbor rich, but different assemblages of worms. Sampling was further limited to depths readily accessible at low tide or by SCUBA (mostly <30 m). We also did not sample the water column for holopelagic species, or planktonic larvae of benthic species. Sampling these types of habitats will likely reveal many more species.

While Chao1 predicts a source diversity between 204–209 species, the actual diversity is likely even higher, because this index tends to underestimate species richness, especially at low sampling intensity and when sampling is uneven across habitats and areas. At low sampling intensities, the magnitude of estimates correlates with sample size (Lemos et al., 2011), so estimates of species richness can be futile when sampling is insufficient (Bent & Forney, 2008). The addition of other areas and habitats would substantially increase documented diversity.

Our previous comparisons between benthic adult and planktonic larval faunas of nemerteans in three different geographic areas revealed that sampling larval stages from plankton can increase documented diversity by as much as 60% (Maslakova et al., 2022). These estimates and considerations suggest that the diversity of Omani nemerteans exceeds 300 species. In addition to focusing on sampling plankton and soft sediments (particularly in southern Oman), future sampling efforts should cover areas not covered by our survey.

The nemertean fauna of Oman is thus at least as diverse as that of other parts of the world recently surveyed by us (Maslakova et al., 2022). We found 102 species among 299 barcoded nemertean specimens in Oman. Limiting comparison to adults only, because we did not sample planktonic larvae in Oman, the Panama Bight ecoregion contained 74–78 putative species among 257 barcoded specimens, and the Southwestern Caribbean ecoregion contained 145 species among 587 barcoded individuals, while in Oregon 69 species are represented by 250 sequenced adults (Maslakova et al., 2022; Ellison & Maslakova, 2023; S. Maslakova, 2025, unpublished data).

The high diversity and the large fraction (89–98%) of previously undocumented nemerteans in Oman predict a much higher diversity in the wider IWP region. Our results underscore how poorly known this phylum is in the most diverse marine region in the world. Prior to our effort, only 217 benthic marine nemertean species have been documented from the entire IWP. Remarkably, our estimate of nemertean diversity in Oman alone exceeds this. Our limited survey boosts IWP diversity by 43%, with only three described species in common with other areas of the region (Table 1). Perhaps even more striking is that the Omani collections amount to 8% of the described global diversity of this phylum (107/1353 accepted species, Norenburg et al., 2024)—a testament to how poorly sampled and described nemerteans are worldwide.

The increase in the number of genetically characterized species is even more striking. Because of the prevalence of cryptic species, DNA taxonomy has become essential for nemertean species delineation, identification, and understanding of their distribution. The number of nemertean BINs (or equivalents) currently documented with DNA barcodes (1230) is comparable to the number of accepted species (1353). The 102 species and 108 BINs delineated in this study of Oman nemerteans represent a 100% increase in the number of DNA barcoded lineages from the entire IWP (106 prior to this study). The proportion of documented species that are represented in the DNA barcode libraries in BOLD and GenBank is another good indication of how well known a taxon is in an area. Only 5% (5 of 108) of the BINs encountered in Oman match other samples in BOLD or GenBank, not including samples from our recent unpublished work in the Red Sea and Guam (Table 1).

This study, as well as our other recent sampling efforts in East Pacific and West Atlantic (Maslakova et al., 2022; Ellison & Maslakova, 2023), Red Sea (Kahn, Wong & Maslakova, 2024), and Guam (Jung, Maslakova & Lasley, 2025) underscore the global undersampling of nemerteans, with the fraction of undescribed species ranging between 87–99%, depending on the region. At the same time, the fraction of all described nemertean species that have been DNA barcoded is ∼18%, while only ∼20% of all barcoded species are described. Together this suggests that 80–90% of nemertean species remain undescribed and undiscovered, with an estimated global diversity between 6,765 and 13,530 species. These numbers are based mostly on littoral and shallow-subtidal lineages. Sampling of deeper parts of the ocean would likely uncover additional species.

### Challenges of nemertean systematics

Almost a quarter of the species (23%) from this survey could not be assigned to a genus. Most of those (20) belong to the heteronemertean family Lineidae, characterized by the presence of lateral cephalic slits. The family is in a desperate need of revision, with the three largest genera, *Lineus*, *Micrura*, and *Cerebratulus*, which contain 71% of the species, known to be highly polyphyletic (see Kajihara, Ganaha & Kohtsuka, 2022 for a recent treatment). Some species could not be assigned even to the family level (Oerstediina gen. sp. SMOM025 and SMOM028, Heteronemertea gen. sp. SMOM079, and Pilidiophora gen. sp. SMOM093), because definitions of many families are morphologically vague and molecularly unsupported, and no close relatives have been DNA-barcoded. Obtaining sequences of more conservative markers than COI will help to place these taxa on the phylogeny.

Most (86%) nemertean species in Oman are cryptic (Table S3). The species of the genus *Carinoma* exemplify the challenge. Members of this genus lack color patterns, tend to be uniformly whitish, lack discernible external characters such as eyes or cephalic furrows, and, as palaeonemerteans, lack stylets, leaving little basis for morphological differentiation of species. There are ten described species of *Carinoma* worldwide, but there are 19 putative species among 68 available sequences. Most of those are undescribed, and the three described, sequenced species, the West Atlantic *Carinoma tremaphoros*, the East Pacific *Carinoma mutabile*, and the trans-Pacific *Carinoma hamanako*, each represent species complexes (Fig. 5). In fact, most species of *Carinoma* look alike. This is unsurprising given that these species are blind and infaunal (inhabit intertidal and subtidal soft sediments), so there is likely no selection for visual cues for species recognition. The situation is analogous for the palaeonemertean genera *Cephalothrix* and *Cephalotrichella*, the hoplonemertean genus *Ototyphlonemertes*, as well as many lineids, all of which contain numerous infaunal, feature-poor, cryptic, and undescribed species.

Given the large number of undescribed and cryptic species it is imperative to include sequence data, especially of rapidly evolving DNA barcoding markers, in species descriptions. Because histological characters are rarely useful for differentiating closely related species, but external appearance and stylets (where present) can be diagnostic, species descriptions should focus on the latter. Moving away from histological descriptions also expedites species descriptions, a much-needed change because nemertean diversity is high and mostly undescribed. Only through the study of living material combined with sequence data can a robust foundation be created for nemertean systematics, biogeography, and evolution.

### Endemicity and regional differentiation

The nemertean fauna shows strong differentiation between northern Oman (Muscat) and southern Oman (Dhofar and Masirah), based on the presence and absence of region-specific taxa and a small fraction of overlapping lineages (Fig. 7, Table S3). This suggests the presence of a barrier to dispersal, selection for differential survival and adaptation, or both. The fact that most species found in both regions exhibit subtle genetic differentiation indicates a barrier to dispersal between Muscat and Masirah, likely corresponding to the R’as-al-Hadd biogeographic boundary. The presence of such a boundary suggests that Oman may be a region of local differentiation and endemism. Preliminary observations suggest that the Dhofar region, in particular, harbors many unique lineages not found elsewhere. Dhofar is well-known for its endemic biota even within Arabia (Randall & Hoover, 1995; Schils & Wilson, 2006; Paulay & Meyer, 2025).

It is not currently possible to estimate the level of endemicity of nemertean fauna of Oman because so little of the IWP nemertean fauna has been sampled. However, it is noteworthy that five of the 13 BINs documented outside Oman are restricted to Arabia (Oman and Red Sea). The high proportion of species shared with the Red Sea reflects its proximity and biotic affinity. Preliminary observations based on our sampling in the Red Sea suggest that many species in Oman have their closest relatives there. Included in this study are data on *Tetrastemma* (Fig. 3) and *Gorgonorhynchus* (Fig. 8), but many other nemertean taxa show this pattern, supporting the notion that waters surrounding the Arabian Peninsula serve as an arena of speciation and a hotspot for marine biodiversity (Stuart-Smith et al., 2013; Di Battista et al., 2016). Furthermore, 69% of the species that were sampled in both northern and southern Oman showed differentiation between them, suggesting that the range of many species is narrow.

## Conclusions

This study of ribbon worms in Oman illustrates a large gap in our knowledge of marine invertebrate diversity, with as many as 90% of the species remaining to be discovered. It underscores the importance of sampling the most biodiverse regions of the world (the tropics), in general, and the IWP region, specifically. Most nemertean species are cryptic, which makes DNA barcodes essential for characterizing all previously described and new species. The problem of cryptic speciation makes it particularly important to recollect and barcode described species from as close as possible to the type localities to pin down existing names to genetic lineages and uncover patterns in biogeographic distribution and speciation. The large number of newly discovered species necessitates rapid approaches to species descriptions, which should focus on characteristics of living specimens and DNA barcodes, eliminating time-consuming histological studies. In the meantime, coining unique and persistent temporary names allows for species tracking in publications and databases (Horton et al., 2024). The fact that a large fraction of the newly discovered species cannot be placed into genera highlights the need for revision of nemertean systematics based on robust phylogenies, with the family Lineidae being the highest priority. Sequencing of additional, more conservative markers than Cytochrome Oxidase I, is critical to evaluating the phylogenetic positions of species that cannot be assigned to a genus. This study highlights the diversity and distinctiveness of the marine invertebrate fauna of Oman, suggesting it is a high priority for conservation. Evidence of local differentiation further suggests that conservation should be done on a regional scale to protect the distinct faunas found on either side of the R’as-al-Hadd biogeographic boundary. While we revealed over 100 new species, our study suggests that the nemertean diversity of Oman remains greatly undersampled, and that future surveys focusing on soft-bottom habitats and planktonic larval stages might uncover twice as many more species. Marine invertebrate diversity remains largely unknown, particularly in the most diverse tropical regions, and biodiversity surveys are critical for documenting species composition, understanding ecosystem function, setting priorities for conservation, and establishing baselines for monitoring change.

##  Supplemental Information

10.7717/peerj.19438/supp-1 Supplemental Information 1 A neighbor-Joining Tree of cytochrome oxidase I sequences of nemerteans from Oman included in this study

10.7717/peerj.19438/supp-2 Supplemental Information 2 COI sequences of nemerteans from Oman

10.7717/peerj.19438/supp-3 Supplemental Information 3 Specimen data of all sequenced nemerteans from Oman Individuals listed by MOTU (delineated based on ASAP subsets), with corresponding species ID, BOLD BIN assignment, field number (BOMAN), BOLD ID, GenBank Accession, Florida Museum of Natural History catalog number (UF) and collecting site (shown on Fig. 1). Sites codes starting with “OMAN” are from 2020, all others—from 2022. Letters following the collection site number represent substations (different habitats or collection methods at the same site).

10.7717/peerj.19438/supp-4 Supplemental Information 4 Classification of nemertean species reported from Oman by this study

10.7717/peerj.19438/supp-5 Supplemental Information 5 Habitat and distribution data for nemertean species and BINs in Oman Species marked with an asterisk (*) lack sequence data and are identified based on external morphological characteristics. We use open nomenclature term “cf.” to indicate that the species in Oman resembles a previously described species from another area, but we lack sequence data from the type locality to confirm that these are indeed the same. “X” represents presence in the region. Abbreviations: B, habitat type; (H, hard bottom; S, soft bottom;), C, cryptic species; ON, Northern Oman; OSD, Dhofar (Southern Oman), and OSM, Masirah Island (Southern Oman); S, singleton, D, doubleton.

10.7717/peerj.19438/supp-6 Supplemental Information 6 A list of nemertean reference sequences used for phylogenetic context of Oman data Representative nemertean sequences (1 per BIN) from Oman, and those of other available genetically characterized nemertean taxa (BOLD BINs), plus additional sequences from GenBank, and our unpublished sequences from the Caribbean, Panama Bight, Red Sea, Guam, and Moorea. Unpublished sequences can be made available from the corresponding author upon a reasonable request.

## Additional Information and Declarations

### Field Study Permissions

The following information was supplied relating to field study approvals (i.e., approving body and any reference numbers): Specimens were collected and exported to the United States with permission from the Environment Authority of Oman.

### DNA Deposition

The following information was supplied regarding the deposition of DNA sequences: The unpublished reference sequences were only used for preliminary phylogenetic analysis (Table S4) and so were not required to be published with the article. Please contact the corresponding author for those sequences.

### Data Availability

The following information was supplied regarding data availability: The datasets containing sequences, traces, specimen photos, and detailed collecting information generated and/or analyzed during the current study are available at the Barcode of Life Datasystems (DS-GORW24, DS-TSTW24, DS-NOMAN), GenBank OQ321709 –OQ321723, PP834667 –PP834949, and PQ871000, and the Florida Museum of Natural History collection database (299 specimens, Table S1, https://specifyportal.floridamuseum.ufl.edu/iz/).
