# Supplementary figures and images for: A hundred species, mostly new—first assessment of ribbon worm diversity and distribution in Oman
Source: PeerJ. 2025 May 28;13:e19438. doi: 10.7717/peerj.19438 (PMC12126093; doi:10.7717/peerj.19438)

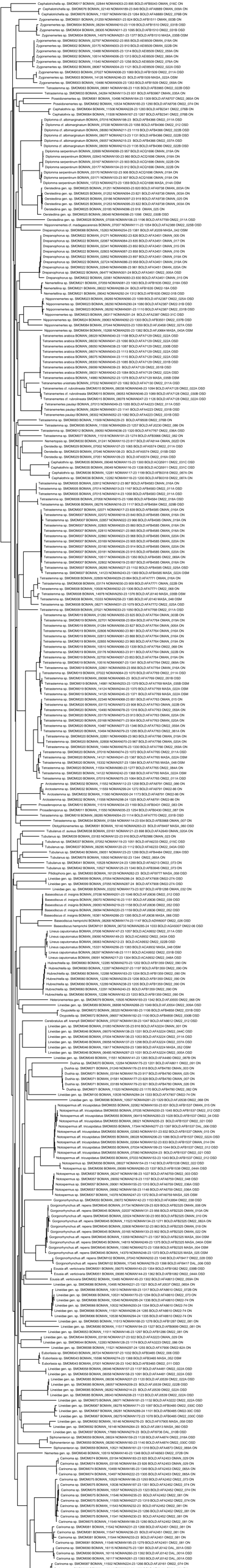

Supplement: Supplemental Information 1 [file peerj-13-19438-s001.pdf]
