# Supplementary material for: A hundred species, mostly new—first assessment of ribbon worm diversity and distribution in Oman
Source: PeerJ. 2025 May 28;13:e19438. doi: 10.7717/peerj.19438 (PMC12126093; doi:10.7717/peerj.19438)
Supplement: Supplemental Information 3 — Individuals listed by MOTU (delineated based on ASAP subsets), with corresponding species ID, BOLD BIN assignment, field number (BOMAN), BOLD ID, GenBank Accession, Florida Museum of Natural History catalog number (UF) and collecting site (shown on Fig. 1). Sites codes starting with “OMAN” are from 2020, all others—from 2022. Letters following the collection site number represent substations (different habitats or collection methods at the same site). [file peerj-13-19438-s003.docx]

**Supplemental Table 1. Specimen data of all sequenced nemerteans from Oman**. Individuals listed by MOTU (delineated based on ASAP subsets), with corresponding species ID, BOLD BIN assignment, field number (BOMAN), BOLD ID, GenBank Accession, Florida Museum of Natural History catalog number (UF) and collecting site (shown on Fig. 1). Sites codes starting with “OMAN” are from 2020, all others — from 2022. Letters following the collection site number represent substations (different habitats or collection methods at the same site).

| **Subset** | **Taxon ID** | **BOLD BIN** | **BOMAN** | **BOLD ID** | **Accession** | **UF** | **Collecting Site** |
| --- | --- | --- | --- | --- | --- | --- | --- |
| 1 | Zygonemertes sp. SMOM001 | AFB1511 | 1259 | NOMAN001-23 | PP834937 | 824 | OMAN-03B |
| 2 | Zygonemertes sp. SMOM002 | AEI8509 | 2797 | NOMAN002-23 | PP834939 | 855 | OMAN-18A |
| 2 | Zygonemertes sp. SMOM002 | AEI8509 | 3175 | NOMAN003-23 | PP834940 | 910 | OMAN-22B |
| 2 | Zygonemertes sp. SMOM002 | AEI8509 | 8067 | NOMAN004-23 | PP834938 | 1121 | OM22-22A |
| 2 | Zygonemertes sp. SMOM002 | AEI8509 | 10488 | NOMAN005-23 | PP834943 | 1314 | OM22-59A |
| 2 | Zygonemertes sp. SMOM002 | AEI8509 | 10514 | NOMAN006-23 | PP834942 | 1313 | OM22-69A |
| 2 | Zygonemertes sp. SMOM002 | AEI8509 | 11540 | NOMAN007-23 | PP834941 | 1256 | OM22-76A |
| 3 | Zygonemertes sp. SMOM003 | AFB1509 | 7027 | NOMAN008-23 | PP834945 | 1069 | OM22-11A |
| 3 | Zygonemertes sp. SMOM003 | AFB1509 | 10486 | NOMAN009-23 | PP834944 | 1353 | OM22-59A |
| 3 | Zygonemertes sp. SMOM003 | AFB1509 | 14126 | NOMAN246-23 | PP834946 | 1476 | MASA-22A |
| 4 | Zygonemertes sp. SMOM004 | AFB1510 | 8294 | NOMAN010-23 | PP834949 | 1109 | OM22-31B |
| 4 | Zygonemertes sp. SMOM004 | AFB1510 | 8305 | NOMAN011-23 | PP834948 | 1095 | OM22-31B |
| 4 | Zygonemertes sp. SMOM004 | AFB1510 | 14979 | NOMAN251-23 | PP834947 | 1377 | MASA-35B |
| 5 | Tetrastemma sp. SMOM005 | AFB4583 | 2812 | NOMAN012-23 | PP834866 | 867 | OMAN-16A |
| 5 | Tetrastemma sp. SMOM005 | AFB4583 | 7014 | NOMAN013-23 | PP834865 | 1167 | OM22-11A |
| 5 | Tetrastemma sp. SMOM005 | AFB4583 | 7015 | NOMAN014-23 | PP834864 | 1058 | OM22-11A |
| 6 | Tetrastemma sp. SMOM006 | AFB4584 | 7058 | NOMAN015-23 | PP834867 | 1066 | OM22-18A |
| 6 | Tetrastemma sp. SMOM006 | AFB4584 | 8276 | NOMAN016-23 | PP834868 | 1117 | OM22-29B |
| 7 | Tetrastemma sp. SMOM007 | AFB4585 | 2071 | NOMAN017-23 | PP834873 | 839 | OMAN-16A |
| 7 | Tetrastemma sp. SMOM007 | AFB4585 | 2072 | NOMAN018-23 | PP834878 | 840 | OMAN-16A |
| 7 | Tetrastemma sp. SMOM007 | AFB4585 | 2802 | NOMAN019-23 | PP834877 | 857 | OMAN-16A |
| 7 | Tetrastemma sp. SMOM007 | AFB4585 | 2805 | NOMAN020-23 | PP834874 | 860 | OMAN-16A |
| 7 | Tetrastemma sp. SMOM007 | AFB4585 | 2810 | NOMAN021-23 | PP834875 | 865 | OMAN-16A |
| 7 | Tetrastemma sp. SMOM007 | AFB4585 | 2857 | NOMAN022-23 | PP834876 | 966 | OMAN-19A |
| 7 | Tetrastemma sp. SMOM007 | AFB4585 | 2860 | NOMAN023-23 | PP834879 | 968 | OMAN-20A |
| 7 | Tetrastemma sp. SMOM007 | AFB4585 | 3169 | NOMAN024-23 | PP834880 | 905 | OMAN-20A |
| 7 | Tetrastemma sp. SMOM007 | AFB4585 | 3180 | NOMAN025-23 | PP834869 | 914 | OMAN-20A |
| 7 | Tetrastemma sp. SMOM007 | AFB4585 | 3181 | NOMAN026-23 | PP834870 | 915 | OMAN-20A |
| 7 | Tetrastemma sp. SMOM007 | AFB4585 | 8265 | NOMAN027-23 | PP834871 | 1102 | OM22-25A |
| 7 | Tetrastemma sp. SMOM007 | AFB4585 | 10517 | NOMAN028-23 | PP834872 | 1350 | OM22-69A |
| 7 | Tetrastemma sp. SMOM007 | AFB4585 | 14123 | NOMAN243-23 | PP834881 | 1369 | MASA-22A |
| 8 | Tetrastemma sp. SMOM008 | AFA7771 | 2809 | NOMAN029-23 | PP834887 | 864 | OMAN-16A |
| 8 | Tetrastemma sp. SMOM008 | AFA7771 | 3174 | NOMAN030-23 | PP834886 | 909 | OMAN-22B |
| 8 | Tetrastemma sp. SMOM008 | AFA7772 | 8271 | NOMAN031-23 | PP834882 | 1079 | OM22-25A |
| 8 | Tetrastemma sp. SMOM008 | AFA7771 | 10508 | NOMAN032-23 | PP834885 | 1306 | OM22-68 |
| 8 | Tetrastemma sp. SMOM008 | AFJ0140 | 14978 | NOMAN250-23 | PP834883 | 1376 | MASA-35B |
| 8 | Tetrastemma sp. SMOM008 | AFJ0140 | 15333 | NOMAN258-23 | PP834884 | 1385 | MASA-48 |
| 9 | Tetrastemma sp. SMOM009 | AFA7768 | 7021 | NOMAN033-23 | PP834888 | 1050 | OM22-11A |
| 10 | Prosadenoporus sp. SMOM010 | AFB0431 | 11519 | NOMAN034-23 | PP834858 | 1169 | OM22-83 |
| 11 | Prosadenoporus sp. SMOM011 | AFB0430 | 11550 | NOMAN035-23 | PP834859 | 1254 | OM22-87 |
| 12 | Tetrastemma sp. SMOM012 | AFA7767 | 9050 | NOMAN036-23 | PP834889 | 1320 | OM22-36A |
| 13 | Tetranemertes unistriata | AFA7130 | 7032 | NOMAN037-23 | OQ321719 | 1062 | OM22-11A |
| 15 | Tetranemertes arabica | AFA7129 | 8029 | NOMAN040-23 | OQ321711 | 1108 | OM22-22A |
| 15 | Tetranemertes arabica | AFA7129 | 8030 | NOMAN041-23 | OQ321712 | 1090 | OM22-22A |
| 15 | Tetranemertes arabica | AFA7129 | 8031 | NOMAN042-23 | OQ321716 | 1084 | OM22-22A |
| 15 | Tetranemertes arabica | AFA7129 | 8050 | NOMAN038-23 | OQ321721 | 1087 | OM22-3B |
| 15 | Tetranemertes arabica | AFA7129 | 8074 | NOMAN043-23 | OQ321717 | 1113 | OM22-22A |
| 15 | Tetranemertes arabica | AFA7129 | 8075 | NOMAN044-23 | OQ321710 | 1115 | OM22-22A |
| 15 | Tetranemertes arabica | AFA7129 | 8300 | NOMAN045-23 | OQ321709 | 1085 | OM22-31B |
| 15 | Tetranemertes arabica | AFA7129 | 9099 | NOMAN039-23 | OQ321713 | 1501 | OM22-51B |
| 15 | Tetranemertes arabica | AFA7129 | 14980 | NOMAN252-23 | PP834863 | 1378 | MASA-35B |
| 14 | Tetranemertes cf. rubrolineata | AFA7128 | 8038 | NOMAN048-23 | OQ321718 | 1094 | OM22-22A |
| 14 | Tetranemertes cf. rubrolineata | AFA7128 | 8053 | NOMAN046-23 | OQ321714 | 1089 | OM22-30B |
| 14 | Tetranemertes cf. rubrolineata | AFA7128 | 8078 | NOMAN047-23 | OQ321723 | 1126 | OM22-22A |
| 16 | Tetranemertes paulayi | AFA4223 | 7013 | NOMAN049-23 | OQ321715 | 1055 | OM22-11A |
| 16 | Tetranemertes paulayi | AFA4223 | 8284 | NOMAN051-23 | OQ321720 | 1141 | OM22-31B |
| 16 | Tetranemertes paulayi | AFA4223 | 8302 | NOMAN052-23 | OQ321722 | 1082 | OM22-31B |
| 17 | Cephalotrichella sp. SMOM017 | AFB5043 | 2844 | NOMAN053-23 | PP834707 | 895 | OMAN-16C |
| 18 | Tetrastemma sp. SMOM018 | AFA7770 | 8285 | NOMAN054-23 | PP834890 | 1114 | OM22-31B |
| 19 | Tetrastemma sp. SMOM019 | AFA7764 | 1260 | NOMAN055-23 | PP834903 | 825 | OMAN-3B |
| 19 | Tetrastemma sp. SMOM019 | AFA7764 | 1284 | NOMAN056-23 | PP834902 | 827 | OMAN-5A |
| 19 | Tetrastemma sp. SMOM019 | AFA7764 | 2700 | NOMAN057-23 | PP834901 | 853 | OMAN-19A |
| 19 | Tetrastemma sp. SMOM019 | AFA7764 | 2701 | NOMAN058-23 | PP834894 | 854 | OMAN-19A |
| 19 | Tetrastemma sp. SMOM019 | AFA7764 | 2801 | NOMAN059-23 | PP834892 | 856 | OMAN-16A |
| 19 | Tetrastemma sp. SMOM019 | AFA7764 | 2806 | NOMAN060-23 | PP834891 | 861 | OMAN-16A |
| 19 | Tetrastemma sp. SMOM019 | AFA7764 | 2813 | NOMAN061-23 | PP834906 | 868 | OMAN-16A |
| 19 | Tetrastemma sp. SMOM019 | AFA7764 | 2855 | NOMAN062-23 | PP834893 | 965 | OMAN-19A |
| 19 | Tetrastemma sp. SMOM019 | AFA7764 | 3176 | NOMAN063-23 | PP834904 | 911 | OMAN-22B |
| 19 | Tetrastemma sp. SMOM019 | AFA7769 | 7022 | NOMAN064-23 | PP834897 | 1070 | OM22-11A |
| 19 | Tetrastemma sp. SMOM019 | AFA7769 | 9098 | NOMAN065-23 | PP834898 | 1545 | OM22-51B |
| 19 | Tetrastemma sp. SMOM019 | AFA7764 | 10510 | NOMAN066-23 | PP834896 | 1339 | OM22-68 |
| 19 | Tetrastemma sp. SMOM019 | AFA7764 | 10516 | NOMAN067-23 | PP834905 | 1341 | OM22-69A |
| 19 | Tetrastemma sp. SMOM019 | AFA7769 | 14124 | NOMAN244-23 | PP834899 | 1370 | MASA-22A |
| 19 | Tetrastemma sp. SMOM019 | AFA7769 | 14125 | NOMAN245-23 | PP834900 | 1371 | MASA-22A |
| 19 | Tetrastemma sp. SMOM019 | AFA7769 | 14981 | NOMAN253-23 | PP834895 | 1379 | MASA-35B |
| 20 | Tetrastemma sp. SMOM020 | AFA7763 | 2348 | NOMAN068-23 | PP834908 | 851 | OMAN-15 |
| 20 | Tetrastemma sp. SMOM020 | AFA7766 | 2851 | NOMAN069-23 | PP834909 | 963 | OMAN-19A |
| 20 | Tetrastemma sp. SMOM020 | AFA7766 | 2858 | NOMAN070-23 | PP834910 | 967 | OMAN-20A |
| 20 | Tetrastemma sp. SMOM020 | AFA7763 | 3168 | NOMAN071-23 | PP834911 | 904 | OMAN-20A |
| 20 | Tetrastemma sp. SMOM020 | AFA7763 | 3172 | NOMAN072-23 | PP834912 | 908 | OMAN-22B |
| 20 | Tetrastemma sp. SMOM020 | AFA7763 | 3179 | NOMAN073-23 | PP834913 | 913 | OMAN-20A |
| 20 | Tetrastemma sp. SMOM020 | AFA7765 | 7018 | NOMAN074-23 | PP834914 | 1072 | OM22-11A |
| 20 | Tetrastemma sp. SMOM020 | AFA7765 | 7019 | NOMAN075-23 | PP834915 | 1064 | OM22-11A |
| 20 | Tetrastemma sp. SMOM020 | AFA7766 | 10484 | NOMAN076-23 | PP834916 | 1330 | OM22-59A |
| 20 | Tetrastemma sp. SMOM020 | AFA7763 | 10487 | NOMAN077-23 | PP834917 | 1346 | OM22-59A |
| 20 | Tetrastemma sp. SMOM020 | AFA7763 | 10490 | NOMAN078-23 | PP834918 | 1316 | OM22-59A |
| 20 | Tetrastemma sp. SMOM020 | AFA7763 | 10494 | NOMAN079-23 | PP834919 | 1295 | OM22-61A |
| 20 | Tetrastemma sp. SMOM020 | AFA7765 | 11554 | NOMAN080-23 | PP834920 | 1277 | OM22-84A |
| 20 | Tetrastemma sp. SMOM020 | AFA7765 | 14121 | NOMAN241-23 | PP834922 | 1367 | MASA-22A |
| 20 | Tetrastemma sp. SMOM020 | AFA7765 | 14122 | NOMAN242-23 | PP834907 | 1368 | MASA-22A |
| 20 | Tetrastemma sp. SMOM020 | AFA7765 | 15332 | NOMAN257-23 | PP834921 | 1384 | MASA-48 |
| 21 | Nemertellina sp. SMOM021 | AFB1635 | 7059 | NOMAN081-23 | PP834820 | 1063 | OM22-18A |
| 21 | Nemertellina sp. SMOM021 | AFB1635 | 8022 | NOMAN288-24 | PP834821 | 1544 | OM22-18A |
| 21 | Nemertellina sp. SMOM021 | AFB1635 | 9042 | NOMAN292-24 | PP834819 | 1312 | OM22-31B |
| 22 | Drepanophorus sp. SMOM022 | AFA3451 | 6477 | NOMAN301-24 | PQ871000 | 1030 | OM22-05A |
| 22 | Drepanophorus sp. SMOM022 | AFA3451 | 1271 | NOMAN082-23 | PP834725 | 826 | OMAN-05 |
| 22 | Drepanophorus sp. SMOM022 | AFA3451 | 2061 | NOMAN083-23 | PP834724 | 830 | OMAN-15 |
| 22 | Drepanophorus sp. SMOM022 | AFA3451 | 2067 | NOMAN084-23 | PP834731 | 835 | OMAN-17 |
| 22 | Drepanophorus sp. SMOM022 | AFA3451 | 2341 | NOMAN085-23 | PP834727 | 850 | OMAN-15 |
| 22 | Drepanophorus sp. SMOM022 | AFA3451 | 2724 | NOMAN086-23 | PP834730 | 958 | OMAN-19A |
| 22 | Drepanophorus sp. SMOM022 | AFA3451 | 2803 | NOMAN087-23 | PP834729 | 858 | OMAN-16 |
| 22 | Drepanophorus sp. SMOM022 | AFA3451 | 2849 | NOMAN088-23 | PP834728 | 961 | OMAN-20A |
| 22 | Drepanophorus sp. SMOM022 | AFA3451 | 2852 | NOMAN089-23 | PP834726 | 897 | OMAN-19A |
| 23 | Nipponnemertes sp. SMOM023 | AFA2387 | 8269 | NOMAN090-23 | PP834825 | 1099 | OM22-25A |
| 23 | Nipponnemertes sp. SMOM023 | AFA2387 | 8290 | NOMAN290-24 | PP834824 | 1080 | OM22-31B |
| 23 | Nipponnemertes sp. SMOM023 | AFA2387 | 8292 | NOMAN091-23 | PP834826 | 1110 | OM22-31B |
| 23 | Nipponnemertes sp. SMOM023 | AFA2387 | 8317 | NOMAN291-24 | PP834827 | 1494 | OM22-31C |
| 24 | Nipponnemertes sp. SMOM024 | AFB3551 | 9063 | NOMAN092-23 | PP834828 | 1303 | OM22-37B |
| 25 | Oerstediina gen. sp. SMOM025 | AFA9738 | 1251 | NOMAN093-23 | PP834749 | 820 | OMAN-03A |
| 25 | Oerstediina gen. sp. SMOM025 | AFA9738 | 1252 | NOMAN094-23 | PP834748 | 821 | OMAN-03A |
| 25 | Oerstediina gen. sp. SMOM025 | AFA9738 | 1253 | NOMAN095-23 | PP834747 | 822 | OMAN-03A |
| 25 | Oerstediina gen. sp. SMOM025 | N/A | 3185 | NOMAN096-23 | PP834746 | 918 | OMAN-25 |
| 25 | Oerstediina gen. sp. SMOM025 | AFA9738 | 3186 | NOMAN097-23 | PP834745 | 919 | OMAN-25 |
| 25 | Oerstediina gen. sp. SMOM025 | N/A | 8048 | NOMAN098-23 | PP834750 | 1098 | OM22-30B |
| 26 | Diplomma serpentinum | ACQ1696 | 2699 | NOMAN099-23 | PP834720 | 957 | OMAN-19A |
| 26 | Diplomma serpentinum | ACQ1696 | 2843 | NOMAN100-23 | PP834719 | 960 | OMAN-18A |
| 26 | Diplomma serpentinum | ACQ1696 | 3167 | NOMAN101-23 | PP834717 | 903 | OMAN-22B |
| 26 | Diplomma serpentinum | ACQ1696 | 3170 | NOMAN102-23 | PP834718 | 906 | OMAN-19A |
| 26 | Diplomma serpentinum | ACQ1696 | 3171 | NOMAN103-23 | PP834721 | 907 | OMAN-19A |
| 26 | Diplomma serpentinum | ACQ1696 | 3177 | NOMAN104-23 | PP834722 | 912 | OMAN-22B |
| 26 | Diplomma serpentinum | ACQ1696 | 13723 | NOMAN273-23 | PP834723 | 1359 | MASA-14A |
| 27 | Diplomma cf. albimarginatum | AFB4366 | 7016 | NOMAN198-23 | PP834713 | 1540 | OM22-11A |
| 27 | Diplomma cf. albimarginatum | AFB4366 | 7038 | NOMAN105-23 | PP834715 | 1056 | OM22-12 |
| 27 | Diplomma cf. albimarginatum | AFB4366 | 8059 | NOMAN210-23 | PP834711 | 1135 | OM22-22B |
| 27 | Diplomma cf. albimarginatum | AFB4366 | 8060 | NOMAN211-23 | PP834716 | 1119 | OM22-22B |
| 27 | Diplomma cf. albimarginatum | AFB4366 | 8077 | NOMAN213-23 | PP834712 | 1131 | OM22-22B |
| 27 | Diplomma cf. albimarginatum | AFB4366 | 9057 | NOMAN215-23 | PP834714 | 1497 | OM22-37A |
| 28 | Oerstediina gen. sp. SMOM028 | AFA7799 | 7008 | NOMAN106-23 | PP834751 | 1196 | OM22-11A |
| 29 | Oerstedia sp. SMOM029 | AFA5574 | 7002 | NOMAN107-23 | PP834849 | 1065 | OM22-11A |
| 29 | Oerstedia sp. SMOM029 | AFA5574 | 7046 | NOMAN108-23 | PP834850 | 1487 | OM22-15B |
| 29 | Oerstedia sp. SMOM029 | AFA5574 | 7051 | NOMAN109-23 | PP834851 | 1488 | OM22-16A |
| 30 | Nemertopsis sp. SMOM030 | AFA8144 | 1241 | NOMAN110-23 | PP834822 | 817 | OMAN-02D |
| 31 | Nipponnemertes cf. madagascarensis | AFA2388 | 7007 | NOMAN111-23 | PP834823 | 1054 | OM22-25B |
| 32 | Arctostemma sp. SMOM032 | AFA8791 | 11552 | NOMAN112-23 | PP834669 | 1258 | OM22-86 |
| 32 | Arctostemma sp. SMOM032 | AFA8791 | 11558 | NOMAN298-24 | PP834670 | 1325 | OM22-86 |
| 32 | Arctostemma sp. SMOM032 | AFA8791 | 11559 | NOMAN299-24 | PP834668 | 1272 | OM22-86 |
| 32 | Arctostemma sp. SMOM032 | AFA8791 | 11560 | NOMAN300-24 | PP834667 | 1173 | OM22-86 |
| 33 | Tetrastemma sp. SMOM033 | AFB3967 | 4284 | NOMAN113-23 | PP834923 | 931 | OMAN-35A |
| 34 | Tetrastemma sp. SMOM034 | AFB3968 | 1584 | NOMAN114-23 | PP834924 | 934 | OMAN-07 |
| 35 | Cephalothrix sp. SMOM035 | ACQ5911 | 9048 | NOMAN115-23 | PP834702 | 1300 | OM22-31C |
| 35 | Cephalothrix sp. SMOM035 | ACQ5911 | 9049 | NOMAN116-23 | PP834701 | 1338 | OM22-31C |
| 36 | Cephalothrix sp. SMOM036 | AFB0318 | 12281 | NOMAN117-23 | PP834704 | 1199 | OM22-87A |
| 36 | Cephalothrix sp. SMOM036 | AFB0318 | 12282 | NOMAN118-23 | PP834703 | 1200 | OM22-87A |
| 37 | Tubulanus sp. SMOM037 | AFA6233 | 7052 | NOMAN119-23 | PP834930 | 1051 | OM22-15C |
| 37 | Tubulanus sp. SMOM037 | AFA6233 | 8295 | NOMAN120-23 | PP834931 | 1112 | OM22-43A |
| 38 | Tubulanus cf. aureus SMOM038 | AFA2649 | 3161 | NOMAN121-23 | PP834929 | 898 | OMAN-2A |
| 39 | Tubulanus sp. SMOM039 | AFB2986 | 3183 | NOMAN122-23 | PP834932 | 916 | OMAN-23 |
| 40 | Tubulanus sp. SMOM040 | AFB4484 | 9051 | NOMAN123-23 | PP834933 | 1299 | OM22-36A |
| 41 | Tubulanus sp. SMOM041 | AFA6213 | 10528 | NOMAN124-23 | PP834934 | 1289 | OM22-73 |
| 42 | Tubulanus sp. SMOM042 | AFB3846 | 10527 | NOMAN125-23 | PP834935 | 1340 | OM22-73 |
| 43 | Euborlasia sp. SMOM043 | AFB3465 | 6724 | NOMAN197-23 | PP834743 | 1032 | OM22-6 |
| 43 | Euborlasia sp. SMOM043 | AFB3465 | 7001 | NOMAN126-23 | PP834742 | 1042 | OM22-11 |
| 43 | Euborlasia sp. SMOM043 | AFB3465 | 15596 | NOMAN274-23 | PP834744 | 1388 | MASA-52 |
| 44 | Lineidae gen. sp. SMOM044 | AFA3223 | 3190 | NOMAN127-23 | PP834774 | 922 | OMAN-29 |
| 44 | Lineidae gen. sp. SMOM044 | AFA3223 | 12283 | NOMAN128-23 | PP834775 | 1174 | OM22-86 |
| 45 | Gorgonorhynchus sp. SMOM045 | AFB2325 | 1734 | NOMAN129-23 | PP834760 | 829 | OMAN-08 |
| 45 | Gorgonorhynchus sp. SMOM045 | AFB2325 | 2024 | NOMAN130-23 | PP834761 | 955 | OMAN-10 |
| 45 | Gorgonorhynchus sp. SMOM045 | AFB2325 | 2037 | NOMAN131-23 | PP834759 | 956 | OMAN-10A |
| 45 | Gorgonorhynchus sp. SMOM045 | AFB2325 | 2808 | NOMAN132-23 | PP834758 | 863 | OMAN-18 |
| 45 | Gorgonorhynchus sp. SMOM045 | AFB2325 | 3165 | NOMAN133-23 | PP834757 | 902 | OMAN-22 |
| 45 | Gorgonorhynchus sp. SMOM045 | AFB2325 | 11523 | NOMAN134-23 | PP834752 | 1271 | OM22-82A |
| 45 | Gorgonorhynchus sp. SMOM045 | AFB2325 | 13359 | NOMAN271-23 | PP834753 | 1357 | MASA-04 |
| 45 | Gorgonorhynchus sp. SMOM045 | AFB2325 | 13360 | NOMAN272-23 | PP834754 | 1358 | MASA-04 |
| 45 | Gorgonorhynchus sp. SMOM045 | AFB2325 | 14379 | NOMAN248-23 | PP834756 | 1373 | MASA-25 |
| 45 | Gorgonorhynchus sp. SMOM045 | AFB2325 | 14819 | NOMAN249-23 | PP834755 | 1375 | MASA-40A |
| 46 | Lineidae gen. sp. SMOM046 | AFA3224 | 1083 | NOMAN135-23 | PP834781 | 816 | OMAN-01 |
| 46 | Lineidae gen. sp. SMOM046 | AFA3224 | 6485 | NOMAN267-23 | PP834776 | 1031 | OM22-05A |
| 46 | Lineidae gen. sp. SMOM046 | AFA3224 | 7004 | NOMAN136-23 | PP834779 | 1053 | OM22-11A |
| 46 | Lineidae gen. sp. SMOM046 | AFA3224 | 9058 | NOMAN137-23 | PP834777 | 1298 | OM22-37A |
| 46 | Lineidae gen. sp. SMOM046 | AFA3224 | 9079 | NOMAN138-23 | PP834780 | 1331 | OM22-44C |
| 46 | Lineidae gen. sp. SMOM046 | AFA3224 | 15617 | NOMAN259-23 | PP834778 | 1389 | MASA-52 |
| 47 | Cerebratulus sp. SMOM047 | AFA8612 | 7037 | NOMAN139-23 | PP834710 | 1047 | OM22-12 |
| 48 | Eopilidiidae gen. sp. SMOM048 | AFA6563 | 10519 | NOMAN140-23 | PP834765 | 1348 | OM22-72B |
| 49 | Lineidae gen. sp. SMOM049 | AFA4480 | 11551 | NOMAN141-23 | PP834782 | 1265 | OM22-87B |
| 50 | Gorgonorhynchus sp. SMOM050 | AFA3894 | 9072 | NOMAN142-23 | PP834764 | 1153 | OM22-38 |
| 51 | Eousia sp. SMOM051 | AFB1082 | 9075 | NOMAN143-23 | PP834739 | 1354 | OM22-39B |
| 51 | Eousia sp. SMOM051 | AFB1082 | 9085 | NOMAN144-23 | PP834740 | 1362 | OM22-44A |
| 52 | Eousia sp. SMOM052 | AFA8613 | 10485 | NOMAN145-23 | PP834741 | 1302 | OM22-59A |
| 53 | Lineidae gen. sp. SMOM053 | AFB1286 | 11511 | NOMAN146-23 | PP834783 | 1297 | OM22-81 |
| 54 | Bilucernus caputornatus | ACA9932 | 7006 | NOMAN147-23 | PP834816 | 1057 | OM22-11A |
| 54 | Bilucernus caputornatus | ACA9932 | 8063 | NOMAN212-23 | PP834818 | 1543 | OM22-22B |
| 54 | Bilucernus caputornatus | ACA9932 | 8297 | NOMAN148-23 | PP834815 | 1111 | OM22-31B |
| 54 | Bilucernus caputornatus | ACA9932 | 9090 | NOMAN149-23 | PP834813 | 1498 | OM22-43A |
| 54 | Bilucernus caputornatus | ACA9932 | 9091 | NOMAN217-23 | PP834817 | 1304 | OM22-48A |
| 54 | Bilucernus caputornatus | ACA9932 | 15331 | NOMAN256-23 | PP834814 | 1383 | MASA-48 |
| 55 | Notospermus sp. SMOM055 | AFB1537 | 2062 | NOMAN150-23 | PP834831 | 831 | OMAN-15 |
| 55 | Notospermus sp. SMOM055 | AFB1537 | 2063 | NOMAN151-23 | PP834841 | 832 | OMAN-15 |
| 55 | Notospermus sp. SMOM055 | AFB1537 | 2064 | NOMAN152-23 | PP834840 | 833 | OMAN-14 |
| 55 | Notospermus sp. SMOM055 | AFB1537 | 6419 | NOMAN283-23 | PP834832 | 1028 | OM22-04 |
| 55 | Notospermus sp. SMOM055 | AFB1537 | 7033 | NOMAN153-23 | PP834839 | 1043 | OM22-12 |
| 55 | Notospermus sp. SMOM055 | AFB1537 | 7034 | NOMAN199-23 | PP834838 | 1044 | OM22-12 |
| 55 | Notospermus sp. SMOM055 | AFB1537 | 7035 | NOMAN200-23 | PP834837 | 1045 | OM22-12 |
| 55 | Notospermus sp. SMOM055 | AFB1537 | 7060 | NOMAN204-23 | PP834836 | 1489 | OM22-21 |
| 55 | Notospermus sp. SMOM055 | AFB1537 | 8021 | NOMAN205-23 | PP834835 | 1490 | OM22-21 |
| 55 | Notospermus sp. SMOM055 | AFB1537 | 8028 | NOMAN206-23 | PP834834 | 1086 | OM22-22A |
| 55 | Notospermus sp. SMOM055 | AFB1537 | 17344 | NOMAN277-23 | PP834833 | 1397 | DAL-06 |
| 56 | Notospermus sp. SMOM056 | AFB1538 | 8027 | NOMAN154-23 | PP834843 | 1142 | OM22-22 |
| 56 | Notospermus sp. SMOM056 | AFB1538 | 9089 | NOMAN280-23 | PP834842 | 1337 | OM22-44A |
| 57 | Notospermus sp. SMOM057 | AFA6759 | 6247 | NOMAN196-23 | PP834844 | 1027 | OM22-03 |
| 57 | Notospermus sp. SMOM057 | AFA6759 | 9061 | NOMAN155-23 | PP834846 | 1315 | OM22-36A |
| 57 | Notospermus sp. SMOM057 | AFA6759 | 9062 | NOMAN156-23 | PP834847 | 1148 | OM22-36A |
| 57 | Notospermus sp. SMOM057 | AFA6759 | 9092 | NOMAN218-23 | PP834848 | 1157 | OM22-48 |
| 57 | Notospermus sp. SMOM057 | AFA6759 | 14378 | NOMAN247-23 | PP834845 | 1372 | MASA-25 |
| 58 | Lineidae gen. sp. SMOM058 | AFA4481 | 8046 | NOMAN157-23 | PP834785 | 1137 | OM22-22A |
| 58 | Lineidae gen. sp. SMOM058 | AFA4481 | 8058 | NOMAN158-23 | PP834784 | 1091 | OM22-22A |
| 59 | Siphonenteron sp. SMOM059 | AFA4874 | 8024 | NOMAN159-23 | PP834860 | 1128 | OM22-18A |
| 59 | Siphonenteron sp. SMOM059 | AFA4874 | 8280 | NOMAN160-23 | PP834862 | 1140 | OM22-30C |
| 59 | Siphonenteron sp. SMOM059 | AFA4873 | 10521 | NOMAN161-23 | PP834861 | 1318 | OM22-69A |
| 60 | Hubrechtella sp. SMOM060 | AFB1359 | 12285 | NOMAN270-23 | PP834768 | 1202 | OM22-90 |
| 60 | Hubrechtella sp. SMOM060 | AFB1359 | 12286 | NOMAN162-23 | PP834773 | 1203 | OM22-90 |
| 60 | Hubrechtella sp. SMOM060 | AFB1359 | 12287 | NOMAN237-23 | PP834767 | 1197 | OM22-90 |
| 60 | Hubrechtella sp. SMOM060 | AFB1359 | 12288 | NOMAN163-23 | PP834772 | 1204 | OM22-90 |
| 60 | Hubrechtella sp. SMOM060 | AFB1359 | 12289 | NOMAN238-23 | PP834771 | 1205 | OM22-90 |
| 60 | Hubrechtella sp. SMOM060 | AFB1359 | 12290 | NOMAN239-23 | PP834770 | 1206 | OM22-90 |
| 60 | Hubrechtella sp. SMOM060 | AFB1359 | 12291 | NOMAN240-23 | PP834769 | 1528 | OM22-90 |
| 61 | Poseidonemertes sp. SMOM061 | AFA8707 | 10496 | NOMAN164-23 | PP834856 | 1309 | OM22-65A |
| 62 | Poseidonemertes sp. SMOM062 | AFA8706 | 10534 | NOMAN165-23 | PP834857 | 1310 | OM22-74 |
| 63 | Tetrastemma sp. SMOM063 | AFB3965 | 8061 | NOMAN166-23 | PP834925 | 1311 | OM22-22B |
| 64 | Cephalothrix sp. SMOM064 | AFB2341 | 11506 | NOMAN228-23 | PP834705 | 1312 | OM22-76B |
| 64 | Cephalothrix sp. SMOM064 | AFB2341 | 11508 | NOMAN167-23 | PP834706 | 1313 | OM22-76B |
| 65 | Lineidae gen. sp. SMOM065 | AFB1287 | 11513 | NOMAN168-23 | PP834786 | 1314 | OM22-81 |
| 66 | Lineidae gen. sp. SMOM066 | AFA8610 | 10513 | NOMAN169-23 | PP834792 | 1315 | OM22-72B |
| 66 | Lineidae gen. sp. SMOM066 | AFA8610 | 10531 | NOMAN170-23 | PP834790 | 1316 | OM22-73 |
| 66 | Lineidae gen. sp. SMOM066 | AFA8610 | 10532 | NOMAN293-24 | PP834789 | 1317 | OM22-74 |
| 66 | Lineidae gen. sp. SMOM066 | AFA8610 | 10538 | NOMAN294-24 | PP834787 | 1318 | OM22-74 |
| 66 | Lineidae gen. sp. SMOM066 | AFA8610 | 10540 | NOMAN295-24 | PP834791 | 1319 | OM22-74 |
| 66 | Lineidae gen. sp. SMOM066 | AFA8610 | 11501 | NOMAN296-24 | PP834788 | 1320 | OM22-74 |
| 67 | Lineidae gen. sp. SMOM067 | AFB0485 | 8278 | NOMAN171-23 | PP834794 | 1321 | OM22-30C |
| 67 | Lineidae gen. sp. SMOM067 | AFB0485 | 8279 | NOMAN172-23 | PP834793 | 1322 | OM22-30C |
| 67 | Lineidae gen. sp. SMOM067 | AFB0485 | 8281 | NOMAN289-24 | PP834795 | 1323 | OM22-30C |
| 68 | Lineidae gen. sp. SMOM068 | AFB1288 | 3202 | NOMAN173-23 | PP834796 | 1324 | OMAN-32 |
| 69 | Baseodiscus hemprichii | ADW6007 | 8268 | NOMAN174-23 | PP834672 | 1325 | OM22-26 |
| 70 | Dushia sp. SMOM070 | AFA8611 | 12284 | NOMAN175-23 | PP834733 | 1326 | OM22-91 |
| 71 | Dushia sp. SMOM071 | AFB4780 | 1249 | NOMAN176-23 | PP834736 | 1327 | OMAN-03 |
| 71 | Dushia sp. SMOM071 | AFB4780 | 1581 | NOMAN177-23 | PP834737 | 1328 | OMAN-07 |
| 71 | Dushia sp. SMOM071 | AFB4780 | 3184 | NOMAN178-23 | PP834738 | 1329 | OMAN-25 |
| 71 | Dushia sp. SMOM071 | AFB4780 | 3189 | NOMAN179-23 | PP834734 | 1330 | OMAN-26 |
| 71 | Dushia sp. SMOM071 | AFB4780 | 11520 | NOMAN282-23 | PP834735 | 1331 | OM22-82 |
| 72 | Oxypolella sp. SMOM072 | AFB4858 | 8320 | NOMAN180-23 | PP834854 | 1332 | OM22-31B |
| 72 | Oxypolella sp. SMOM072 | AFB4858 | 8657 | NOMAN182-23 | PP834853 | 1333 | OM22-30B |
| 73 | Lineidae gen. sp. SMOM073 | AFA3222 | 8071 | NOMAN181-23 | PP834797 | 1334 | OM22-22A |
| 74 | Carinoma sp. SMOM074 | AFA2453 | 3194 | NOMAN183-23 | PP834681 | 1335 | OMAN-29 |
| 74 | Carinoma sp. SMOM074 | AFA2453 | 3195 | NOMAN184-23 | PP834679 | 1336 | OMAN-29 |
| 74 | Carinoma sp. SMOM074 | AFA2453 | 10497 | NOMAN222-23 | PP834678 | 1337 | OM22-65A |
| 74 | Carinoma sp. SMOM074 | AFA2453 | 10499 | NOMAN185-23 | PP834680 | 1338 | OM22-65A |
| 75 | Carinoma sp. SMOM075 | AFA2452 | 10529 | NOMAN186-23 | PP834687 | 1339 | OM22-73 |
| 75 | Carinoma sp. SMOM075 | AFA2452 | 10536 | NOMAN187-23 | PP834686 | 1340 | OM22-74 |
| 75 | Carinoma sp. SMOM075 | AFA2452 | 10537 | NOMAN223-23 | PP834688 | 1341 | OM22-74 |
| 75 | Carinoma sp. SMOM075 | AFA2452 | 11505 | NOMAN227-23 | PP834684 | 1342 | OM22-74 |
| 75 | Carinoma sp. SMOM075 | AFA2452 | 11541 | NOMAN230-23 | PP834683 | 1539 | OM22-81 |
| 75 | Carinoma sp. SMOM075 | AFA2452 | 11543 | NOMAN232-23 | PP834682 | 1524 | OM22-81 |
| 75 | Carinoma sp. SMOM075 | AFA2452 | 11545 | NOMAN234-23 | PP834690 | 1286 | OM22-81 |
| 75 | Carinoma sp. SMOM075 | AFA2452 | 11546 | NOMAN235-23 | PP834689 | 1526 | OM22-81 |
| 75 | Carinoma sp. SMOM075 | AFA2452 | 11549 | NOMAN188-23 | PP834685 | 1280 | OM22-81 |
| 76 | Cephalotrichella sp. SMOM076 | AFA8889 | 2149 | NOMAN189-23 | PP834708 | 849 | OMAN-09A |
| 76 | Cephalotrichella sp. SMOM076 | AFA8889 | 11507 | NOMAN190-23 | PP834709 | 1264 | OM22-76B |
| 77 | Tetrastemma sp. SMOM077 | AFB3966 | 11518 | NOMAN191-23 | PP834926 | 1274 | OM22-82 |
| 78 | Tubulanus sp. SMOM078 | N/A | 10500 | NOMAN192-23 | PP834936 | 1344 | OM22-65A |
| 79 | Heteronemertea gen. sp. SMOM079 | AFJ0555 | 10505 | NOMAN193-23 | PP834766 | 1342 | OM22-68 |
| 80 | Lineidae gen. sp. SMOM080 | AFB0699 | 11517 | NOMAN194-23 | PP834798 | 1327 | OM22-81 |
| 81 | Carinoma sp. SMOM081 | AFA2451 | 11542 | NOMAN231-23 | PP834692 | 1268 | OM22-81 |
| 81 | Carinoma sp. SMOM081 | AFA2451 | 11544 | NOMAN233-23 | PP834693 | 1525 | OM22-81 |
| 81 | Carinoma sp. SMOM081 | AFA2451 | 11547 | NOMAN236-23 | PP834691 | 1527 | OM22-81 |
| 81 | Carinoma sp. SMOM081 | AFA2451 | 11548 | NOMAN195-23 | PP834694 | 1279 | OM22-81 |
| 82 | Baseodiscus cf. insignis | AFJ0636 | 7036 | NOMAN201-23 | PP834677 | 1046 | OM22-12 |
| 82 | Baseodiscus cf. insignis | AFJ0636 | 9070 | NOMAN216-23 | PP834673 | 1151 | OM22-39 |
| 82 | Baseodiscus cf. insignis | AFJ0636 | 9093 | NOMAN219-23 | PP834674 | 1158 | OM22-52 |
| 82 | Baseodiscus cf. insignis | AFJ0636 | 9094 | NOMAN220-23 | PP834676 | 1159 | OM22-52 |
| 82 | Baseodiscus cf. insignis | AFJ0636 | 16381 | NOMAN266-23 | PP834675 | 1395 | MASA-66 |
| 88 | Tetrastemma sp. SMOM083 | AFI9508 | 11509 | NOMAN229-23 | PP834927 | 1486 | OM22-76B |
| 84 | Nipponnemertes sp. SMOM084 | AFJ0458 | 7044 | NOMAN203-23 | PP834829 | 1059 | OM22-27A |
| 85 | Lineidae gen. sp. SMOM085 | AFJ0539 | 8036 | NOMAN207-23 | PP834800 | 1120 | OM22-22A |
| 85 | Lineidae gen. sp. SMOM085 | AFJ0539 | 8043 | NOMAN208-23 | PP834801 | 1123 | OM22-22A |
| 85 | Lineidae gen. sp. SMOM085 | AFJ0539 | 8057 | NOMAN209-23 | PP834799 | 1492 | OM22-22B |
| 85 | Lineidae gen. sp. SMOM085 | AFJ0539 | 8262 | NOMAN214-23 | PP834802 | 1493 | OM22-22A |
| 86 | Lineidae gen. sp. SMOM086 | AFJ0537 | 10495 | NOMAN221-23 | PP834803 | 1321 | OM22-65A |
| 87 | Carinoma sp. SMOM087 | AFJ0161 | 11502 | NOMAN224-23 | PP834697 | 1266 | OM22-74 |
| 87 | Carinoma sp. SMOM087 | AFJ0161 | 11503 | NOMAN225-23 | PP834695 | 1270 | OM22-74 |
| 87 | Carinoma sp. SMOM087 | AFJ0161 | 11504 | NOMAN226-23 | PP834696 | 1275 | OM22-74 |
| 89 | Drepanophorus sp. SMOM088 | AFJ0209 | 15263 | NOMAN254-23 | PP834732 | 1381 | MASA-42 |
| 95 | Lineidae gen. sp. SMOM089 | AFJ0554 | 6898 | NOMAN268-23 | PP834804 | 1049 | OM22-09A |
| 91 | Carinoma sp. SMOM090 | AFJ0142 | 16115 | NOMAN275-23 | PP834699 | 1391 | DAL-01A |
| 91 | Carinoma sp. SMOM090 | AFJ0142 | 16116 | NOMAN260-23 | PP834698 | 1392 | DAL-01A |
| 91 | Carinoma sp. SMOM090 | AFJ0142 | 16117 | NOMAN261-23 | PP834700 | 1393 | DAL-01A |
| 93 | Ototyphlonemertes sp. SMOM091 | AFI9497 | 16140 | NOMAN263-23 | PP834852 | 1479 | MASA-60 |
| 94 | Lineidae gen. sp. SMOM092 | AFX7905 | 16146 | NOMAN276-23 | PP834806 | 1481 | MASA-58 |
| 94 | Lineidae gen. sp. SMOM092 | AFJ0613 | 16148 | NOMAN264-23 | PP834805 | 1483 | MASA-60 |
| 92 | Pilidiophora gen. sp. SMOM093 | AFI9777 | 16129 | NOMAN262-23 | PP834855 | 1477 | MASA-58 |
| 90 | Nipponnemertes sp. SMOM094 | AFJ0664 | 15268 | NOMAN255-23 | PP834830 | 1382 | MASA-43A |
| 96 | Tetrastemma sp. SMOM095 | AFJ0230 | 11556 | NOMAN269-23 | PP834928 | 1257 | OM22-86 |
| 98 | Lineidae gen. sp. SMOM096 | AFJ0538 | 10507 | NOMAN281-23 | PP834807 | 1329 | OM22-68 |
| 97 | Lineidae gen. sp. SMOM097 | AFI9736 | 17669 | NOMAN279-23 | PP834808 | 1485 | DAL-10B |
| 102 | Lineidae gen. sp. SMOM098 | AFX7906 | 11521 | NOMAN297-24 | PP834809 | 1255 | OM22-82A |
| 101 | Lineidae gen. sp. SMOM099 | AFX7908 | 7054 | NOMAN286-24 | PP834811 | 1256 | OM22-27A |
| 101 | Lineidae gen. sp. SMOM099 | AFX7908 | 7055 | NOMAN287-24 | PP834810 | 1257 | OM22-27A |
| 99 | Lineidae gen. sp. SMOM100 | AFX9707 | 10539 | NOMAN284-24 | PP834812 | 1333 | OM22-74 |
| 100 | Baseodiscus sp. SMOM101 | AGA5007 | 6733 | NOMAN285-24 | PP834671 | 1033 | OM22-06 |
| 83 | Gorgonorhynchus sp. SMOM102 | AFI9417 | 7043 | NOMAN202-23 | PP834763 | 1048 | OM22-28 |
| 83 | Gorgonorhynchus sp. SMOM102 | AFI9417 | 17345 | NOMAN278-23 | PP834762 | 1398 | DAL-06 |
