# Supplementary material for: A hundred species, mostly new—first assessment of ribbon worm diversity and distribution in Oman
Source: PeerJ. 2025 May 28;13:e19438. doi: 10.7717/peerj.19438 (PMC12126093; doi:10.7717/peerj.19438)
Supplement: Supplemental Information 4 [file peerj-13-19438-s004.docx]

**Supplemental Table 2. Classification of nemertean species reported from Oman by this study.**

| **class Palaeonemertea** | | | | | |  |
| --- | --- | --- | --- | --- | --- | --- |
|  | order Archinemertea | | | | |  |
|  |  |  |  | family Cephalotrichidae | | |
|  |  |  |  |  | genus Cephalothrix | |
|  |  |  |  |  |  | Cephalothrix sp. SMOM035 |
|  |  |  |  |  |  | Cephalothrix sp. SMOM036 |
|  |  |  |  |  |  | Cephalothrix sp. SMOM064 |
|  |  |  |  | family Cephalotrichellidae | | |
|  |  |  |  |  | genus Cephalotrichella | |
|  |  |  |  |  |  | Cephalotrichella sp. SMOM017 |
|  |  |  |  |  |  | Cephalotrichella sp. SMOM076 |
|  | order Carinomiformes | | | | |  |
|  |  |  |  | family Carinomidae | | |
|  |  |  |  |  | genus Carinoma | |
|  |  |  |  |  |  | Carinoma sp. SMOM074 |
|  |  |  |  |  |  | Carinoma sp. SMOM075 |
|  |  |  |  |  |  | Carinoma sp. SMOM081 |
|  |  |  |  |  |  | Carinoma sp. SMOM087 |
|  |  |  |  |  |  | Carinoma sp. SMOM090 |
|  | order Tubulaniformes | | | | |  |
|  |  |  |  | family Tubulanidae | | |
|  |  |  |  |  | genus Tubulanus | |
|  |  |  |  |  |  | Tubulanus cf. aureus |
|  |  |  |  |  |  | Tubulanus sp. SMOM037 |
|  |  |  |  |  |  | Tubulanus sp. SMOM039 |
|  |  |  |  |  |  | Tubulanus sp. SMOM040 |
|  |  |  |  |  |  | Tubulanus sp. SMOM041 |
|  |  |  |  |  |  | Tubulanus sp. SMOM042 |
|  |  |  |  |  |  | Tubulanus sp. SMOM078 |
| **class Pilidiophora** | | | | |  |  |
|  | order Heteronemertea | | | | |  |
|  |  |  |  | family Eopilidiidae | | |
|  |  |  |  |  | Eopilidiidae gen. sp. SMOM048 | |
|  |  |  |  | family Lineidae | | |
|  |  |  |  |  | genus Bilucernus | |
|  |  |  |  |  |  | Bilucernus caputornatus |
|  |  |  |  |  | genus Cerebratulus^a^ | |
|  |  |  |  |  |  | Cerebratulus sp. SMOM047 |
|  |  |  |  |  | genus Dushia | |
|  |  |  |  |  |  | Dushia sp. SMOM070 |
|  |  |  |  |  |  | Dushia sp. SMOM071 |
|  |  |  |  |  | genus Eousia | |
|  |  |  |  |  |  | Eousia sp. SMOM051 |
|  |  |  |  |  |  | Eousia sp. SMOM052 |
|  |  |  |  |  | genus Euborlasia | |
|  |  |  |  |  |  | Euborlasia sp. SMOM043 |
|  |  |  |  |  | genus Gorgonorhynchus | |
|  |  |  |  |  |  | Gorgonorhynchus sp. SMOM045 |
|  |  |  |  |  |  | Gorgonorhynchus sp. SMOM050 |
|  |  |  |  |  |  | Gorgonorhynchus sp. SMOM102 |
|  |  |  |  |  | genus Micrura^a^ | |
|  |  |  |  |  |  | Micrura sp. SMOM103 |
|  |  |  |  |  | genus Notospermus | |
|  |  |  |  |  |  | Notospermus sp. SMOM055 |
|  |  |  |  |  |  | Notospermus sp. SMOM056 |
|  |  |  |  |  |  | Notospermus sp. SMOM057 |
|  |  |  |  |  | genus Polydendrorhynchus | |
|  |  |  |  |  |  | Polydendrorhynchus sp. SMOM104 |
|  |  |  |  |  | genus Siphonenteron | |
|  |  |  |  |  |  | Siphonenteron sp. SMOM059 |
|  |  |  |  |  | genus incertae sedis | |
|  |  |  |  |  |  | Lineidae gen. sp. SMOM044 |
|  |  |  |  |  |  | Lineidae gen. sp. SMOM046 |
|  |  |  |  |  |  | Lineidae gen. sp. SMOM049 |
|  |  |  |  |  |  | Lineidae gen. sp. SMOM053 |
|  |  |  |  |  |  | Lineidae gen. sp. SMOM058 |
|  |  |  |  |  |  | Lineidae gen. sp. SMOM065 |
|  |  |  |  |  |  | Lineidae gen. sp. SMOM066 |
|  |  |  |  |  |  | Lineidae gen. sp. SMOM067 |
|  |  |  |  |  |  | Lineidae gen. sp. SMOM068 |
|  |  |  |  |  |  | Lineidae gen. sp. SMOM073 |
|  |  |  |  |  |  | Lineidae gen. sp. SMOM080 |
|  |  |  |  |  |  | Lineidae gen. sp. SMOM085 |
|  |  |  |  |  |  | Lineidae gen. sp. SMOM086 |
|  |  |  |  |  |  | Lineidae gen. sp. SMOM089 |
|  |  |  |  |  |  | Lineidae gen. sp. SMOM092 |
|  |  |  |  |  |  | Lineidae gen. sp. SMOM096 |
|  |  |  |  |  |  | Lineidae gen. sp. SMOM097 |
|  |  |  |  |  |  | Lineidae gen. sp. SMOM098 |
|  |  |  |  |  |  | Lineidae gen. sp. SMOM099 |
|  |  |  |  |  |  | Lineidae gen. sp. SMOM100 |
|  |  |  |  |  |  | Lineidae gen. sp. SMOM104 |
|  |  |  |  | family Valenciniidae | | |
|  |  |  |  |  | genus Baseodiscus | |
|  |  |  |  |  |  | Baseodiscus cf. insignis |
|  |  |  |  |  |  | Baseodiscus hemprichii |
|  |  |  |  |  |  | Baseodiscus sp. SMOM101 |
|  |  |  |  |  | genus Oxypolella | |
|  |  |  |  |  |  | Oxypolella sp. SMOM072 |
|  |  |  |  | family incertae sedis | | |
|  |  |  |  |  | genus insertae sedis | |
|  |  |  |  |  |  | Heteronemertea gen. sp. SMOM079 |
|  | order Hubrechtiiformes | | | | | |
|  |  |  |  | family Hubrechtellidae | | |
|  |  |  |  |  | genus Hubrechtella | |
|  |  |  |  |  |  | Hubrechtella sp. SMOM060 |
|  | order incertae sedis | | | | |  |
|  |  |  |  | family incertae sedis | | |
|  |  |  |  |  | genus insertae sedis | |
|  |  |  |  |  |  | Pilidiophora gen. sp. SMOM093 |
| **class Hoplonemertea** | | | | |  |  |
|  | order Monostilifera | | | | | |
|  |  | suborder Cratenemertea^b^ | | | | |
|  |  |  |  |  | genus Nipponnemertes | |
|  |  |  |  |  |  | Nipponnemertes cf. madagascarensis |
|  |  |  |  |  |  | Nipponnemertes sp. SMOM023 |
|  |  |  |  |  |  | Nipponnemertes sp. SMOM024 |
|  |  |  |  |  |  | Nipponnemertes sp. SMOM084 |
|  |  |  |  |  |  | Nipponnemertes sp. SMOM094 |
|  |  | order Eumonostilifera | | | | |
|  |  |  | infraorder Amphiporina | | | |
|  |  |  |  | family Carcinonemertidae | | |
|  |  |  |  |  | genus Carcinonemertes | |
|  |  |  |  |  |  | Carcinonemertes sp. SMOM106 |
|  |  |  |  | family Ototyphlonemertidae | | |
|  |  |  |  |  | genus Ototyphlonemertes | |
|  |  |  |  |  |  | Ototyphlonemertes sp. SMOM091 |
|  |  |  |  | family insertae sedis** | | |
|  |  |  |  |  | genus Arctostemma | |
|  |  |  |  |  |  | Arctostemma sp. SMOM032 |
|  |  |  |  |  | genus Nemertopsis | |
|  |  |  |  |  |  | Nemertopsis sp. SMOM030 |
|  |  |  |  |  | genus Poseidonemertes | |
|  |  |  |  |  |  | Poseidonemertes sp. SMOM061 |
|  |  |  |  |  |  | Poseidonemertes sp. SMOM062 |
|  |  |  |  |  | genus Prosadenoporus | |
|  |  |  |  |  |  | Prosadenoporus sp. SMOM010 |
|  |  |  |  |  |  | Prosadenoporus sp. SMOM011 |
|  |  |  |  |  | genus Tetrastemma | |
|  |  |  |  |  |  | Tetrastemma sp. SMOM005 |
|  |  |  |  |  |  | Tetrastemma sp. SMOM006 |
|  |  |  |  |  |  | Tetrastemma sp. SMOM007 |
|  |  |  |  |  |  | Tetrastemma sp. SMOM008 |
|  |  |  |  |  |  | Tetrastemma sp. SMOM009 |
|  |  |  |  |  |  | Tetrastemma sp. SMOM012 |
|  |  |  |  |  |  | Tetrastemma sp. SMOM018 |
|  |  |  |  |  |  | Tetrastemma sp. SMOM019 |
|  |  |  |  |  |  | Tetrastemma sp. SMOM020 |
|  |  |  |  |  |  | Tetrastemma sp. SMOM033 |
|  |  |  |  |  |  | Tetrastemma sp. SMOM034 |
|  |  |  |  |  |  | Tetrastemma sp. SMOM063 |
|  |  |  |  |  |  | Tetrastemma sp. SMOM077 |
|  |  |  |  |  |  | Tetrastemma sp. SMOM083 |
|  |  |  |  |  |  | Tetrastemma sp. SMOM095 |
|  |  |  |  |  |  | Tetrastemma sp. SMOM107 |
|  |  |  |  |  | genus Zygonemertes | |
|  |  |  |  |  |  | Zygonemertes sp. SMOM001 |
|  |  |  |  |  |  | Zygonemertes sp. SMOM002 |
|  |  |  |  |  |  | Zygonemertes sp. SMOM003 |
|  |  |  |  |  |  | Zygonemertes sp. SMOM004 |
|  |  |  | infraorder Oerstediina | | | |
|  |  |  |  | family Oerstediidae | | |
|  |  |  |  |  | genus Diplomma | |
|  |  |  |  |  |  | Diplomma cf. albimarginatum |
|  |  |  |  |  |  | Diplomma serpentinum |
|  |  |  |  |  | genus Nemetellina | |
|  |  |  |  |  |  | Nemertellina sp. SMOM021 |
|  |  |  |  |  | genus Oerstedia | |
|  |  |  |  |  |  | Oerstedia sp. SMOM029 |
|  |  |  |  |  | genus Tetranemertes | |
|  |  |  |  |  |  | Tetranemertes arabica |
|  |  |  |  |  |  | Tetranemertes paulayi |
|  |  |  |  |  |  | Tetranemertes cf. rubrolineata^c^ |
|  |  |  |  |  |  | Tetranemertes unistriata |
|  |  |  |  | family insertae sedis | | |
|  |  |  |  |  | genus insertae sedis | |
|  |  |  |  |  |  | Oerstediina gen. sp. SMOM025 |
|  |  |  |  |  |  | Oerstediina gen. sp. SMOM028 |
|  | order Polystilifera | | | | | |
|  |  | suborder Reptantia | | | | |
|  |  |  |  | family Drepanophoridae | | |
|  |  |  |  |  | genus Drepanophorus | |
|  |  |  |  |  |  | Drepanophorus sp. SMOM022 |
|  |  |  |  |  |  | Drepanophorus sp. SMOM088 |

^a^ Non-monophyletic.

^b^ Familial classification currently suspended.

^c^ *Tetranemertes rubrolineata* was described from Madagascar (Kirsteuer, 1965). While species encountered by us in Oman fits the description (Cherneva et al. 2023), we have since discovered several cryptic lineages in the Red Sea (Maslakova et al., unpublished), which makes it doubtful whether the species in Oman is the same as in Madagascar. Collecting and DNA-barcoding specimens from Madagascar is critical to confirm identification of Arabian specimens.
