# Supplementary material for: A hundred species, mostly new—first assessment of ribbon worm diversity and distribution in Oman
Source: PeerJ. 2025 May 28;13:e19438. doi: 10.7717/peerj.19438 (PMC12126093; doi:10.7717/peerj.19438)
Supplement: Supplemental Information 5 — Species marked with an asterisk (*) lack sequence data and are identified based on external morphological characteristics. We use open nomenclature term “cf.” to indicate that the species in Oman resembles a previously described species from another area, but we lack sequence data from the type locality to confirm that these are indeed the same. “X” represents presence in the region. Abbreviations: B, habitat type; (H, hard bottom; S, soft bottom;), C, cryptic species; ON, Northern Oman; OSD, Dhofar (Southern Oman), and OSM, Masirah Island (Southern Oman); S, singleton, D, doubleton. [file peerj-13-19438-s005.docx]

**Supplemental Table 3. Habitat and distribution data for nemertean species and BINs in Oman.** Species marked with an asterisk (*) lack sequence data and are identified based on external morphological characteristics. We use open nomenclature term “cf.” to indicate that the species in Oman resembles a previously described species from another area, but we lack sequence data from the type locality to confirm that these are indeed the same. “X” represents presence in the region. Abbreviations: B – habitat type (H – hard bottom, S – soft bottom), C – cryptic species, ON — Northern Oman, OSD — Dhofar (Southern Oman), and OSM — Masirah Island (Southern Oman), S – singleton, D – doubleton.

| **Species** | **MOTU** | **BIN** | **ON** | **OSD** | **OSM** | **B** | **C** | **S** | **D** | **Note** |
| --- | --- | --- | --- | --- | --- | --- | --- | --- | --- | --- |
| Arctostemma sp. SMOM032 | SMOM032 | AFA8791 | x |  |  | S |  |  |  |  |
| Baseodiscus cf. insignis | SMOM082 | AFJ0636 |  | x |  | H | x |  |  |  |
| Baseodiscus hemprichii | SMOM069 | ADW6007 |  | x |  | H | x | x |  |  |
| Baseodiscus sp. SMOM101 | SMOM101 | AGA5007 |  | x |  | H | x | x |  | hemprichii complex |
| *Carcinonemertes sp. SMOM106 | N/A | N/A | x |  |  | H | x |  |  |  |
| Carinoma sp. SMOM074 | SMOM074 | AFA2453 | x |  |  | S | x |  |  |  |
| Carinoma sp. SMOM075 | SMOM075 | AFA2452 | x |  |  | S | x |  |  |  |
| Carinoma sp. SMOM081 | SMOM081 | AFA2451 | x |  |  | S | x |  |  |  |
| Carinoma sp. SMOM087 | SMOM087 | AFJ0161 | x |  |  | S | x |  |  |  |
| Carinoma sp. SMOM090 | SMOM090 | AFJ0142 |  | x |  | S | x |  |  |  |
| Cephalothrix sp. SMOM035 | SMOM035 | ACQ5911 |  | x |  | H | x |  | x |  |
| Cephalothrix sp. SMOM036 | SMOM036 | AFB0318 | x |  |  | S | x |  | x |  |
| Cephalothrix sp. SMOM064 | SMOM064 | AFB2341 | x |  |  | S | x |  | x |  |
| Cephalotrichella sp. SMOM017 | SMOM017 | AFB5043 | x |  |  | S | x | x |  |  |
| Cephalotrichella sp. SMOM076 | SMOM076 | AFA8889 | x |  |  | S | x |  | x |  |
| Cerebratulus sp. SMOM047 | SMOM047 | AFA8612 |  | x |  | H | x | x |  | krempfi complex |
| Diplomma cf. albimarginatum | SMOM027 | AFB4366 |  | x |  | H | x |  |  |  |
| Diplomma serpentinum | SMOM026 | ACQ1696 | x |  |  | H | x |  |  |  |
| Drepanophorus sp. SMOM022 | SMOM022 | AFA3451 | x | x |  | H | x |  |  |  |
| Drepanophorus sp. SMOM088 | SMOM088 | AFJ0209 |  |  | x | H | x | x |  |  |
| Dushia sp. SMOM070 | SMOM070 | AFA8611 | x |  |  | H | x | x |  |  |
| Dushia sp. SMOM071 | SMOM071 | AFB4780 | x |  |  | H | x |  |  |  |
| Eopilidiidae gen. sp. SMOM048 | SMOM048 | AFA6563 | x |  |  | S |  | x |  |  |
| Eousia sp. SMOM051 | SMOM051 | AFB1082 |  | x |  | H | x |  | x | verticivaria complex |
| Eousia sp. SMOM052 | SMOM052 | AFA8613 | x |  |  | H | x | x |  | verticivaria complex |
| Euborlasia sp. SMOM043 | SMOM043 | AFB3465 |  | x | x | H | x |  |  |  |
| Gorgonorhynchus sp. SMOM045 | SMOM045 | AFB2325 | x |  | x | H | x |  |  | repens complex |
| Gorgonorhynchus sp. SMOM050 | SMOM050 | AFA3894 |  | x |  | H | x | x |  |  |
| Gorgonorhynchus sp. SMOM102 | SMOM102 | AFI9417 |  | x |  | H | x |  | x | repens complex |
| Heteronemertea gen. sp. SMOM079 | SMOM079 | AFJ0555 | x |  |  | S |  | x |  |  |
| Hubrechtella sp. SMOM060 | SMOM060 | AFB1359 | x |  |  | S | x |  |  |  |
| *Lineidae gen. sp. SMOM104 | N/A | N/A | x |  |  | S | x | x |  |  |
| Lineidae gen. sp. SMOM044 | SMOM044 | AFA3223 | x |  |  | S |  |  | x |  |
| Lineidae gen. sp. SMOM046 | SMOM046 | AFA3224 | x | x | x | H |  |  |  |  |
| Lineidae gen. sp. SMOM049 | SMOM049 | AFA4480 | x |  |  | S |  | x |  |  |
| Lineidae gen. sp. SMOM053 | SMOM053 | AFB1286 | x |  |  | S | x | x |  |  |
| Lineidae gen. sp. SMOM058 | SMOM058 | AFA4481 |  | x |  | H | x |  | x |  |
| Lineidae gen. sp. SMOM065 | SMOM065 | AFB1287 | x |  |  | S | x | x |  |  |
| Lineidae gen. sp. SMOM066 | SMOM066 | AFA8610 | x |  |  | S | x |  |  |  |
| Lineidae gen. sp. SMOM067 | SMOM067 | AFB0485 |  | x |  | H | x |  |  |  |
| Lineidae gen. sp. SMOM068 | SMOM068 | AFB1288 | x |  |  | H |  | x |  |  |
| Lineidae gen. sp. SMOM073 | SMOM073 | AFA3222 |  | x |  | H | x | x |  |  |
| Lineidae gen. sp. SMOM080 | SMOM080 | AFB0699 | x |  |  | S | x | x |  |  |
| Lineidae gen. sp. SMOM085 | SMOM085 | AFJ0539 |  | x |  | H | x |  |  |  |
| Lineidae gen. sp. SMOM086 | SMOM086 | AFJ0537 | x |  |  | S | x | x |  |  |
| Lineidae gen. sp. SMOM089 | SMOM089 | AFJ0554 |  | x |  | H | x | x |  |  |
| Lineidae gen. sp. SMOM092 | SMOM092 | AFJ0613 |  | x |  | H | x |  | x |  |
| Lineidae gen. sp. SMOM092 | SMOM092 | AFX7905 |  | x |  | H | x |  | x |  |
| Lineidae gen. sp. SMOM096 | SMOM096 | AFJ0538 | x |  |  | S |  | x |  |  |
| Lineidae gen. sp. SMOM097 | SMOM097 | AFI9736 |  | x |  | S |  | x |  |  |
| Lineidae gen. sp. SMOM098 | SMOM098 | AFX7906 | x |  |  | S | x | x |  |  |
| Lineidae gen. sp. SMOM099 | SMOM099 | AFX7908 |  | x |  | H | x |  | x |  |
| Lineidae gen. sp. SMOM100 | SMOM100 | AFX9707 | x |  |  | S | x | x |  |  |
| Bilucernus caputornatus | SMOM054 | ACA9932 |  | x | x | H |  |  |  |  |
| *Micrura sp. SMOM103 | N/A | N/A |  | x |  | H |  |  |  |  |
| Nemertellina sp. SMOM021 | SMOM021 | AFB1635 |  | x |  | H | x |  |  |  |
| Nemertopsis sp. SMOM030 | SMOM030 | AFA8144 | x |  |  | H | x | x |  | bivittata complex |
| Nipponnemertes cf. madagascarensis | SMOM031 | AFA2388 |  | x |  | H | x | x |  |  |
| Nipponnemertes sp. SMOM023 | SMOM023 | AFA2387 |  | x |  | H | x |  |  |  |
| Nipponnemertes sp. SMOM024 | SMOM024 | AFB3551 |  | x |  | H | x | x |  |  |
| Nipponnemertes sp. SMOM084 | SMOM084 | AFJ0458 |  | x |  | H | x | x |  |  |
| Nipponnemertes sp. SMOM094 | SMOM094 | AFJ0664 |  |  | x | H | x | x |  |  |
| Notospermus sp. SMOM055 | SMOM055 | AFB1537 | x | x |  | H | x |  |  | tricuspidatus complex |
| Notospermus sp. SMOM056 | SMOM056 | AFB1538 |  | x |  | H | x |  | x |  |
| Notospermus sp. SMOM057 | SMOM057 | AFA6759 |  | x |  | H | x |  |  |  |
| Oerstedia sp. SMOM029 | SMOM029 | AFA5574 |  | x |  | H | x |  |  |  |
| Oerstediina gen. sp. SMOM025 | SMOM025 | NA |  | x |  | H | x |  |  |  |
| Oerstediina gen. sp. SMOM025 | SMOM025 | AFA9738 | x |  |  | H | x |  |  |  |
| Oerstediina gen. sp. SMOM028 | SMOM028 | AFA7799 |  | x |  | H | x | x |  |  |
| Ototyphlonemertes sp. SMOM091 | SMOM091 | AFI9497 |  | x |  | S | x | x |  |  |
| Oxypolella sp. SMOM072 | SMOM072 | AFB4858 |  | x |  | H | x |  | x |  |
| Pilidiophora gen. sp. SMOM093 | SMOM093 | AFI9777 |  | x |  | S | x | x |  |  |
| *Polydendrorhynchus sp. SMOM104 | N/A | N/A |  |  | x | S | x | x |  |  |
| Poseidonemertes sp. SMOM061 | SMOM061 | AFA8707 | x |  |  | S | x | x |  |  |
| Poseidonemertes sp. SMOM062 | SMOM062 | AFA8706 | x |  |  | S | x | x |  |  |
| Prosadenoporus sp. SMOM010 | SMOM010 | AFB0431 | x |  |  | H | x | x |  |  |
| Prosadenoporus sp. SMOM011 | SMOM011 | AFB0430 | x |  |  | H | x | x |  |  |
| Siphonenteron sp. SMOM059 | SMOM059 | AFA4873 | x |  |  | H | x |  |  |  |
| Siphonenteron sp. SMOM059 | SMOM059 | AFA4874 |  | x |  | H | x |  |  |  |
| Tetranemertes arabica | SMOM014 | AFA7129 |  | x |  | H | x |  |  |  |
| Tetranemertes paulayi | SMOM016 | AFA4223 |  | x |  | H | x |  |  |  |
| Tetranemertes cf. rubrolineata | SMOM015 | AFA7128 |  | x |  | H | x |  |  |  |
| Tetranemertes unistriata | SMOM013 | AFA7130 |  | x |  | H | x | x |  |  |
| *Tetrastemma sp. SMOM107 | N/A | N/A |  | x |  | H |  | x |  |  |
| Tetrastemma sp. SMOM005 | SMOM005 | AFB4583 | x | x |  | H | x |  |  |  |
| Tetrastemma sp. SMOM006 | SMOM006 | AFB4584 |  | x |  | H | x |  |  |  |
| Tetrastemma sp. SMOM007 | SMOM007 | AFB4585 | x | x | x | H | x |  |  |  |
| Tetrastemma sp. SMOM008 | SMOM008 | AFA7771 | x |  |  | H | x |  |  |  |
| Tetrastemma sp. SMOM008 | SMOM008 | AFA7772 |  | x |  | H | x |  |  |  |
| Tetrastemma sp. SMOM008 | SMOM008 | AFJ0140 |  |  | x | H | x |  |  |  |
| Tetrastemma sp. SMOM009 | SMOM009 | AFA7768 |  | x |  | H | x | x |  |  |
| Tetrastemma sp. SMOM012 | SMOM012 | AFA7767 |  | x |  | H |  | x |  |  |
| Tetrastemma sp. SMOM018 | SMOM018 | AFA7770 |  | x |  | H | x | x |  |  |
| Tetrastemma sp. SMOM019 | SMOM019 | AFA7764 | x |  |  | H | x |  |  |  |
| Tetrastemma sp. SMOM019 | SMOM019 | AFA7769 |  | x | x | H | x |  |  |  |
| Tetrastemma sp. SMOM020 | SMOM020 | AFA7765 | x | x | x | H | x |  |  |  |
| Tetrastemma sp. SMOM020 | SMOM020 | AFA7763 | x |  |  | H | x |  |  |  |
| Tetrastemma sp. SMOM020 | SMOM020 | AFA7766 | x |  |  | H | x |  |  |  |
| Tetrastemma sp. SMOM033 | SMOM033 | AFB3967 | x |  |  | S | x | x |  |  |
| Tetrastemma sp. SMOM034 | SMOM034 | AFB3968 | x |  |  | H | x | x |  |  |
| Tetrastemma sp. SMOM063 | SMOM063 | AFB3965 |  | x |  | H | x | x |  |  |
| Tetrastemma sp. SMOM077 | SMOM077 | AFB3966 | x |  |  | H | x | x |  |  |
| Tetrastemma sp. SMOM083 | SMOM083 | AFI9508 | x |  |  | S |  | x |  |  |
| Tetrastemma sp. SMOM095 | SMOM095 | AFJ0230 | x |  |  | S |  | x |  |  |
| Tubulanus cf. aureus | SMOM038 | AFA2649 |  | x |  | H | x | x |  |  |
| Tubulanus sp. SMOM037 | SMOM037 | AFA6233 |  | x |  | H | x |  | x |  |
| Tubulanus sp. SMOM039 | SMOM039 | AFB2986 | x |  |  | S | x | x |  |  |
| Tubulanus sp. SMOM040 | SMOM040 | AFB4484 |  | x |  | H | x | x |  |  |
| Tubulanus sp. SMOM041 | SMOM041 | AFA6213 | x |  |  | S | x | x |  |  |
| Tubulanus sp. SMOM042 | SMOM042 | AFB3846 | x |  |  | S | x | x |  |  |
| Tubulanus sp. SMOM078 | SMOM078 | N/A | x |  |  | S | x | x |  |  |
| Zygonemertes sp. SMOM001 | SMOM001 | AFB1511 | x |  |  | H | x | x |  |  |
| Zygonemertes sp. SMOM002 | SMOM002 | AEI8509 | x | x |  | H | x |  |  |  |
| Zygonemertes sp. SMOM003 | SMOM003 | AFB1509 | x | x | x | H | x |  |  |  |
| Zygonemertes sp. SMOM004 | SMOM004 | AFB1510 |  | x | x | H | x |  |  |  |
