# Supplementary material for: A hundred species, mostly new—first assessment of ribbon worm diversity and distribution in Oman
Source: PeerJ. 2025 May 28;13:e19438. doi: 10.7717/peerj.19438 (PMC12126093; doi:10.7717/peerj.19438)
Supplement: Supplemental Information 6 — Representative nemertean sequences (1 per BIN) from Oman, and those of other available genetically characterized nemertean taxa (BOLD BINs), plus additional sequences from GenBank, and our unpublished sequences from the Caribbean, Panama Bight, Red Sea, Guam, and Moorea. Unpublished sequences can be made available from the corresponding author upon a reasonable request. [file peerj-13-19438-s006.docx]

**Supplemental Table 4. A list of nemertean reference sequences used for phylogenetic context of Oman data.** Representative nemertean sequences (1 per BIN) from Oman, and those of other available genetically characterized nemertean taxa (BOLD BINs), plus additional sequences from GenBank, and our unpublished sequences from the Caribbean, Panama Bight, Red Sea, Guam, and Moorea. Unpublished sequences can be made available from the corresponding author upon a reasonable request.

| **Taxon** | **GenBank Accession** | **BOLD Process ID** | **BOLD BIN** | **Specimen Number** |
| --- | --- | --- | --- | --- |
| Abyssonemertes kajiharai | KY296906 | GBSP14438-19 |  |  |
| Acteonemertes sp. | OQ466705 |  |  |  |
| Acteonemertidae sp. | EU255604 | GBSP5466-13 | BOLD:ACH2954 |  |
| Acteonemertidae sp. | EU255621 | GBSP5449-13 | BOLD:ACH2953 |  |
| Acteonemertidae sp. | EU255628 | GBSP5442-13 | BOLD:ACH3146 |  |
| Alvinonemertes christianeae | ON021867 |  |  |  |
| Alvinonemertes claudiae | ON021868 |  |  |  |
| Alvinonemertes dagmarae | ON021866 |  |  |  |
| Alvinonemertes dariae | ON021865 |  |  |  |
| Alvinonemertes tatjanae | ON021864 |  |  |  |
| Amphiporidae sp. | KP203859 | GBSP14448-19 | BOLD:ADW6608 |  |
| Amphiporidae sp. | KY296907 | GBSP14458-19 | BOLD:ADW6607 |  |
| Amphiporidae sp. | KY296913 | GBSP14650-19 | BOLD:ADW6606 |  |
| Amphiporidae sp. | LC505451 | GBMNC35090-20 | BOLD:AEG0135 |  |
| Amphiporidae sp. | MG421992 | NNOP054-08 | BOLD:AAP2669 | HLC-30034 |
| Amphiporidae sp. | MG422838 | NNOP059-08 | BOLD:AAP2671 | HLC-30179 |
| Amphiporidae sp. | MN205522 | GBMNC35156-20 | BOLD:AEG0136 |  |
| Amphiporina sp. CB140 | OK081457 | CARNE377-21 | BOLD:AEI3274 | Hoplo_CB140_CB141_18_08 |
| Amphiporina sp. CB151 | OK081532 | CARNE403-21 | BOLD:AEI8511 | Hoplo_CB151_CB151_18_01 |
| Amphiporina sp. CB171 | OK081343 | CARNE439-21 | BOLD:AEI8510 | Hoplo_CB171_CB141_18_11 |
| Amphiporus aff. cruentatus |  | NECOL037-23 | BOLD:AFN3678 | Co_Mon_005 |
| Amphiporus allucens | KC812591 | GBSP7825-13 | BOLD:AAK1237 |  |
| Amphiporus angulatus | MG421582 | NNOP068-08 | BOLD:AAN6733 | HLC-30533 |
| Amphiporus angulatus | MG421935 | ARCMI352-14 | BOLD:AAG3609 | BIOUG14667-F09 |
| Amphiporus cruentatus | OQ075659 | NONEP045-21 | BOLD:AEI5687 | BON_61_2020 |
| Amphiporus formidabilis | KC710979 | GBMNA12762-19 | BOLD:AAK1236 |  |
| Amphiporus imparispinosus | OK081432 | POIMB597-19 | BOLD:AEA1921 | BOIMB_0475 |
| Amphiporus imparispinosus |  | BBPS335-19 | BOLD:AEB2317 | 3497_DNA |
| Amphiporus imparispinosus |  | BHAK2519-20 | BOLD:AAK1235 |  |
| Amphiporus lactifloreus | MZ558351 | GBMNE6985-21 | BOLD:AEN1869 |  |
| Amphiporus lactifloreus |  | MBAM211-24 | BOLD:AAK1244 | MBA-230422-003A |
| Amphiporus sp. | EU255601 | GBSP5469-13 | BOLD:ACH2424 |  |
| Amphiporus sp. | KC812590 | GBSP7826-13 | BOLD:ACI0283 |  |
| Amphiporus sp. | MG421941 | CCSMA188-10 | BOLD:AAY6348 | 08PROBE-1136 |
| Amphiporus sp. | MG421942 | NNOP066-08 | BOLD:ABZ4969 | HLC-30479 |
| Amphiporus sp. | MN205525 | GBMNC35153-20 | BOLD:AEG6978 |  |
| Amphiporus sp. | OQ322763 |  |  |  |
| Amphiporus sp. | OQ322917 |  | BOLD:ACH2424 |  |
| Amphiporus sp. | OQ626346 |  |  |  |
| Amphiporus sp. |  | DISA999-19 | BOLD:ADR7156 | LACM:DISCO:7708 |
| Amphiporus sp. |  | KBINV022-11 | BOLD:ABY8223 | 11BIOAK-1568 |
| Amphiporus sp. BOBA017 | OQ075661 | NONEP040-21 | BOLD:ADR7530 | BON_47_2020 |
| Amphiporus sp. BOBA018 | OQ075660 | NONEP029-21 | BOLD:AEA1922 | BON_16_2020 |
| Amphiporus sp. CB058 | OK081392 | CARNE320-21 | BOLD:AEA9103 | Hoplo_CB058_CB058_18_01 |
| Amphiporus sp. ETP005 | OK081666 | NOPP007-21 | BOLD:AEK3307 | ETP005_SMPP0791 |
| Amphiporus sp. ETP043 | OK081434 | NOPP067-21 | BOLD:AEL2802 | ETP043_SMPP0702 |
| Amphiporus sp. OR027 | KU197588 | ORNE070-21 | BOLD:AEA0204 | Hoplo_OR027_E2H4 |
| Amphiporus sp. SMGM034 |  | NEGAM070-24 | BOLD:AGE6203 | GLTZ-0411 |
| Antarctonemertes delutebasisae | AJ436900 | GBSP0355-06 | BOLD:AAW5987 |  |
| Antarctonemertes papilliformis | MZ216515 | GBMNF18510-22 | BOLD:AEU5023 |  |
| Antarctonemertes phyllospadicola | OQ075664 | NONEP050-21 | BOLD:ACH3602 | BON_67_2020 |
| Antarctonemertes riesgoae |  | INTGS095-22 | BOLD:ADX3908 | NEM_H_0330.1 |
| Antarctonemertes sp. | MZ216516 | GBMNF18511-22 | BOLD:AET7512 |  |
| Antarctonemertes sp. | OM456697 | GBMNF77459-22 | BOLD:AEZ4154 |  |
| Antarctonemertes unilineata | MG948830 | GBSP14864-19 | BOLD:ADW5513 |  |
| Antarctonemertes valida | MT414869 | GBMNC35259-20 | BOLD:AEG0339 |  |
| Antarctonemertes valida |  | INTGS088-22 | BOLD:ADW7916 | NEM_A_0078 |
| Apatronemertes albimaculosa | HQ848584 |  | BOLD:ADL9805 |  |
| Arctostemma arcticum | MZ216538 | GBMNF18521-22 | BOLD:AES2537 |  |
| Arctostemma sp. SMOM032 | PP834669 | NOMAN112-23 |  | BOMAN-11552 |
| Arenogigas armoricus | KP119170 | GBMIN138739-18 | BOLD:ADM2366 |  |
| Argonemertes australiensis | AY428840 |  | BOLD:ADX8044 |  |
| Arhochmus korotneffi | EF157592 | GBSP5823-13 | BOLD:ACH2645 |  |
| Balionemertes australiensis |  | NERS013-23 | BOLD:AEA8716 | BDJRS_4919 |
| Balionemertes sp. | KP270866 | GBSP10425-18 | BOLD:ADM3326 |  |
| Balionemertes sp. SMGM001 |  | NEGAM001-24 | BOLD:ADM3326 | GLTZ-0241 |
| Baseodiscus aff. rubrolineata |  | NERS005-23 | BOLD:AFI9889 | BDJRS_3260 |
| Baseodiscus aureus | KC812601 | GBSP7816-13 | BOLD:ACH5862 |  |
| Baseodiscus cf. insignis | N/A | NERS006-23 | BOLD:AFJ0636 | BDJRS_3333 |
| Baseodiscus cf. insignis | PP834677 | NOMAN201-23 | BOLD:AFJ0636 | BOMAN-7036 |
| Baseodiscus delineatus | MK047687 | GBSP17187-19 | BOLD:ADW6006 |  |
| Baseodiscus delineatus sp. | N/A | N/A | N/A | BMOO_08239 |
| Baseodiscus hemprichii | N/A | NEGAM021-24 | BOLD:ADW6007 | GLTZ-0387 |
| Baseodiscus hemprichii | PP834672 | NOMAN174-23 | BOLD:ADW6007 | BOMAN-8268 |
| Baseodiscus komatsui | N/A | NEGAM003-24 | BOLD:AES5992 | GLTZ-0246 |
| Baseodiscus maculosus | LC190950 | GBMNF18591-22 | BOLD:AET9630 |  |
| Baseodiscus mexicanus | OK081730 | NOPP036-21 | BOLD:ADW9207 | ETP016_B1_69 |
| Baseodiscus paracelensis | LC190952 | GBMNF18590-22 | BOLD:AET9629 |  |
| Baseodiscus punnetti | LC190954 | GBMNF18593-22 | BOLD:ADX6894 |  |
| Baseodiscus quinquelineatus | LC190955 | GBMNF18594-22 | BOLD:AEA8444 |  |
| Baseodiscus sp. | EF124962 | GBSP3694-12 | BOLD:ACB0153 |  |
| Baseodiscus sp. | HQ848588 |  |  |  |
| Baseodiscus sp. | HQ848589 | GBSP17882-21 | BOLD:AEM0971 |  |
| Baseodiscus sp. | LC190942 | GBMNF18597-22 | BOLD:AES5995 |  |
| Baseodiscus sp. | LC190944 | GBMNF18586-22 | BOLD:AEU0923 |  |
| Baseodiscus sp. | LC190945 | GBMNF18598-22 | BOLD:AES5991 |  |
| Baseodiscus sp. | LC190949 | GBMNF18599-22 | BOLD:AES5992 |  |
| Baseodiscus sp. | LC190951 | GBMNF18600-22 | BOLD:AES5993 |  |
| Baseodiscus sp. | LC190956 | GBMNF18601-22 | BOLD:AES5994 |  |
| Baseodiscus sp. | LC190959 | GBMNF18604-22 | BOLD:AES5988 |  |
| Baseodiscus sp. | LC190960 | GBMNF18605-22 | BOLD:AES5989 |  |
| Baseodiscus sp. |  | DISA902-19 |  | LACM:DISCO:7133 |
| Baseodiscus sp. CB004 | MW021772 | CARNE082-19 | BOLD:AEA9219 | Pili_CB004_BdT030_1 |
| Baseodiscus sp. CB0136 | OK081461 | CARNE359-21 | BOLD:AEI3875 | Pili_CB135_CB135_18_01 |
| Baseodiscus sp. ETP017 | N/A | NOPP198-23 | BOLD:AFR1428 | Pili_ETP017_SMPP0895 |
| Baseodiscus sp. ETP018 | N/A | NOPP200-23 | BOLD:ADL0021 | Pili_ETP018_SMPP0859 |
| Baseodiscus sp. OR100 | OK081627 | ORNE406-21 | BOLD:AEL5181 | Pili_OR100_BOIMB_2598 |
| Baseodiscus sp. SMOM101 | PP834671 | NOMAN285-24 | BOLD:AGA5007 | BOMAN-6733 |
| Baseodiscus sp. SMRS015 | N/A | NERS015-23 | BOLD:AFI9679 | BDJRS_5752 |
| Baseodiscus takakurai | LC190937 | GBMNF18606-22 | BOLD:AES5990 |  |
| Baseodiscus unicolor | KF935505 | GBSP11444-19 | BOLD:ADW9254 |  |
| Bilucernus caputornatus | PP834817 | NOMAN217-23 | BOLD:ACA9932 | BOMAN-9091 |
| Bilucernus caputornatus | N/A | NEGAM025-24 | BOLD:ACA9932 | GLTZ-2468 |
| Callinera grandis | EU489491 |  |  |  |
| Callinera sp. | KP270864 | GBSP11424-19 | BOLD:ADX6180 |  |
| Carcinonemertes carcinophila | HQ848619 |  |  |  |
| Carcinonemertes conanobrieni | KY741564 |  | BOLD:AEG8400 |  |
| Carcinonemertes epialti | KU197594 | ORNE092-21 | BOLD:AEK9648 | Hoplo_OR036_E4D6 |
| Carcinonemertes sp. | AJ436901 | GBSP0356-06 | BOLD:AAW4327 |  |
| Carcinonemertes sp. | KU197595 | GBSP14649-19 | BOLD:ADW4302 |  |
| Carcinonemertes sp. | MH753556 | GBSP14891-19 |  |  |
| Carcinonemertes sp. | MT872631 | GBMNC35099-20 | BOLD:AEG6275 |  |
| Carcinonemertes sp. | MW596481 | GBMNF18459-22 | BOLD:AES2349 |  |
| Carcinonemertes sp. | MW596485 | GBMNF18461-22 | BOLD:AES2350 |  |
| Carinina chocolata | KU197655 | ORNE149-21 | BOLD:AEL5217 | Palaeo_OR050_3 |
| Carinina chocolata | KU197657 | ORNE153-21 | BOLD:AEL5216 | Palaeo_OR050_E1E1 |
| Carinina chocolata | KU197660 | ORNE157-21 | BOLD:AEL0127 | Palaeo_OR051_E3C5 |
| Carinina ochracea | KM487742 | GBSP10434-18 | BOLD:AAM4521 |  |
| Carinina plecta | EU489493 |  |  |  |
| Carinina sp. | KP270863 | GBSP10435-18 | BOLD:ADM6708 |  |
| Carininidae sp. | MN205489 | GBMNC35189-20 |  |  |
| Carininidae sp. | MN205491 | GBMNC35187-20 | BOLD:AEG4780 |  |
| Carininidae sp. | MN205493 | GBMNC35185-20 | BOLD:AEG4779 |  |
| Carinoma mutabilis | KU197669 | ORNE130-21 | BOLD:AEL5779 | Palaeo_OR039_E2D7 |
| Carinoma mutabilis | OK081408 | POIMB360-19 | BOLD:AAY6351 | BOIMB_0457 |
| Carinoma sp. | KF935500 | GBSP11440-19 | BOLD:ADW2331 |  |
| Carinoma sp. | KJ592725 | SDP100034-13 | BOLD:ACM2397 | F12LK05 |
| Carinoma sp. | MG421956 | CCSMA184-10 | BOLD:AAG3608 | 07PROBE-02896 |
| Carinoma sp. | MH235833 |  |  |  |
| Carinoma sp. |  | BBPS809-19 | BOLD:ADX5108 |  |
| Carinoma sp. |  | CMBIA533-12 | BOLD:ACH0320 | MBI-SCCWRP-00469 |
| Carinoma sp. ETP053 | OK081774 | NOPP076-21 | BOLD:AEK2772 | ETP053_SMPP0740 |
| Carinoma sp. OR037 | KU197673 | ORNE137-21 | BOLD:AEL3040 | Palaeo_OR042_E2B4 |
| Carinoma sp. OR043 | KU197674 | ORNE138-21 | BOLD:AEK4464 | Palaeo_OR043_34 |
| Carinoma sp. SMOM074 | PP834680 | NOMAN185-23 | BOLD:AFA2453 | BOMAN-10499 |
| Carinoma sp. SMOM075 | PP834685 | NOMAN188-23 | BOLD:AFA2452 | BOMAN-11549 |
| Carinoma sp. SMOM081 | PP834692 | NOMAN231-23 | BOLD:AFA2451 | BOMAN-11542 |
| Carinoma sp. SMOM087 | PP834696 | NOMAN226-23 | BOLD:AFJ0161 | BOMAN-11504 |
| Carinoma sp. SMOM090 | PP834699 | NOMAN275-23 | BOLD:AFJ0142 | BOMAN-16115 |
| Carinoma tremaphoros | AJ436943 | GBSP0398-06 | BOLD:AAW4271 |  |
| Carinoma tremaphoros | HQ848630 |  |  |  |
| Carinoma tremaphoros | OQ322653 |  | BOLD:AAW4271 |  |
| Cephalothrichella sp. CB141 | OK081429 | CARNE382-21 | BOLD:AEI5099 | Palaeo_CB141_MCB004_18_01 |
| Cephalothrichella sp. CB150 | OK081368 | CARNE252-21 | BOLD:ACQ0981 | Palaeo_CB034_MCB004_18_05 |
| Cephalothrichella sp. CB150 | OK081615 | CARNE402-21 | BOLD:ADM6001 | Palaeo_CB150_MCB004_18_03 |
| Cephalothrichella sp. ETP011 | OK081687 | NOPP009-21 | BOLD:AEK7639 | ETP011_SMPP0786 |
| Cephalothricidae sp. | N/A | N/A | N/A | BMOO_07924 |
| Cephalothrix alba | KM083819 | GBMAA674-14 | BOLD:ACQ1369 |  |
| Cephalothrix bipunctata | KF935501 | GBSP11459-19 | BOLD:ADX1903 |  |
| Cephalothrix cf. | GU726643 | GBMAA703-14 | BOLD:AAK0877 |  |
| Cephalothrix fasciculus | GU726623 | GBMAA611-14 | BOLD:ACQ5910 |  |
| Cephalothrix filiformis | GU726645 | GBMAA610-14 | BOLD:AAX8105 |  |
| Cephalothrix hermaphroditicus | OQ075669 | NONEP036-21 | BOLD:ADM3467 | BON_38_2020 |
| Cephalothrix hongkongiensis |  | GBMTG2068-16 | BOLD:ACQ2010 | NC_012821 |
| Cephalothrix iwatai | KP270873 | GBSP10430-18 | BOLD:ADM5301 |  |
| Cephalothrix linearis | LC422243 | GBSP16398-19 | BOLD:AEB6216 |  |
| Cephalothrix major | GU726689 | GBMAA910-14 |  |  |
| Cephalothrix oestrymnica | MH681908 | GBSP17070-19 | BOLD:AEB6328 |  |
| Cephalothrix rufifrons | EF140788 | GBSP10203-13 | BOLD:AAB1713 | EF140788 |
| Cephalothrix rufifrons | MK047675 | GBSP17175-19 | BOLD:ACQ0932 |  |
| Cephalothrix simula | OQ075672 | NONEP042-21 | BOLD:AAM5519 | BON_51_2020 |
| Cephalothrix sp. | GU564482 | GBMNA12772-19 | BOLD:ACD2623 |  |
| Cephalothrix sp. | GU726616 | GBMAA750-14 | BOLD:ACQ0933 |  |
| Cephalothrix sp. | GU726621 | GBMAA920-14 | BOLD:ACQ0547 |  |
| Cephalothrix sp. | GU726631 | GBMAA385-14 | BOLD:ACQ0936 |  |
| Cephalothrix sp. | GU726666 | GBMAA624-14 | BOLD:ACQ0545 |  |
| Cephalothrix sp. | GU726667 | GBMAA623-14 | BOLD:ACQ0549 |  |
| Cephalothrix sp. | GU726672 | GBMAA493-14 | BOLD:ACQ0935 |  |
| Cephalothrix sp. | GU726681 | GBMAA675-14 | BOLD:ACQ0548 |  |
| Cephalothrix sp. | HQ848602 |  |  |  |
| Cephalothrix sp. | KM083810 | GBMIN44212-15 | BOLD:ACQ0934 | KM083810 |
| Cephalothrix sp. | KM083811 | GBMAA1167-14 | BOLD:ACQ0541 |  |
| Cephalothrix sp. | KM083812 | GBMAA1170-14 | BOLD:ACQ0544 |  |
| Cephalothrix sp. | KM083813 | GBMAA211-14 | BOLD:ACQ0540 |  |
| Cephalothrix sp. | KM083815 | GBMAA212-14 | BOLD:ACQ0546 |  |
| Cephalothrix sp. | KM083816 | GBMIN44213-15 |  |  |
| Cephalothrix sp. | KM083820 | GBMAA1168-14 | BOLD:ACQ0542 |  |
| Cephalothrix sp. | KP254258 | FLFIV122-14 | BOLD:ACQ2506 |  |
| Cephalothrix sp. | MG421193 | CCANN215-08 | BOLD:AAG3611 | 08PROBE-0126 |
| Cephalothrix sp. | MT999901 | HIMF034-20 | BOLD:AEE5708 | USNM1616802 |
| Cephalothrix sp. | MT999909 | HIMF057-20 | BOLD:ACQ0543 | USNM1616764 |
| Cephalothrix sp. | MT999911 | HIMF052-20 | BOLD:AEE0601 | USNM1616768 |
| Cephalothrix sp. | MW118025 | GBMND99291-21 | BOLD:AEK9765 |  |
| Cephalothrix sp. | MW118026 | GBMND99292-21 | BOLD:AEL1001 |  |
| Cephalothrix sp. | OK081406 | ABBAI101-15 | BOLD:AEM3579 | CCLV052 |
| Cephalothrix sp. |  | DISCT005-17 | BOLD:ACQ0931 | LACM:DISCO:3521 |
| Cephalothrix sp. |  | QHAK876-21 | BOLD:AAX8106 | QHAK-00713 |
| Cephalothrix sp. CB006 | OK081360 | CARNE220-21 | BOLD:ACQ0934 | Palaeo_CB006_MCB017_18_02 |
| Cephalothrix sp. CB154 | OK081720 | CARNE411-21 | BOLD:AEH9309 | Palaeo_CB154_MCB005_18_03 |
| Cephalothrix sp. CB158 | OK081746 | CARNE418-21 | BOLD:AEI5100 | Palaeo_CB158_MCB005_18_01 |
| Cephalothrix sp. CB172 | OK081542 | CARNE440-21 | BOLD:AEH9308 | Palaeo_CB172_MCB005_18_04 |
| Cephalothrix sp. ETP012 | OK081418 | NOPP010-21 | BOLD:AEL3046 | ETP012_SMPP0625 |
| Cephalothrix sp. giglio | MH681955 | GBSP17117-19 | BOLD:AEB1514 |  |
| Cephalothrix sp. giglio | MH681956 | GBSP17118-19 | BOLD:AEB1515 |  |
| Cephalothrix sp. OR022 | KU197692 | ORNE110-21 | BOLD:AEK8142 | Palaeo_OR022_E3C9 |
| Cephalothrix sp. OR034 | KU197683 | ORNE122-21 | BOLD:AEL0140 | Palaeo_OR034_E2D8 |
| Cephalothrix sp. OR037 | KU197695 | ORNE125-21 | BOLD:AEK6232 | Palaeo_OR037_E5A4 |
| Cephalothrix sp. OR097 | OK081751 | ORNE170-21 | BOLD:AEK8143 | Palaeo_OR097_E3A7 |
| Cephalothrix sp. SMGM023 | N/A | NEGAM074-24 | BOLD:AGE4051 | GLTZ-0417 |
| Cephalothrix sp. SMOM035 | PP834701 | NOMAN116-23 | BOLD:ACQ5911 | BOMAN-9049 |
| Cephalothrix sp. SMOM036 | PP834703 | NOMAN118-23 | BOLD:AFB0318 | BOMAN-12282 |
| Cephalothrix sp. SMOM064 | PP834706 | NOMAN167-23 | BOLD:AFB2341 | BOMAN-11508 |
| Cephalothrix spiralis | MK047676 | GBSP17176-19 | BOLD:ACQ6349 |  |
| Cephalotrichella echinicola | MK307889 | GBSP17346-19 | BOLD:AEB2689 |  |
| Cephalotrichella sp. SMOM017 | PP834707 | NOMAN053-23 | BOLD:AFB5043 | BOMAN-2844 |
| Cephalotrichella sp. SMOM076 | PP834709 | NOMAN190-23 | BOLD:AFA8889 | BOMAN-11507 |
| Cephalotrichella sp. SMRS002 | N/A | NERS085-23 | BOLD:AFI9556 | BNOM_2223 |
| Cephalotrichidae sp. | KU839751 |  |  |  |
| Cephalotrichidae sp. | KU839752 |  |  |  |
| Cephalotrichidae sp. | KU839753 |  |  |  |
| Cerebratulus albifrons | KU197715 | ORNE392-21 | BOLD:AEL1050 | Pili_OR093_LWE2 |
| Cerebratulus albifrons | KU197843 | ORNE235-21 | BOLD:ADW4574 | Pili_OR070_146 |
| Cerebratulus californiensis | KU197725 | ORNE212-21 | BOLD:ADX2958 | Pili_OR062_E3F7 |
| Cerebratulus cf. bengalensis | KY991481 | GBSP10918-19 | BOLD:ADW2324 |  |
| Cerebratulus cf. krempfi | PP834710 | NOMAN139-23 | BOLD:AFA8612 | BOMAN-7037 |
| Cerebratulus cf. superinger | LC625634 | GBMNF18610-22 | BOLD:AET6369 |  |
| Cerebratulus formosus | LC625639 | GBMNF18612-22 | BOLD:AET6370 |  |
| Cerebratulus fuscus |  | DTNHM4936-22 |  | NHMUK014451822 |
| Cerebratulus herculeus | EF124991 | GBSP3713-12 | BOLD:AAP2667 |  |
| Cerebratulus lacteus | EF124965 | GBSP14167-19 | BOLD:ACI1190 |  |
| Cerebratulus lacteus | KC424754 |  | BOLD:ACI1190 |  |
| Cerebratulus lacteus | KX261791 | GBSP11400-19 | BOLD:ADW8560 |  |
| Cerebratulus lineolatus | MK047689 | GBSP17189-19 | BOLD:AEB2637 |  |
| Cerebratulus mordukhovichi | OM456681 | GBMNF77443-22 | BOLD:AEZ1955 |  |
| Cerebratulus orochi | LC538102 | GBMNC35114-20 | BOLD:AEG8183 |  |
| Cerebratulus orochi | OQ626348 |  |  |  |
| Cerebratulus sp. | EF124998 | GBSP14168-19 |  |  |
| Cerebratulus sp. | FJ811493 | GBSP2039-10 | BOLD:AAM3031 |  |
| Cerebratulus sp. | FJ811496 | GBSP2037-10 | BOLD:AAM2282 |  |
| Cerebratulus sp. | FJ811513 | GBSP2020-10 | BOLD:AAM2286 |  |
| Cerebratulus sp. | FJ811515 | GBSP2018-10 | BOLD:AAM2285 |  |
| Cerebratulus sp. | KP697722 | NORGE027-14 | BOLD:ACM5917 | NemBar1150 |
| Cerebratulus sp. | KP697723 | NORGE072-14 | BOLD:ACM5918 | NemBar1451 |
| Cerebratulus sp. | KP697724 | NORGE014-14 | BOLD:ACM5919 | NemBar1143 |
| Cerebratulus sp. | KP697726 | NORGE029-14 | BOLD:AAM2283 | NemBar1156 |
| Cerebratulus sp. | MT808211 | GBMND32473-21 | BOLD:AEH7814 |  |
| Cerebratulus sp. | MT808216 | GBMND32478-21 | BOLD:AEH7815 |  |
| Cerebratulus sp. | OK081718 | POIMB918-19 | BOLD:ADX8052 | BOIMB_1179 |
| Cerebratulus sp. | OM456683 | GBMNF77445-22 | BOLD:ABZ8354 |  |
| Cerebratulus sp. | OQ322814 |  |  |  |
| Cerebratulus sp. | OQ450482 |  |  |  |
| Cerebratulus sp. |  | BBPS1020-19 | BOLD:AEB5762 | 2980_DNA |
| Cerebratulus sp. |  | BBPS435-19 | [BOLD:AEB5649](https://bench.boldsystems.org/index.php/Public_BarcodeCluster?clusteruri=BOLD:AEB5649) | 4390_DNA |
| Cerebratulus sp. |  | BHAK2498-20 | BOLD:ADM2275 | BHAK-0978 |
| Cerebratulus sp. |  | ECHCA090-18 | BOLD:ADN6923 | CCDB-25432 H06 |
| Cerebratulus sp. |  | NLMAR543-20 | BOLD:AEG4759 | RMNH.5101616 |
| Cerebratulus sp. |  | QHAK4235-23 | BOLD:AAY6349 | ZHAK-03097 |
| Cerebratulus sp. |  | QHAK4250-23 | BOLD:AFI0228 | ZHAK-03112 |
| Cerebratulus sp. OR056 | KU197740 | ORNE187-21 | BOLD:AAE9633 | Pili_OR056_E4A4 |
| Cerebratulus sp. OR057 | KU197752 | ORNE193-21 | BOLD:ADO8699 | Pili_OR057_E1G5 |
| Cerebratulus sp. OR061 | KU197747 | ORNE202-21 | BOLD:AEA1764 | Pili_OR061_E3G4 |
| Cerebratulus sp. OR091 | KU197743 | ORNE388-21 | BOLD:AEK8038 | Pili_OR091_E1I1 |
| Cerebratulus sp. SMRS012 | N/A | NERS001-23 | BOLD:AFI9214 | AA_22_481 |
| Chernyshevia escarpiaphila | ON021860 |  |  |  |
| Corsoua sp. CB027 | MW021777 | CARNE100-19 | BOLD:AEB5198 | Pili_CB027_BdT005_3 |
| Corsoua sp. CB028 | MW021779 | CARNE108-19 | BOLD:AEB5199 | Pili_CB204_BdT018_1 |
| Corsoua sp. CB028 | MW021781 | CARNE106-19 | BOLD:AEB4784 | Pili_CB028_BdT005_1 |
| Corsoua sp. CB173 | OK081398 | CARNE441-21 | BOLD:AEB5755 | Pili_CB173_CB028_18_01 |
| Corsoua sp. CB174 | OK081640 | CARNE444-21 | BOLD:AEI8846 | Pili_CB174_CB028_18_04 |
| Corsoua takakurai | LC520106 | GBMNC35089-20 |  |  |
| Cratenemertidae sp. | KP270877 | GBMIN138463-18 | BOLD:ADL9233 |  |
| Cratenemertidae sp. CB024 | OK081738 | CARNE230-21 | BOLD:AEB7032 | Hoplo_CB024_CB150_18_01 |
| Dendrorhynchus sinensis | KC602702 | GBMAA1219-14 | BOLD:ACQ5960 |  |
| Dinonemertes arctica | FJ602542 | GBSP1802-10 | BOLD:AAX9568 |  |
| Diplomma cf. albimarginata | PP834711 | NOMAN210-23 | BOLD:AFB4366 | BOMAN-8059 |
| Diplomma polyophthalma | AB505816 | GBMAA203-14 | BOLD:ACQ1695 |  |
| Diplomma serpentina | MN205514 | GBMNC35164-20 | BOLD:AEG2032 |  |
| Diplomma serpentina | N/A | NERS096-23 | BOLD:ACQ1696 | BNOM_3061 |
| Diplomma serpentina | N/A | NEGAM002-24 | BOLD:ACQ1696 | GLTZ-0242 |
| Drepanophorus sp. SMGM007 | N/A | NEGAM010-24 | BOLD:AAY0563 | GLTZ-0258 |
| Drepanophorus sp. SMGM011 | N/A | NEGAM018-24 | BOLD:AGD9379 | GLTZ-0362 |
| Drepanophorus sp. SMOM022 | N/A | NERS014-23 | BOLD:AFA3451 | BDJRS_5025 |
| Drepanophorus sp. SMOM022 | PP834726 | NOMAN089-23 | BOLD:AFA3451 | BOMAN-2852 |
| Drepanophorus sp. SMOM088 | PP834732 | NOMAN254-23 | BOLD:AFJ0209 | BOMAN-15263 |
| Drepanophorus spectabilis | HQ848610 |  | BOLD:AEB1268 |  |
| Dushia atra | MW021801 |  | BOLD:ACB0189 |  |
| Dushia sp. | LC389855 | GBSP16734-19 | BOLD:AEB6511 |  |
| Dushia sp. | MN205501 | GBMNC35177-20 | BOLD:AEB1310 |  |
| Dushia sp. SMOM070 | PP834733 | NOMAN175-23 | BOLD:AFA8611 | BOMAN-12284 |
| Dushia sp. SMOM071 | PP834734 | NOMAN179-23 | BOLD:AFB4780 | BOMAN-3189 |
| Emplectonema buergeri |  | BHAK2543-20 | BOLD:AAX7485 | BHAK-8978 |
| Emplectonema gracile |  | GBMTG2032-16 | BOLD:AAF0221 | NC_016952 |
| Emplectonema mitsuii | MT776032 | GBMNF18480-22 | BOLD:AEY4673 |  |
| Emplectonema mitsuii | OM456699 | GBMNF77461-22 | BOLD:ACQ6446 |  |
| Emplectonema neesii |  | ADMAB302-23 | BOLD:AFK8712 | NHMO-DOT-988 |
| Emplectonema sp. |  | BHAK2537-20 | BOLD:AEC7939 | BHAK-5840 |
| Emplectonema viride | KU197597 | ORNE047-21 | BOLD:AAP1200 | Hoplo_OR017_E5B5 |
| Emplectonema viride |  | QHAK936-21 | BOLD:AEO1116 | QHAK-00779 |
| Emplectonematidae sp. | MT828541 | GBMNC35112-20 | BOLD:AEG9089 |  |
| Eousia aff. verticivaria | PP834740 | NOMAN145-23 | BOLD:AFB1082 | BOMAN-9085 |
| Eousia aff. verticivaria | PP834741 | NOMAN145-23 | BOLD:AFA8613 | BOMAN-10485 |
| Eousia sp. SMGM021 | N/A | NEGAM057-24 | BOLD:AEE4489 | GLTZ-0378 |
| Eousia sp. SMGM033 | N/A | NEGAM084-24 | BOLD:AGD9164 | GLTZ-2498 |
| Eousia sp. SMRS011 | N/A | NERS103-23 | BOLD:AFI9724 | BOIMB_2806 |
| Euborlasia maycoli | LC520108 | GBMNC35087-20 | BOLD:AEF9756 |  |
| Euborlasia sp. CB021 | MW021802 | CARNE088-19 | BOLD:AEB1506 | Pili_CB021_BdT034_1 |
| Euborlasia sp. ETP091 | N/A | NOPP227-23 | BOLD:AEN8556 | Pili_ETP091_SMPP0870 |
| Euborlasia sp. SMOM043 | PP834742 | NOMAN126-23 | BOLD:AFB3465 | BOMAN-7001 |
| Eumonostilifera gen.sp. SMOM025 | PP834745 | NOMAN097-23 | BOLD:AFA9738 | BOMAN-3186 |
| Eumonostilifera gen.sp. SMOM025 | PP834750 | NOMAN098-23 |  | BOMAN-8048 |
| Eumonostilifera gen.sp. SMOM028 | PP834751 | NOMAN106-23 | BOLD:AFA7799 | BOMAN-7008 |
| Eumonostilifera sp. | KJ592731 | SDP100035-13 | BOLD:ACM2303 | F12LK03 |
| Eumonostilifera sp. | ON021869 |  |  |  |
| Eumonostilifera sp. | ON021871 |  |  |  |
| Eumonostilifera sp. | PP652015 | MGHK753-21 | BOLD:AEL4071 | BHKG-3500 |
| Eumonostilifera sp. BOBA016 | OK081680 | ORNE038-21 |  | Hoplo_OR013_RE25III17_PgN |
| Eumonostilifera sp. BOBA020 | OQ075688 | NONEP054-21 | BOLD:AEJ7493 | BON_75_2020 |
| Eumonostilifera sp. CB061 | MW021884 | CARNE038-19 | BOLD:AEA9096 | Hoplo_CB061_BdT015_8 |
| Eumonostilifera sp. CB061 | MW021896 | CARNE040-19 | BOLD:AEA9097 | Hoplo_CB061_BdT037_1 |
| Eumonostilifera sp. CB062 | OK081412 | CARNE325-21 | BOLD:AEB6278 | Hoplo_CB062_CBdT0069 |
| Eumonostilifera sp. CB129 | OK081575 | CARNE357-21 | BOLD:AEI3272 | Hoplo_CB129_CB129_18_02 |
| Eumonostilifera sp. CB138 | OK081381 | CARNE373-21 | BOLD:AEI3273 | Hoplo_CB138_SMCP0016 |
| Eumonostilifera sp. CB156 | N/A | CARNE601-23 | BOLD:AFQ6985 | Hoplo_CB210_SMCP1418 |
| Eumonostilifera sp. CB156 | OK081733 | CARNE415-21 | BOLD:AEI8512 | Hoplo_CB156_CBdT0070 |
| Eumonostilifera sp. CB161 | OK081490 | CARNE421-21 | BOLD:AEI5851 | Hoplo_CB161_MCB010_18_01 |
| Eumonostilifera sp. CB162 | OK081758 | CARNE422-21 | BOLD:AEI3430 | Hoplo_CB162_MCB011_18_01 |
| Eumonostilifera sp. CB175 | OK081656 | CARNE445-21 | BOLD:AEI5857 | Hoplo_CB175_CB062_18_01 |
| Eumonostilifera sp. CB191 | OK081410 | CARNE469-21 | BOLD:AEI5852 | Hoplo_CB191_CBdT0018 |
| Eumonostilifera sp. CB192 | OK081696 | CARNE474-21 | BOLD:AEI5853 | Hoplo_CB192_SMCP0026 |
| Eumonostilifera sp. CB198 | OK081571 | CARNE490-21 | BOLD:AEI5856 | Hoplo_CB198_SMCP0250 |
| Eumonostilifera sp. CB205 | N/A | CARNE647-23 | BOLD:AFR4200 | Hoplo_CB205_SMCP1969 |
| Eumonostilifera sp. ETP074 | N/A | NOPP126-23 | BOLD:AFQ4860 | Hoplo_ETP074_SMPP0789 |
| Eumonostilifera sp. ETP075 | N/A | NOPP129-23 | BOLD:AFQ4861 | Hoplo_ETP075_SMPP0746 |
| Eumonostilifera sp. ETP078 | N/A | NOPP165-23 | BOLD:AFQ4862 | Hoplo_ETP078_SMPP0676 |
| Eumonostilifera sp. SMRS008 | N/A | NERS034-23 | BOLD:AFI9613 | BOIMB_2765 |
| Evelineus sp. | OM456684 | GBMNF77446-22 | BOLD:AEZ4656 |  |
| Evelineus sp. CB022 | MW021803 | CARNE091-19 | BOLD:AEB4971 | Pili_CB022_BdT031_1 |
| Evelineus sp. CB042 | MW021849 |  | BOLD:AEB3962 |  |
| Evelineus sp. CB136 | OK081677 | CARNE360-21 | BOLD:AEI3598 | Pili_CB136_CB136_18_01 |
| Fragilonemertes rosea | OQ322915 |  | BOLD:ACB0332 |  |
| Fragilonemertes_Cerebratulus leucopsis | OQ322705 |  | BOLD:ADZ5933 |  |
| Geonemertes pelaensis | EU255602 | GBSP5468-13 | BOLD:ACH2418 |  |
| Gononemertes parasita |  | GBMTG3990-16 | BOLD:ACQ1956 | NC_024822 |
| Gononemertes sp. | KP270878 | GBMIN138464-18 | BOLD:ADM0977 |  |
| Gononemertes sp. | MN205518 | GBMNC35160-20 | BOLD:AEG1655 |  |
| Gorgonorhynchus aff. repens | MN690210 | GBMNC35092-20 | BOLD:AEB0258 |  |
| Gorgonorhynchus aff. repens | PP834757 | NOMAN133-23 | BOLD:AFB2325 | BOMAN-3165 |
| Gorgonorhynchus aff. repens | PP834762 | NOMAN278-23 | BOLD:AFI9417 | BOMAN-17345 |
| Gorgonorhynchus albocinctus | N/A | NEGAM031-24 | BOLD:ACV7712 | GLTZ-2488 |
| Gorgonorhynchus sp. | HQ848632 |  |  |  |
| Gorgonorhynchus sp. | LC010649 | GBMIN44201-15 | BOLD:ACV7712 |  |
| Gorgonorhynchus sp. | LC520103 | GBMND99296-21 | BOLD:AEL3164 |  |
| Gorgonorhynchus sp. CB012 | MW021805 | CARNE083-19 | BOLD:ADW2695 | Pili_CB012_B1_15_867_080304_1 |
| Gorgonorhynchus sp. SMOM050 | PP834764 | NOMAN142-23 | BOLD:AFA3894 | BOMAN-9072 |
| Gorgonorhynchus sp. SMRS012 | N/A | NERS003-23 | BOLD:AFJ0163 | BDJRS_2656 |
| Gorgonorhynchus sp. SMRS013 | N/A | NERS008-23 | BOLD:AFI9289 | BDJRS_4430 |
| Gurjanovella littoralis | AJ436904 | GBSP0359-06 | BOLD:AAY2595 |  |
| Gurjanovella littoralis | LC505450 | GBMNB39711-20 | BOLD:AED3204 |  |
| Gurjanovella sp. | MF512123 | GBSP14771-19 | BOLD:AAP2666 |  |
| Gurjanovella sp. OR003 | MW396773 | ORNE030-21 | BOLD:AEL5254 | Hoplo_OR003_COIMB2116 |
| Heteronemertea gen. sp. | GU227121 |  |  |  |
| Heteronemertea gen. sp. | LC625640 | GBMNF18639-22 | BOLD:ADW0071 |  |
| Heteronemertea gen.sp. SMOM048 | PP834765 | NOMAN140-23 | BOLD:AFA6563 | BOMAN-10519 |
| Heteronemertea gen.sp. SMOM079 | PP834766 | NOMAN193-23 | BOLD:AFJ0555 | BOMAN-10505 |
| Heteronemertea sp. | KR606058 | GBSP14401-19 | BOLD:ADW8760 |  |
| Heteronemertea sp. | KU365724 | GBSP14196-19 | BOLD:ADW6395 |  |
| Heteronemertea sp. | MG421228 | BBAY134-13 | BOLD:ACW6225 | BIOUG06838-E08 |
| Heteronemertea sp. | MG422671 | BBAY136-13 | BOLD:ACI2722 | BIOUG06838-E10 |
| Heteronemertea sp. | MG423254 | OPQCS018-08 | BOLD:AAK0257 | HLC-24037 |
| Heteronemertea sp. | MH242860 |  | BOLD:ADM0201 |  |
| Heteronemertea sp. | MN794599 | ABBAI2293-17 | BOLD:ADE9513 | RCMBAR1225 |
| Heteronemertea sp. | MN794606 | ABBAI2294-17 | BOLD:ADF2093 | RCMBAR1226 |
| Heteronemertea sp. | MN794614 | ABBAI2311-17 | BOLD:ADE9514 | RCMBAR1243 |
| Heteronemertea sp. | MN794838 | ABBAI1265-16 | BOLD:ADF1025 | RCMBAR729 |
| Heteronemertea sp. | MN794843 | ABBAI503-15 | BOLD:ADF0859 | PHOTO_GAEZ_0038 |
| Heteronemertea sp. | MN794852 | ABBAI1370-16 | BOLD:ADF1022 | RCMBAR840 |
| Heteronemertea sp. | N/A | N/A | N/A | BMOO_08550 |
| Heteronemertea sp. |  | INV828-17 | BOLD:ADX9419 | CNP-Inv 2544 |
| Heteronemertea sp. |  | QHAK2485-22 | BOLD:AER8497 | QHAK-01482 |
| Heteronemertea sp. CB084 | MW021815 | CARNE206-19 | BOLD:AEA9039 | Pili_CB084_pilidium_014_2016 |
| Heteronemertea sp. CB159 | OK081403 | CARNE419-21 | BOLD:AEI1308 | Pili_CB159_MCB012_18_01 |
| Heteronemertea sp. OR035 | KU197832 | ORNE171-21 | BOLD:AEL6041 | Pili_OR035_E1G9 |
| Heteronemertea sp. OR058 | KU197829 | ORNE195-21 | BOLD:AEK4998 | Pili_OR058_E1H4 |
| Heteronemertea sp. OR092 | OK081426 | ORNE408-21 | BOLD:AAU0227 | Pili_OR092_934 |
| Heteronemertea sp. OR095 | KU197835 | ORNE402-21 | BOLD:AEL5990 | Pili_OR095_119 |
| Heteronemertea sp. OR098 | KU197831 | ORNE403-21 | BOLD:AEK4997 | Pili_OR098_E1H6 |
| Heteronemertes longifissa | OQ450486 |  | BOLD:ACM5920 |  |
| Heteronemertes longifissa | OQ450488 |  |  |  |
| Hinumanemertes kikuchii | GU392014 | GBSP4575-12 | BOLD:ACB4331 |  |
| Hoplonemertea gen. sp. | GU227122 |  |  |  |
| Hoplonemertea gen. sp. | LC625630 | GBMNF18628-22 | BOLD:AEU3344 |  |
| Hubrechtella cf. dubia | EF124984 | GBSP3711-12 |  |  |
| Hubrechtella dubia | KP697730 | NORGE088-14 | BOLD:AAD3042 | NemBar1126 |
| Hubrechtella ijimai | KY986685 | GBSP14782-19 | BOLD:ADW1472 |  |
| Hubrechtella ijimai | KY986686 | GBSP14783-19 | BOLD:ADW1473 |  |
| Hubrechtella juliae | KP270875 | GBSP10431-18 | BOLD:ACW7836 |  |
| Hubrechtella sp. | EF124999 | GBSP3715-12 | BOLD:ACB0063 |  |
| Hubrechtella sp. | MK047677 | GBSP17177-19 | BOLD:AEB6619 |  |
| Hubrechtella sp. | MN794586 | ABBAI263-15 | BOLD:ADE3502 | LV064 |
| Hubrechtella sp. | MN794794 | ABBAI1362-16 | BOLD:ADE0958 | RCMBAR832 |
| Hubrechtella sp. | MN794863 | ABBAI2412-17 | BOLD:ADJ4255 | RCMBAR2050 |
| Hubrechtella sp. CB026 | MW021824 | CARNE093-19 | BOLD:ACZ3139 | Pili_CB026_pilidium_005_2016 |
| Hubrechtella sp. SMOM060 | PP834772 | NOMAN163-23 | BOLD:AFB1359 | BOMAN-12288 |
| Iwatanemertes piperata |  | GBMTG5032-16 | BOLD:ADC7190 | NC_023523 |
| Kulikovia alborostrata | JN234382 | GBMNA12757-19 | BOLD:AAX5107 |  |
| Kulikovia cf. montgomeryi | OM456685 | GBMNF77447-22 | BOLD:AEC9556 |  |
| Kulikovia manchenkoi | HQ848574 |  | BOLD:ADX0055 |  |
| Kulikovia montgomeryi |  | QHAK2488-22 | BOLD:AAP2668 | QHAK-01486 |
| Kulikovia sp. BOBA003 | OQ075675 | NONEP023-21 | BOLD:ADX1401 | BON_03_2020 |
| Kulikovia sp. OR083 | KU197818 | ORNE307-21 | BOLD:AAP1137 | Pili_OR083_E1B6 |
| Kulikovia torquata | OR883927 |  | BOLD:ACB0058 |  |
| Leptonemertes cf. calicophora | HQ848596 |  |  |  |
| Leucocephalonemertes aurantiaca | GU392016 | GBSP4577-12 | BOLD:ACB4318 |  |
| Lineidae gen. sp. | LC190962 | GBMNF18640-22 | BOLD:AEU3017 |  |
| Lineidae gen. sp. | LC625623 | GBMNF18622-22 | BOLD:AEU3346 |  |
| Lineidae gen. sp. | LC625624 | GBMNF18623-22 | BOLD:AES5147 |  |
| Lineidae gen. sp. | LC625625 | GBMNF18624-22 | BOLD:AET5217 |  |
| Lineidae gen. sp. | LC625626 | GBMNF18625-22 | BOLD:AEU3343 |  |
| Lineidae gen. sp. | LC625627 | GBMNF18626-22 | BOLD:AET9254 |  |
| Lineidae gen. sp. | LC625628 | GBMNF18627-22 | BOLD:AER8856 |  |
| Lineidae gen. sp. | LC625636 | GBMNF18629-22 | BOLD:AER8855 |  |
| Lineidae gen. sp. | LC625637 | GBMNF18630-22 | BOLD:AEU3345 |  |
| Lineidae gen. sp. | LC625638 | GBMNF18631-22 | BOLD:AET3741 |  |
| Lineidae gen. sp. | LC625641 | GBMNF18632-22 | BOLD:AET8624 |  |
| Lineidae gen. sp. | LC625648 | GBMNF18635-22 | BOLD:AET5216 |  |
| Lineidae gen.sp. SMOM044 | PP834775 | NOMAN128-23 | BOLD:AFA3223 | BOMAN-12283 |
| Lineidae gen.sp. SMOM046 | PP834777 | NOMAN137-23 | BOLD:AFA3224 | BOMAN-9058 |
| Lineidae gen.sp. SMOM049 | PP834782 | NOMAN141-23 | BOLD:AFA4480 | BOMAN-11551 |
| Lineidae gen.sp. SMOM053 | PP834783 | NOMAN146-23 | BOLD:AFB1286 | BOMAN-11511 |
| Lineidae gen.sp. SMOM058 | PP834784 | NOMAN158-23 | BOLD:AFA4481 | BOMAN-8058 |
| Lineidae gen.sp. SMOM065 | PP834786 | NOMAN168-23 | BOLD:AFB1287 | BOMAN-11513 |
| Lineidae gen.sp. SMOM066 | PP834790 | NOMAN170-23 | BOLD:AFA8610 | BOMAN-10531 |
| Lineidae gen.sp. SMOM067 | PP834794 | NOMAN171-23 | BOLD:AFB0485 | BOMAN-8278 |
| Lineidae gen.sp. SMOM068 | PP834796 | NOMAN173-23 | BOLD:AFB1288 | BOMAN-3202 |
| Lineidae gen.sp. SMOM073 | PP834797 | NOMAN181-23 | BOLD:AFA3222 | BOMAN-8071 |
| Lineidae gen.sp. SMOM080 | PP834798 | NOMAN194-23 | BOLD:AFB0699 | BOMAN-11517 |
| Lineidae gen.sp. SMOM085 | PP834801 | NOMAN208-23 | BOLD:AFJ0539 | BOMAN-8043 |
| Lineidae gen.sp. SMOM086 | PP834803 | NOMAN221-23 | BOLD:AFJ0537 | BOMAN-10495 |
| Lineidae gen.sp. SMOM089 | PP834804 | NOMAN268-23 | BOLD:AFJ0554 | BOMAN-6898 |
| Lineidae gen.sp. SMOM092 | PP834805 | NOMAN264-23 | BOLD:AFJ0613 | BOMAN-16148 |
| Lineidae gen.sp. SMOM096 | PP834807 | NOMAN281-23 | BOLD:AFJ0538 | BOMAN-10507 |
| Lineidae gen.sp. SMOM097 | PP834808 | NOMAN279-23 | BOLD:AFI9736 | BOMAN-17669 |
| Lineidae gen.sp. SMOM098 | PP834809 | NOMAN297-24 |  | BOMAN-11521 |
| Lineidae gen.sp. SMOM099 | PP834810 | NOMAN287-24 |  | BOMAN-7055 |
| Lineidae gen.sp. SMOM100 | PP834812 | NOMAN284-24 |  | BOMAN-10539 |
| Lineidae sp. | GU227127 |  |  |  |
| Lineidae sp. | KC602704 | GBSP11715-19 | BOLD:ADW2934 |  |
| Lineidae sp. | KP270874 | GBMIN138465-18 | BOLD:ADM1993 |  |
| Lineidae sp. | KU197759 | GBSP14298-19 | BOLD:ADX1038 |  |
| Lineidae sp. | KU839778 |  |  |  |
| Lineidae sp. | MG422779 | ARCMI354-14 | BOLD:AAP2670 | BIOUG14667-F11 |
| Lineidae sp. | MG423559 | BBAY206-14 | BOLD:ACN3404 | BIOUG12670-C10 |
| Lineidae sp. | MN205497 | GBMNC35181-20 | BOLD:AEG9474 |  |
| Lineidae sp. | MN205503 | GBMNC35175-20 | BOLD:AEG6695 |  |
| Lineidae sp. | MN794646 | ABBAI1733-16 | BOLD:ADE1361 | RCMB0285 |
| Lineidae sp. | MT999960 | HIMF040-20 | BOLD:AEE4489 | USNM1616794 |
| Lineidae sp. | MW021831 |  |  |  |
| Lineidae sp. | MW021836 |  |  |  |
| Lineidae sp. | MW021858 |  |  |  |
| Lineidae sp. | MW021860 |  |  |  |
| Lineidae sp. | MW021864 |  |  |  |
| Lineidae sp. | OM456686 | GBMNF77448-22 | BOLD:AEX0519 |  |
| Lineidae sp. | OM456688 | GBMNF77450-22 | BOLD:AEY9047 |  |
| Lineidae sp. | OM456689 | GBMNF77451-22 | BOLD:AEZ3431 |  |
| Lineidae sp. | OM456690 | GBMNF77452-22 | BOLD:AEZ1150 |  |
| Lineidae sp. | OM456691 | GBMNF77453-22 | BOLD:AEZ7050 |  |
| Lineidae sp. | OM456692 | GBMNF77454-22 | BOLD:AEW6654 |  |
| Lineidae sp. | OM456693 | GBMNF77455-22 | BOLD:AEZ8612 |  |
| Lineidae sp. | ON021872 |  |  |  |
| Lineidae sp. | P1020925 | ZPC950-20 | BOLD:AEC7943 | BIOUG19287 H09 |
| Lineidae sp. |  | DISA534-19 | BOLD:ADR8066 | LACM:DISCO:3241 |
| Lineidae sp. |  | DISA924-19 | BOLD:ADR9816 | LACM:DISCO:7300 |
| Lineidae sp. |  | INV779-17 | BOLD:ADY0748 | CNP-Inv 2495 |
| Lineidae sp. |  | INV819-17 | BOLD:ADY2256 | CNP-Inv 2535 |
| Lineidae sp. |  | LABBI067-09 | BOLD:AAG3612 | TBLABR-067 |
| Lineidae sp. |  | LABBI070-09 | BOLD:AEH1786 | TBLABR-070 |
| Lineidae sp. CB0136 | MN794610 | ABBAI1020-16 | BOLD:ADI8471 | RCMBAR466 |
| Lineidae sp. CB014 | MW021869 | CARNE086-19 | BOLD:AEB5356 | Pili_CB014_B1_44_840_080103_1 |
| Lineidae sp. CB015 | OK081443 | CARNE448-21 | BOLD:AEI0700 | Pili_CB178_CB014_18_01b |
| Lineidae sp. CB035 | OK081341 | CARNE259-21 | BOLD:AEA9020 | Pili_CB035_CB035_18_09 |
| Lineidae sp. CB037 | KF935511 | GBSP11473-19 | BOLD:ADL4245 |  |
| Lineidae sp. CB039 | OK081338 | CARNE266-21 | BOLD:AEI0698 | Pili_CB039_SMCP0090 |
| Lineidae sp. CB041 | OK081710 | CARNE269-21 |  | Pili_CB041_CB041_18_03 |
| Lineidae sp. CB045 | OK081791 | CARNE289-21 | BOLD:AEA9018 | Pili_CB045_CB047_18_03 |
| Lineidae sp. CB046 | MW021845 | CARNE158-19 | BOLD:AEB5760 | Pili_CB046_BdT007_3 |
| Lineidae sp. CB047 | OK081550 | CARNE299-21 | BOLD:AEA9017 | Pili_CB047_CB047_18_01 |
| Lineidae sp. CB048 | OK081516 | CARNE300-21 | BOLD:AEB5354 | Pili_CB048_CB045_18_09 |
| Lineidae sp. CB049 | OK081808 | CARNE306-21 | BOLD:AEB5427 | Pili_CB049_CB049_18_09 |
| Lineidae sp. CB050 | KF935513 | GBSP11471-19 | BOLD:ADW9829 |  |
| Lineidae sp. CB052 | MW021834 | CARNE178-19 | BOLD:AEA9019 | Pili_CB052_BdT026_3 |
| Lineidae sp. CB086 | MW021813 | CARNE209-19 | BOLD:AEA9038 | Pili_CB086_pilidium_030_2016 |
| Lineidae sp. CB102 | MN794840 | ABBAI2519-17 | BOLD:ADF0175 | RCMBAR2163 |
| Lineidae sp. CB137 | OK081361 | CARNE365-21 | BOLD:AEI4864 | Pili_CB137_CB036_18_05 |
| Lineidae sp. CB142 | OK081389 | CARNE385-21 | BOLD:AEI4866 | Pili_CB142_CBdT0065 |
| Lineidae sp. CB144 | OK081787 | CARNE388-21 | BOLD:AEI9690 | Pili_CB144_CB052_18_02 |
| Lineidae sp. CB145 | OK081478 | CARNE392-21 | BOLD:AEI4865 | Pili_CB145_CBdT0047 |
| Lineidae sp. CB147 | OK081345 | CARNE397-21 | BOLD:AEI9688 | Pili_CB147_SMCP0285 |
| Lineidae sp. CB148 | N/A | NECOL006-23 | BOLD:AFP9770 | Co_Lin_002 |
| Lineidae sp. CB149 | OK081383 | CARNE401-21 | BOLD:AEI9689 | Pili_CB149_SMCP0286 |
| Lineidae sp. CB152 | OK081621 | CARNE409-21 | BOLD:AEI0699 | Pili_CB152_SMCP0281 |
| Lineidae sp. CB181 | N/A | NECOL010-23 | BOLD:AFP9733 | Co_Mic_003 |
| Lineidae sp. CB182 | N/A | CARNE645-23 |  | Pili_CB182_SMCP1410 |
| Lineidae sp. CB196 | OK081465 | CARNE487-21 | BOLD:AEI4153 | Pili_CB196_SMCP0108 |
| Lineidae sp. CB199 | OK081458 | CARNE491-21 | BOLD:AEI8134 | Pili_CB199_CBdT0087 |
| Lineidae sp. CB200 | OK081752 | CARNE493-21 | BOLD:AEI8133 | Pili_CB200_SMCP0268 |
| Lineidae sp. CB201 | OK081515 | CARNE494-21 | BOLD:AEI5919 | Pili_CB201_SMCP0192 |
| Lineidae sp. CB209 | N/A | CARNE576-23 |  | Pili_CB209_CBdT0081 |
| Lineidae sp. ETP014 | OK081540 | NOPP023-21 | BOLD:AEK4780 | ETP014_SMPP0911 |
| Lineidae sp. ETP015 | OK081530 | NOPP030-21 | BOLD:AEK7581 | ETP015_SMPP0862 |
| Lineidae sp. ETP020 | OK081387 | NOPP038-21 | BOLD:AEK7582 | ETP020_B1_76 |
| Lineidae sp. ETP021 | OK081630 | NOPP039-21 | BOLD:AEL5224 | ETP021_B1_62 |
| Lineidae sp. ETP022 | OK081789 | NOPP043-21 | BOLD:AEL8178 | ETP022_B1_77 |
| Lineidae sp. ETP023 | OK081420 | NOPP044-21 | BOLD:AEK5285 | ETP023_B1_57 |
| Lineidae sp. ETP024 | N/A | NOPP201-23 | BOLD:AFQ6887 | Pili_ETP024_B2_A3 |
| Lineidae sp. ETP025 | OK081689 | NOPP045-21 | BOLD:ADE1179 | ETP025_B1_56 |
| Lineidae sp. ETP026 | OK081709 | NOPP047-21 | BOLD:AEK5286 | ETP026_B1_73 |
| Lineidae sp. ETP027 | N/A | NOPP202-23 | BOLD:AFQ6886 | Pili_ETP027_SMPP0653 |
| Lineidae sp. ETP028 | OK081612 | NOPP049-21 | BOLD:ADY0932 | ETP028_SMPP0655 |
| Lineidae sp. ETP030 | OK081692 | NOPP053-21 |  | ETP030_SMPP0652 |
| Lineidae sp. ETP032 | OK081533 | NOPP054-21 | BOLD:AEL5223 | ETP032_SMPP0737 |
| Lineidae sp. ETP034 | OK081682 | NOPP056-21 | BOLD:AEK9680 | ETP034_B2_A1 |
| Lineidae sp. ETP035 | N/A | NOPP203-23 | BOLD:AFR7983 | Pili_ETP035_B2_A2 |
| Lineidae sp. ETP036 | OK081495 | NOPP058-21 | BOLD:AEK4779 | ETP036_SMPP0736 |
| Lineidae sp. ETP037 | OK081445 | NOPP060-21 | BOLD:AEK4781 | ETP037_B1_80 |
| Lineidae sp. ETP038 | MN794630 | ABBAK019-17 | BOLD:ADK9792 | RCMB0619 |
| Lineidae sp. ETP039 | OK081401 | NOPP062-21 | BOLD:AEL3583 | ETP039_B1_59 |
| Lineidae sp. ETP048 | N/A | NOPP206-23 | BOLD:AFQ6885 | Pili_ETP048_SMPP0884 |
| Lineidae sp. ETP049 | N/A | NOPP207-23 | BOLD:AFR5954 | Pili_ETP049_SMPP0735 |
| Lineidae sp. ETP050 | N/A | NOPP208-23 | BOLD:AFQ6884 | Pili_ETP050_SMPP0803 |
| Lineidae sp. ETP051 | OK081806 | NOPP071-21 | BOLD:AEL1689 | ETP051_SMPP0807 |
| Lineidae sp. ETP054 | N/A | NOPP216-23 | BOLD:AFR7982 | Pili_ETP054_SMPP0805 |
| Lineidae sp. ETP054 | OK081810 | NOPP078-21 | BOLD:AEL2322 | ETP054_SMPP0670 |
| Lineidae sp. ETP055 | OK081510 | NOPP079-21 | BOLD:AEK4327 | ETP055_SMPP0630 |
| Lineidae sp. ETP090 | N/A | NOPP225-23 | BOLD:AFR8393 | Pili_ETP090_SMPP0665 |
| Lineidae sp. OR101 | OK081636 | ORNE407-21 | BOLD:AEL1688 | Pili_OR101_NHL418 |
| Lineidae sp. SMGM010 | N/A | NEGAM017-24 | BOLD:AGD4696 | GLTZ-0361 |
| Lineidae sp. SMGM012 | N/A | NEGAM067-24 | BOLD:AEG7165 | GLTZ-0402 |
| Lineidae sp. SMGM014 | N/A | NEGAM083-24 | BOLD:AGD4056 | GLTZ-2497 |
| Lineidae sp. SMGM019 | N/A | NEGAM032-24 | BOLD:AGD4698 | GLTZ-2491 |
| Lineidae sp. SMGM024 | N/A | NEGAM066-24 |  | GLTZ-0401 |
| Lineidae sp. SMGM025 | N/A | NEGAM082-24 | BOLD:AGD4697 | GLTZ-2495 |
| Lineidae sp. SMGM026 | N/A | NEGAM065-24 | BOLD:AGD4693 | GLTZ-0398 |
| Lineidae sp. SMGM027 | N/A | NEGAM079-24 | BOLD:AGD4692 | GLTZ-2486 |
| Lineidae sp. SMGM028 | N/A | NEGAM073-24 | BOLD:AGD4695 | GLTZ-0416 |
| Lineidae sp. SMGM029 | N/A | NEGAM075-24 | BOLD:AGD4694 | GLTZ-2477 |
| Lineidae sp. SMOM058 | N/A | NEGAM081-24 | BOLD:AFA4481 | GLTZ-2493 |
| Lineidae sp. SMOM067 | N/A | NEGAM028-24 | BOLD:AFB0485 | GLTZ-2480 |
| Lineidae sp. SMRS010 | N/A | NERS011-23 | BOLD:AFI9735 | BDJRS_4785 |
| Lineidae sp. SMRS014 | N/A | NERS017-23 | BOLD:AFI9734 | BDJRS_6191 |
| Lineidae sp. SMRS019 | N/A | NERS048-23 | BOLD:AFI9733 | BOIMB_2785 |
| Lineus bilineatus | EF124969 | GBSP3699-12 | BOLD:ACB0347 |  |
| Lineus bilineatus | GU392015 | GBSP4576-12 | BOLD:AAC5473 |  |
| Lineus clandestinus | MK047694 | GBSP17194-19 | BOLD:AAN6732 |  |
| Lineus longissimus | MK047697 | GBSP17197-19 | BOLD:AAX5078 |  |
| Lineus pictifrons |  | DISCT004-17 | BOLD:ADH5578 | LACM:DISCO:3481 |
| Lineus ruber | MK047693 | GBSP17193-19 | BOLD:AAX5077 |  |
| Lineus rubescens | KU197785 | ORNE379-21 | BOLD:ADW8928 | Pili_OR088_E2I9 |
| Lineus rubescens | KU197790 | ORNE373-21 | BOLD:ADS0049 | Pili_OR087_E3F5 |
| Lineus sanguineus | MK047714 | GBSP17214-19 | BOLD:AAC3224 |  |
| Lineus sp. | LC553818 | GBMND32485-21 | BOLD:AEH8007 |  |
| Lineus sp. | LC553819 | GBMND32486-21 | BOLD:AEH3361 |  |
| Lineus sp. | LC553821 | GBMND32488-21 | BOLD:AEH3362 |  |
| Lineus sp. | MH235867 |  |  |  |
| Lineus sp. | MZ558339 | GBMNE6999-21 | BOLD:AEN5900 |  |
| Lineus sp. | OK081650 | POIMB213-19 | BOLD:ADX6613 | BOIMB_0437 |
| Lineus sp. |  | QHAK2968-23 | BOLD:ADX6613 | QHAK-01938 |
| Lineus sp. OR074 | KU197804 | ORNE264-21 | BOLD:AEL2623 | Pili_OR074_E3C8 |
| Lineus sp. OR075 | KU197814 | ORNE270-21 | BOLD:AEK3943 | Pili_OR075_E2H3 |
| Lineus viridis | FJ839919 | GBMNA12758-19 | BOLD:AAK8914 |  |
| Lineus viridis |  | WSVAR002-09 | BOLD:AFJ4546 | WS112 |
| Maculaura alaskensis | KP682072 | ORNE336-21 | BOLD:AAX4262 | Pili_OR084_OR_S1_E3B8 |
| Maculaura aquilonia | KP682084 | GBMIN138687-18 | BOLD:ADL9780 |  |
| Maculaura aquilonia | KP682133 | GBMIN138689-18 | BOLD:ADL9779 |  |
| Maculaura cerebrosa | OK081519 | POIMB1013-19 | BOLD:AAP1201 | BOIMB_1464 |
| Maculaura magna | KP682150 | ORNE301-21 | BOLD:AEK7678 | Pili_OR081_OR_C3_93 |
| Maculaura magna | KP682152 | ORNE303-21 | BOLD:AEK7679 | Pili_OR082_OR_C4_112 |
| Maculaura magna | OK081783 | POIMB362-19 | BOLD:ADL9679 | BOIMB_0454 |
| Maculaura magna | KP682154 | ORNE298-21 | BOLD:ADX7722 | Pili_OR080_OR_C5_163 |
| Maculaura oregonensis | KP682158 | ORNE339-21 | BOLD:ADM2641 | Pili_OR085_OR_C1_E4A2 |
| Maculaura sp. | OQ450493 |  |  |  |
| Malacobdella arrokeana | JX220616 | GBSP8734-13 | BOLD:ACH5500 |  |
| Malacobdella grossa |  | NLMAR580-20 | BOLD:AAB1714 | RMNH.5101662 |
| Malacobdella japonica | KF597261 | GBMIN138732-18 | BOLD:ADM3058 |  |
| Malacobdella siliquae | KU197601 | ORNE102-21 | BOLD:AEK8982 | Hoplo_OR046_E4B2 |
| Micrura akkeshiensis | EF124975 | GBSP3704-12 | BOLD:ACA9930 |  |
| Micrura bathyalis | MN205502 | GBMNC35176-20 | BOLD:AEG5820 |  |
| Micrura bella | OQ450494 |  |  |  |
| Micrura bella |  | GBMTG5676-16 | BOLD:ACI3089 | NC_027727 |
| Micrura callima | EF124976 | GBSP3705-12 | BOLD:ACA9931 |  |
| Micrura callima | MN205496 | GBMNC35182-20 | BOLD:AEG7165 |  |
| Micrura callima | MW278386 | GBMND32492-21 | BOLD:ADX8185 |  |
| Micrura cf. aurantiaca | N/A | NERS047-23 | BOLD:AFJ0414 | BOIMB_2783 |
| Micrura chlorapardalis | MK047690 | GBSP17190-19 | BOLD:ADW5336 |  |
| Micrura dellechiajei | KP893673 | GBSP11462-19 | BOLD:ADW3505 |  |
| Micrura fasciolata | GU392022 | GBSP4583-12 | BOLD:AAD5268 |  |
| Micrura fasciolata |  | ADMAB091-23 | BOLD:ACM6287 | NHMO-DOT-721 |
| Micrura formosana | EF124978 | GBSP14171-19 |  |  |
| Micrura ignea | KP115292 | GBMNA12759-19 | BOLD:ADC8526 |  |
| Micrura leidyi | KU906113 | SERCI326-14 | BOLD:ACM1454 | SERCINVERT0326 |
| Micrura purpurea | GU392019 | GBSP4580-12 | BOLD:AAC4377 |  |
| Micrura rubramaculosa | OK081370 | CARNE250-21 | BOLD:AEB6432 | Pili_CB033_SMCP0269 |
| Micrura sp. | EF124979 | GBSP3707-12 |  |  |
| Micrura sp. | KF935510 | GBSP11470-19 | BOLD:ADW4115 |  |
| Micrura sp. | OM456696 | GBMNF77458-22 | BOLD:AEY1881 |  |
| Micrura sp. | OQ417231 |  |  |  |
| Micrura sp. |  | BBPS434-19 | BOLD:AEA8956 | 4401_DNA |
| Micrura sp. |  | BBPS854-19 | BOLD:AEA8955 | 3288_DNA |
| Micrura sp. OR064 | KU197826 | ORNE217-21 | BOLD:ADW5343 | Pili_OR064_E4H8 |
| Micrura sp. OR067 | KU197841 | ORNE222-21 | BOLD:ADW4042 | Pili_OR067_E1H8 |
| Micrura sp. OR068 | KU197857 | ORNE226-21 | BOLD:AEK6814 | Pili_OR068_E3B2 |
| Micrura sp. OR069 | KU197847 | ORNE230-21 | BOLD:AEL3801 | Pili_OR069_E2C4 |
| Micrura sp. OR070 | KU197842 | ORNE234-21 | BOLD:ADM7414 | Pili_OR070_142 |
| Micrura sp. OR071 | KU197850 | ORNE237-21 | BOLD:AEK6813 | Pili_OR071_E3B1 |
| Micrura sp. OR072 | KX342092 | ORNE243-21 | BOLD:ADM3288 | Pili_OR072_N3_A |
| Micrura sp. SMGM012 | N/A | NEGAM020-24 | BOLD:AEG7165 | GLTZ-0381 |
| Micrura sp. SMGM014 | N/A | NEGAM024-24 | BOLD:AGD4056 | GLTZ-0415 |
| Micrura varicolor | KP697754 | NORGE038-14 | BOLD:ACM5324 | NemBar1295 |
| Micrura verrilli | OK081405 | POIMB221-19 | BOLD:ADW4746 | BOIMB_0425 |
| Micrura verrilli |  | BHAK2468-20 | BOLD:AEC7028 | BHAK-0506 |
| Micrura wilsoni | OK081325 | POIMB600-19 | BOLD:ADW9830 | BOIMB_0478 |
| Micrurides albopunctatus | GU392027 | GBSP4588-12 | BOLD:ACB5342 |  |
| Monostilifera sp. | EU489489 |  |  |  |
| Monostilifera sp. | KP270880 | GBMIN138741-18 | BOLD:ADM2367 |  |
| Monostilifera sp. | KP270882 | GBMIN138740-18 | BOLD:ADM2365 |  |
| Monostilifera sp. | KU839773 |  |  |  |
| Monostilifera sp. | MZ216521 | GBMNF18578-22 | BOLD:AEU4491 |  |
| Monostilifera sp. | MZ580728 | DUTCH208-19 | BOLD:AEC4254 | DUT_1050 |
| Monostilifera sp. |  | DISA1000-19 | BOLD:ADR7154 | LACM:DISCO:7709 |
| Monostilifera sp. |  | INTGS091-22 | BOLD:AEO1136 | NEM_C_0689.4 |
| Monostilifera sp. |  | INV771-17 | BOLD:ADY2209 | CNP-Inv 2487 |
| Monostilifera sp. |  | INV783-17 | BOLD:ADY0121 | CNP-Inv 2499 |
| Monostilifera sp. |  | INV787-17 | BOLD:ADY0021 | CNP-Inv 2503 |
| Monostilifera sp. |  | INV803-17 | BOLD:ADY0222 | CNP-Inv 2519 |
| Monostilifera sp. |  | INV843-17 | BOLD:ADX9447 | CNP-Inv 2559 |
| Monostilifera sp. | OM456732 | GBMNF77494-22 | BOLD:AEY7136 |  |
| Monostilifera sp. CB190 | N/A | NECOL046-23 | BOLD:AFO6074 | Co_Mon_014 |
| Nectonemertes cf. mirabilis | MF512124 | GBSP14772-19 | BOLD:AAX3456 |  |
| Nemertea sp. | KU839780 |  |  |  |
| Nemertea sp. | N/A | N/A | N/A | BMOO_06148 |
| Nemertea sp. | N/A | N/A | N/A | BMOO_08564 |
| Nemertea sp. | N/A | N/A | N/A | BMOO_08745 |
| Nemertea sp. | N/A | N/A | N/A | BMOO_09786 |
| Nemertea sp. | N/A | N/A | N/A | BMOO_10094 |
| Nemertea sp. | N/A | N/A | N/A | BMOO_15117 |
| Nemertea sp. | N/A | N/A | N/A | BMOO_15830 |
| Nemertea sp. | N/A | N/A | N/A | BMOO_16582 |
| Nemertea sp. | N/A | N/A | N/A | BMOO_16824 |
| Nemertea sp. | N/A | N/A | N/A | BMOO_16855 |
| Nemertea sp. | N/A | N/A | N/A | BMOO_19040 |
| Nemertea sp. | N/A | N/A | N/A | DL323 |
| Nemertea sp. | N/A | N/A | N/A | XMOO_0218 |
| Nemertellina sp. | N/A | NERS097-23 | BOLD:AFI9671 | BNOM_3082 |
| Nemertellina sp. BOBA011 | OQ075689 | NONEP052-21 | BOLD:AEJ4336 | BON_69_2020 |
| Nemertellina sp. CB0211 | N/A | CARNE590-23 | BOLD:AFQ8404 | Hoplo_CB211_SMCP0375 |
| Nemertellina sp. CB160 | OK081779 | CARNE420-21 | BOLD:AEI3161 | Hoplo_CB160_MCB007_18_01 |
| Nemertellina sp. CB206 | N/A | CARNE622-23 | BOLD:AFR5442 | Hoplo_CB206_SMCP1968 |
| Nemertellina sp. ETP058 | OK081597 | NOPP086-21 | BOLD:AEK3903 | ETP058_SMPP0642 |
| Nemertellina sp. ETP083 | N/A | NOPP172-23 | BOLD:AFQ8404 | Hoplo_ETP083_SMPP0693 |
| Nemertellina sp. SMGM002 | N/A | NEGAM004-24 | BOLD:AGD3531 | GLTZ-0249 |
| Nemertellina sp. SMOM021 | PP834820 | NOMAN081-23 | BOLD:AFB1635 | BOMAN-7059 |
| Nemertellina sp. SMRS009 | N/A | NERS081-23 | BOLD:AFJ0233 | BNOM_1851 |
| Nemertellina sp. SMRS018 | N/A | NERS024-23 | BOLD:AFI9666 | BOIMB_2787 |
| Nemertellina sp. SMRS018 | N/A | NERS042-23 | BOLD:AFI9667 | BOIMB_2775 |
| Nemertellina sp. SMRS025 | N/A | NERS068-23 | BOLD:AFI9668 | BNOM_0966 |
| Nemertellina sp. SMRS026 | N/A | NERS071-23 | BOLD:AFI9670 | BNOM_0969 |
| Nemertellina sp. SMRS028 | N/A | NERS074-23 | BOLD:AFI9669 | BNOM_1001 |
| Nemertellina yamaokai | AJ436907 | GBSP0362-06 | BOLD:AAX6040 |  |
| Nemertopsis bivittata | KX377861 | GBSP14447-19 | BOLD:ADX2239 |  |
| Nemertopsis bivittata | MW065563 | GBMNF74319-22 | BOLD:AEZ5911 |  |
| Nemertopsis sp. | KF728379 | GBMAA1577-15 | BOLD:ACS4400 |  |
| Nemertopsis sp. | MK047679 | GBSP17179-19 | BOLD:AEB1699 |  |
| Nemertopsis sp. | MN690179 | GBMNC35095-20 | BOLD:AEA9854 |  |
| Nemertopsis sp. | MN794777 | ABBAI317-15 | BOLD:ADE9451 | LVSF037 |
| Nemertopsis sp. | MT433990 | GBMNF18502-22 | BOLD:AEU4967 |  |
| Nemertopsis sp. | MT433991 | GBMNF18503-22 | BOLD:AEU4966 |  |
| Nemertopsis sp. | MT433995 | GBMNF18507-22 | BOLD:AEU4965 |  |
| Nemertopsis sp. | MT433996 | GBMNF18508-22 | BOLD:AET3997 |  |
| Nemertopsis sp. | MT433997 | GBMNF18509-22 | BOLD:ADG1226 |  |
| Nemertopsis sp. CB146 | AJ436908 | GBSP0363-06 | BOLD:AAX6059 |  |
| Nemertopsis sp. SMOM030 | PP834822 | NOMAN110-23 | BOLD:AFA8144 | BOMAN-1241 |
| Nemertopsis tetraclitophila | KF572482 | GBMNA12765-19 | BOLD:ADC6251 |  |
| Nemertovema hadalis | KY296912 | GBSP14445-19 |  |  |
| Nemertovema norenburgi | MN205523 | GBMNC35155-20 | BOLD:AEG6447 |  |
| Nipponnemertes arenaria | OM456701 | GBMNF77463-22 | BOLD:AEG7149 |  |
| Nipponnemertes bimaculata | PP464300 | WANEM002-22 | BOLD:AEW1521 | BFHL5974 |
| Nipponnemertes bimaculata |  | BHAK2509-20 | BOLD:AAX6404 | BHAK-0492 |
| Nipponnemertes cf. madagascarensis | PP834823 | NOMAN111-23 | BOLD:AFA2388 | BOMAN-7007 |
| Nipponnemertes incainca | OK081430 | CARNE329-21 | BOLD:ADX6536 | Hoplo_CB063_SMCP0284 |
| Nipponnemertes ogumai | AB920907 | GBMAA618-14 |  |  |
| Nipponnemertes ogumai | LC677144 | GBMNF18465-22 | BOLD:AET2215 |  |
| Nipponnemertes pulchra | KP697762 | NORGE085-14 | BOLD:AAE2013 | NemBar1449 |
| Nipponnemertes punctatula | KC710980 | GBMNA12763-19 | BOLD:AAX6405 |  |
| Nipponnemertes sp. | HQ848599 |  |  |  |
| Nipponnemertes sp. | MH106528 | GBSP16931-19 | BOLD:AEB2033 |  |
| Nipponnemertes sp. | OM456698 | GBMNF77460-22 | BOLD:AEG5361 |  |
| Nipponnemertes sp. | OM456702 | GBMNF77464-22 | BOLD:AEG6548 |  |
| Nipponnemertes sp. | OM456716 | GBMNF77478-22 | BOLD:AEW6851 |  |
| Nipponnemertes sp. | OM456718 | GBMNF77480-22 | BOLD:AEZ6636 |  |
| Nipponnemertes sp. | OM456719 | GBMNF77481-22 | BOLD:AEM4628 |  |
| Nipponnemertes sp. | OM456720 | GBMNF77482-22 | BOLD:AEY4498 |  |
| Nipponnemertes sp. | ON357589 |  |  |  |
| Nipponnemertes sp. | ON357606 | GBMNF78013-22 | BOLD:AEX9768 |  |
| Nipponnemertes sp. | ON357614 | GBMNF78016-22 | BOLD:AEX7958 |  |
| Nipponnemertes sp. | OP104817 |  | BOLD:ADY0746 |  |
| Nipponnemertes sp. |  | INTGS089-22 | BOLD:AEN9545 | NEM_B_0168.3 |
| Nipponnemertes sp. |  | INV795-17 | BOLD:ADY0746 | CNP-Inv 2511 |
| Nipponnemertes sp. |  | INV799-17 | BOLD:ADX9763 | CNP-Inv 2515 |
| Nipponnemertes sp. BOBA028 | OQ075692 | NONEP068-21 | BOLD:AEJ7531 | BON_95_2021 |
| Nipponnemertes sp. CB015 | N/A | CARNE616-23 | BOLD:AFR4014 | Hoplo_CB015_SMCP1958 |
| Nipponnemertes sp. CB016 | MW021788 | CARNE009-19 | BOLD:AEB4937 | Hoplo_CB016_BdT009_6 |
| Nipponnemertes sp. CB017 | MW021786 | CARNE014-19 | BOLD:AEB3485 | Hoplo_CB017_BdT010_2 |
| Nipponnemertes sp. CB018 | MW021794 | CARNE015-19 | BOLD:AEB4938 | Hoplo_CB018_BdT010_3 |
| Nipponnemertes sp. CB025 | OK081756 | CARNE232-21 | BOLD:AEB1182 | Hoplo_CB025_CB025_18_01 |
| Nipponnemertes sp. CB177 | N/A | NECOL050-23 | BOLD:AFN7537 | Co_Nip_003 |
| Nipponnemertes sp. CB177 | OK081523 | CARNE446-21 | BOLD:AEI9845 | Hoplo_CB177_CB017_18_01 |
| Nipponnemertes sp. CB188 | N/A | NECOL004-23 | BOLD:AFP8267 | Co_Dis_001 |
| Nipponnemertes sp. CB188 | N/A | NECOL055-23 | BOLD:AEB6750 | Co_Rep_003 |
| Nipponnemertes sp. ETP056 | OK081753 | NOPP081-21 | BOLD:AEK5429 | ETP056_SMPP0794 |
| Nipponnemertes sp. ETP057 | OK081506 | NOPP083-21 | BOLD:AEK5430 | ETP057_SMPP0798 |
| Nipponnemertes sp. ETP086 | N/A | NOPP175-23 | BOLD:AFR4795 | Hoplo_ETP086_SMPP0011 |
| Nipponnemertes sp. g | ON357598 | GBMNF77993-22 | BOLD:AEY5967 |  |
| Nipponnemertes sp. j | ON357625 | GBMNF77992-22 | BOLD:AEX9349 |  |
| Nipponnemertes sp. l | ON357603 | GBMNF78011-22 | BOLD:AEX6451 |  |
| Nipponnemertes sp. n | ON357599 | GBMNF78007-22 | BOLD:AEY9558 |  |
| Nipponnemertes sp. o | ON357602 | GBMNF78010-22 | BOLD:AEX4725 |  |
| Nipponnemertes sp. SMGM003 | N/A | NEGAM009-24 | BOLD:AGD3301 | GLTZ-0257 |
| Nipponnemertes sp. SMOM023 | PP834826 | NOMAN091-23 | BOLD:AFA2387 | BOMAN-8292 |
| Nipponnemertes sp. SMOM024 | PP834828 | NOMAN092-23 | BOLD:AFB3551 | BOMAN-9063 |
| Nipponnemertes sp. SMOM084 | PP834829 | NOMAN203-23 | BOLD:AFJ0458 | BOMAN-7044 |
| Nipponnemertes sp. SMOM094 | PP834830 | NOMAN255-23 | BOLD:AFJ0664 | BOMAN-15268 |
| Nipponnemertes sp. SMRS005 | N/A | NERS009-23 | BOLD:AFJ0665 | BDRJS_4631 |
| Nipponnemertes sp. SMRS006 | N/A | NERS064-23 | BOLD:AFI9271 | BOIMB_2830 |
| NipponnemertesAmphiporus hastatus | KP697710 | NORGE078-14 | BOLD:ACM5027 | NemBar1457b |
| Nipponomicrura sp. | OM456695 | GBMNF77457-22 | BOLD:AEY7451 |  |
| Nipponomicrura sp. | OQ450496 |  |  |  |
| Nipponomicrura uchidai | KY561815 | GBSP14781-19 | BOLD:ADX4076 |  |
| NOT_Prosorhochmus nelsoni | MK047678 | GBSP17178-19 | BOLD:AEB2022 |  |
| Notospermus aff. tricuspidatus | N/A | NERS106-23 | BOLD:AFB1537 | BOIMB_2828 |
| Notospermus aff. tricuspidatus | PP834834 | NOMAN206-23 | BOLD:AFB1537 | BOMAN-8028 |
| Notospermus albocinctus | OK081502 | CARNE330-21 | BOLD:AEB6982 | Pili_CB064_CB064_18_02 |
| Notospermus geniculatus | EF125002 | GBSP3717-12 | BOLD:AAX5105 |  |
| Notospermus geniculatus | LC625629 | GBMNF18618-22 | BOLD:AAX5105 |  |
| Notospermus geniculatus | MH714705 | GBSP17134-19 | BOLD:AEB6981 |  |
| Notospermus sp. | KF935515 | GBSP11474-19 | BOLD:ADW5640 |  |
| Notospermus sp. | KF935516 | GBSP11449-19 | BOLD:ADW5641 |  |
| Notospermus sp. | MW021901 |  |  |  |
| Notospermus sp. | MW021904 |  |  |  |
| Notospermus sp. CB066 | MW021903 | CARNE191-19 | BOLD:AEB5852 | Pili_CB066_BdT028_1 |
| Notospermus sp. ETP033 | N/A | NOPP223-23 | BOLD:AFR8898 | Pili_ETP033_SMPP0009 |
| Notospermus sp. ETP033 | OK081413 | NOPP055-21 | BOLD:AEK8186 | ETP033_B1_67 |
| Notospermus sp. SMGM009 | N/A | NEGAM016-24 | BOLD:AGD3267 | GLTZ-0288 |
| Notospermus sp. SMGM030 | N/A | NEGAM076-24 | BOLD:AGD3268 | GLTZ-2478 |
| Notospermus sp. SMGM031 | N/A | NEGAM078-24 | BOLD:AGD3266 | GLTZ-2484 |
| Notospermus sp. SMOM056 | N/A | NERS012-23 | BOLD:AFJ0114 | BDJRS_4844 |
| Notospermus sp. SMOM056 | N/A | NERS016-23 | BOLD:AFB1538 | BDJRS_6125 |
| Notospermus sp. SMOM056 | PP834843 | NOMAN154-23 | BOLD:AFB1538 | BOMAN-8027 |
| Notospermus sp. SMOM057 | PP834847 | NOMAN156-23 | BOLD:AFA6759 | BOMAN-9062 |
| Notospermus tricuspidatus | EF124973 | GBSP3702-12 | BOLD:ACB0152 |  |
| Notospermus tricuspidatus | N/A | NEGAM072-24 | BOLD:ACB0152 | GLTZ-0414 |
| Oerstedia aff. dorsalis | EU489488 |  | BOLD:AAM1387 |  |
| Oerstedia aff. dorsalis | KP697773 | NORGE060-14 | BOLD:AAB9744 | NemBar1442a |
| Oerstedia aff. dorsalis | MZ216522 | GBMNF18513-22 | BOLD:AES4466 |  |
| Oerstedia aff. dorsalis | MZ216523 | GBMNF18512-22 | BOLD:AES4465 |  |
| Oerstedia dorsalis | FJ855306 | GBSP1784-10 | BOLD:AAM1387 |  |
| Oerstedia dorsalis | FJ855330 | GBSP1760-10 | BOLD:AAM1386 |  |
| Oerstedia dorsalis | FJ855333 | GBSP1757-10 | BOLD:AAM1385 |  |
| Oerstedia dorsalis | FJ855336 | GBSP1754-10 | BOLD:AAB9743 |  |
| Oerstedia dorsalis | KF935536 | GBSP11465-19 | BOLD:AAM1389 |  |
| Oerstedia dorsalis | OM456721 | GBMNF77483-22 | BOLD:AAM1388 |  |
| Oerstedia dorsalis |  | ADMAB228-23 | BOLD:AAB9742 | NHMO-DOT-814 |
| Oerstedia fuscosparsa | OP265741 |  |  |  |
| Oerstedia oculata | MN205512 | GBMNC35166-20 | BOLD:AEG6052 |  |
| Oerstedia phoresiae | MN205513 | GBMNC35165-20 | BOLD:AEG7355 |  |
| Oerstedia polyorbis | MZ216524 | GBMNF18514-22 | BOLD:AET8118 |  |
| Oerstedia sp. | AJ436912 | GBSP0367-06 | BOLD:AAW9899 |  |
| Oerstedia sp. | KF935535 | GBSP11467-19 | BOLD:ADW5273 |  |
| Oerstedia sp. | MG422137 | CCANN209-08 | BOLD:AAG3610 | 08PROBE-0120 |
| Oerstedia sp. |  | BHAK2530-20 | BOLD:AEC9750 | BHAK-3646 |
| Oerstedia sp. |  | BHAK2542-20 | BOLD:AEC7534 | BHAK-8823 |
| Oerstedia sp. |  | DISA789-19 | BOLD:ADR7531 | LACM:DISCO:6472 |
| Oerstedia sp. |  | INV801-17 | BOLD:ADY2257 | CNP-Inv 2517 |
| Oerstedia sp. |  | INV834-17 | BOLD:ADY5557 | CNP-Inv 2550 |
| Oerstedia sp. | OM456722 | GBMNF77484-22 | BOLD:AEY0918 |  |
| Oerstedia sp. | OM456723 | GBMNF77485-22 | BOLD:AEZ5142 |  |
| Oerstedia sp. | OM456724 | GBMNF77486-22 | BOLD:AEZ6228 |  |
| Oerstedia sp. | OM456725 | GBMNF77487-22 | BOLD:AEW6391 |  |
| Oerstedia sp. | OM456726 | GBMNF77488-22 | BOLD:AEY6359 |  |
| Oerstedia sp. | OM456727 | GBMNF77489-22 | BOLD:AEX4285 |  |
| Oerstedia sp. | OM456728 | GBMNF77490-22 | BOLD:AEZ8056 |  |
| Oerstedia sp. | OM456729 | GBMNF77491-22 | BOLD:AEZ3176 |  |
| Oerstedia sp. | OM456731 | GBMNF77493-22 | BOLD:AEZ4955 |  |
| Oerstedia sp. | OM456733 | GBMNF77495-22 | BOLD:AEX5781 |  |
| Oerstedia sp. | OM456734 | GBMNF77496-22 | BOLD:AEX0956 |  |
| Oerstedia sp. BOBA022 | OQ075694 | NONEP032-21 | BOLD:AEJ2779 | BON_32_2020 |
| Oerstedia sp. ETP093 | N/A | NOPP177-23 | BOLD:AFQ3746 | Hoplo_ETP085_SMPP0888 |
| Oerstedia sp. Iturup | MZ216525 | GBMNF18515-22 | BOLD:AET8117 |  |
| Oerstedia sp. SMOM029 | PP834849 | NOMAN107-23 | BOLD:AFA5574 | BOMAN-7002 |
| Oerstediidae sp. | MN205516 | GBMNC35162-20 | BOLD:AEG8111 |  |
| Oerstediidae sp. | ON255709 | GBMNF77946-22 | BOLD:AEX9852 |  |
| Oerstediidae sp. | OP034708 | GBMNF78952-22 | BOLD:AEZ0106 |  |
| Ototyphlonemertes aff. duplex | KU230158 |  | BOLD:ACQ7534 |  |
| Ototyphlonemertes aff. erneba | KM083858 | GBMAA219-14 | BOLD:ACQ2292 |  |
| Ototyphlonemertes aff. erneba | KM083869 | GBMAA688-14 | BOLD:ACQ2289 |  |
| Ototyphlonemertes ani | LC311004 | GBSP11611-19 | BOLD:ADM3130 |  |
| Ototyphlonemertes chernyshevi | LC310990 | GBSP11625-19 | BOLD:ADW0549 |  |
| Ototyphlonemertes correae | KU230204 |  | BOLD:AEB4220 |  |
| Ototyphlonemertes dorsalis | KU230238 | GBSP17891-21 | BOLD:ACQ7535 |  |
| Ototyphlonemertes duplex | KU230168 |  |  |  |
| Ototyphlonemertes duplex | KU230177 |  |  |  |
| Ototyphlonemertes duplex | KU230182 |  |  |  |
| Ototyphlonemertes duplex | KU230241 |  | BOLD:AEE2116 |  |
| Ototyphlonemertes envalli | LC310996 | GBSP11619-19 | BOLD:ADX2062 |  |
| Ototyphlonemertes erneba | KM083861 | GBMAA1333-14 | BOLD:ACQ2290 |  |
| Ototyphlonemertes erneba | KM083871 | GBMAA1334-14 | BOLD:ACQ2288 |  |
| Ototyphlonemertes erneba | KT722713 | GBMIN138754-18 | BOLD:ADM1546 |  |
| Ototyphlonemertes erneba | KT722719 | GBMIN138752-18 | BOLD:ACQ2293 |  |
| Ototyphlonemertes erneba | KT722722 | GBMIN138758-18 | BOLD:ADM1545 |  |
| Ototyphlonemertes erneba | KT722724 | GBMIN138751-18 | BOLD:ADM1547 |  |
| Ototyphlonemertes erneba | KT722727 | GBMIN138747-18 | BOLD:ACS6391 |  |
| Ototyphlonemertes erneba | KT722728 | GBSP14543-19 | BOLD:ADW8131 |  |
| Ototyphlonemertes erneba | KT730596 | GBMIN138882-18 | BOLD:ACQ2291 |  |
| Ototyphlonemertes fila | KT730641 | GBMIN138763-18 | BOLD:ADM2260 |  |
| Ototyphlonemertes fila | KT730659 | GBSP14579-19 | BOLD:ADX6897 |  |
| Ototyphlonemertes fila | KT730662 | GBMIN138760-18 | BOLD:ACQ7945 |  |
| Ototyphlonemertes fila | KT730666 | GBMIN138765-18 | BOLD:ADL9201 |  |
| Ototyphlonemertes fila | KT730668 | GBSP14535-19 | BOLD:ADW0159 |  |
| Ototyphlonemertes fila | MW000005 | HIMF077-20 | BOLD:AEE3345 | USNM1616751 |
| Ototyphlonemertes fila | MW000008 | HIMF044-20 | BOLD:AEE4436 | USNM1616784 |
| Ototyphlonemertes fila | OM836544 | GBMNF77724-22 | BOLD:ADX6898 |  |
| Ototyphlonemertes lactea | KU230050 | GBMIN138825-18 | BOLD:ADM3129 |  |
| Ototyphlonemertes lactea | KU230070 | GBSP14484-19 | BOLD:ADW5071 |  |
| Ototyphlonemertes lactea | KU230075 | GBSP14547-19 | BOLD:ADM3128 |  |
| Ototyphlonemertes lactea | KU230091 | GBMIN138795-18 | BOLD:ACQ0217 |  |
| Ototyphlonemertes lactea | KU230104 | GBSP14551-19 | BOLD:ACQ0218 |  |
| Ototyphlonemertes lactea | KU230126 | GBSP14556-19 | BOLD:ADW5070 |  |
| Ototyphlonemertes lactea | KU230290 | GBMIN138867-18 | BOLD:ADM2142 |  |
| Ototyphlonemertes lactea | KX377862 | GBSP14461-19 | BOLD:ADW5069 |  |
| Ototyphlonemertes lactea | MG926537 | GBSP15001-19 |  |  |
| Ototyphlonemertes lactea | MG926538 | GBSP15002-19 |  |  |
| Ototyphlonemertes lactea | MG926541 | GBSP15005-19 |  |  |
| Ototyphlonemertes lei | LC311007 | GBSP11608-19 | BOLD:ADV9732 |  |
| Ototyphlonemertes longissima | MK045324 | GBSP17174-19 | BOLD:AEB5742 |  |
| Ototyphlonemertes macintoshi | KM083877 | GBMAA1989-15 |  |  |
| Ototyphlonemertes macintoshi | KU230250 | GBMIN138846-18 | BOLD:ADM2143 |  |
| Ototyphlonemertes macintoshi | KU230255 | GBMIN138863-18 | BOLD:ADM2141 |  |
| Ototyphlonemertes macintoshi | KU230258 | GBSP14472-19 | BOLD:ADW5535 |  |
| Ototyphlonemertes macintoshi | KU230279 | GBMIN138865-18 | BOLD:ACQ7628 |  |
| Ototyphlonemertes macintoshi | KU230287 | GBMIN138842-18 | BOLD:ADM2140 |  |
| Ototyphlonemertes macintoshi | KU230288 | GBMIN138843-18 |  |  |
| Ototyphlonemertes macintoshi | MW000010 | HIMF075-20 | BOLD:AEE4899 | USNM1616749 |
| Ototyphlonemertes martynovi | OM832255 |  |  |  |
| Ototyphlonemertes nakaoae | LC311008 | GBSP11607-19 | BOLD:ADW2464 |  |
| Ototyphlonemertes norenburgi | LC310998 | GBSP11617-19 | BOLD:ADV9645 |  |
| Ototyphlonemertes pallida | KT736311 | GBSP14540-19 | BOLD:ADM2500 |  |
| Ototyphlonemertes pallida | KT736314 | GBSP14481-19 | BOLD:ADX6388 |  |
| Ototyphlonemertes pallida | MG926513 | GBSP15010-19 |  |  |
| Ototyphlonemertes pallida | MG926535 | GBSP15032-19 |  |  |
| Ototyphlonemertes pallida | MG926536 | GBSP15033-19 |  |  |
| Ototyphlonemertes pallida_mcintoshi | KF935545 | GBSP11452-19 | BOLD:ADM2139 |  |
| Ototyphlonemertes santacruzensis | KT730600 | GBMIN138885-18 | BOLD:ADM1100 |  |
| Ototyphlonemertes santacruzensis | KT730601 | GBSP14491-19 | BOLD:ADX4281 |  |
| Ototyphlonemertes santacruzensis | KT730607 | GBMIN138883-18 | BOLD:ADM1099 |  |
| Ototyphlonemertes santacruzensis | KT730621 | GBMIN138880-18 | BOLD:ACQ7039 |  |
| Ototyphlonemertes santacruzensis | KT730623 | GBSP14478-19 | BOLD:ADX3403 |  |
| Ototyphlonemertes santacruzensis | KT730625 | GBMIN138890-18 | BOLD:ACQ7037 |  |
| Ototyphlonemertes sp. | KU230044 | GBMIN138743-18 | BOLD:ADM3125 |  |
| Ototyphlonemertes sp. | KU230117 | GBMIN138745-18 | BOLD:ADM3127 |  |
| Ototyphlonemertes sp. | MG421301 | NNOP067-08 | BOLD:AAP2672 | HLC-30497 |
| Ototyphlonemertes sp. | MT999992 | HIMF078-20 | BOLD:AEE8653 | USNM1616738 |
| Ototyphlonemertes sp. | OM836527 | GBMNF77708-22 | BOLD:AEZ6631 |  |
| Ototyphlonemertes sp. | OM836531 | GBMNF77712-22 | BOLD:AEW7525 |  |
| Ototyphlonemertes sp. | OM836540 | GBMNF77721-22 | BOLD:AEX8094 |  |
| Ototyphlonemertes sp. | OM836543 | GBMNF77726-22 | BOLD:AEW7939 |  |
| Ototyphlonemertes sp. | OM836551 | GBMNF77732-22 | BOLD:AEY5668 |  |
| Ototyphlonemertes sp. | OM836553 | GBMNF77734-22 | BOLD:AEX7551 |  |
| Ototyphlonemertes sp. | OM836556 | GBMNF77737-22 | BOLD:ADW7218 |  |
| Ototyphlonemertes sp. BOBA030 | OQ075696 | NONEP056-21 | BOLD:ADM3126 | BON_77_2020 |
| Ototyphlonemertes sp. CB077 | OK081713 | CARNE332-21 | BOLD:ACQ7038 | Hoplo_CB077_MCB003_18_02 |
| Ototyphlonemertes sp. CB157 | OK081642 | CARNE416-21 | BOLD:AEI4005 | Hoplo_CB157_MCB001_18_01 |
| Ototyphlonemertes sp. OR016 | OK081472 | ORNE044-21 | BOLD:AEL4572 | Hoplo_OR016_3_MMB2020 |
| Ototyphlonemertes sp. OR096 | MW396776 | ORNE103-21 | BOLD:AEK9060 | Hoplo_OR096_COIMB2113 |
| Ototyphlonemertes sp. SMOM091 | PP834852 | NOMAN263-23 | BOLD:AFI9497 | BOMAN-16140 |
| Ototyphlonemertes tsukagoshii | LC215924 | GBSP14737-19 | BOLD:ADW1147 |  |
| Ovicides paralithodis | AB704417 | GBSP7994-13 | BOLD:ACI1851 |  |
| Oxypolella sp. | KP697714 | NORGE091-14 | BOLD:ACM5178 | NemBar1138 |
| Oxypolella sp. CB081 | MW021909 | CARNE196-19 | BOLD:AEB4325 | Pili_CB081_BdT012_2 |
| Oxypolella sp. SMOM072 | PP834854 | NOMAN180-23 | BOLD:AFB4858 | BOMAN-8320 |
| Oxypolella sp. SMRS17 | N/A | NERS027-23 | BOLD:AFI9548 | BOIMB_2815 |
| Oxypollella sp. CB080 | MW021908 | CARNE194-19 | BOLD:AEB4326 | Pili_CB080_BdT012_1 |
| Palaeonemertea sp. | MN794709 | ABBAI1757-16 | BOLD:ADE0350 | RCMB0315 |
| Palaeonemertea sp. | MN794801 | ABBAI1642-16 | BOLD:ADF1023 | RCMB0188 |
| Palaeonemertea sp. | N/A | N/A | N/A | BMOO_09147 |
| Paleonemertea sp. | KU839781 |  |  |  |
| Paleonemertea sp. |  | BBPS1071-19 | BOLD:AEA9462 | 4199_DNA |
| Paleonemertea sp. |  | BBPS121-19 | BOLD:AEA9045 | 3204_DNA |
| Paleonemertea sp. |  | BBPS214-19 | BOLD:AEA9855 | 3112_DNA |
| Pantinonemertes californiensis | MK047685 | GBSP17185-19 | BOLD:ACH3573 |  |
| Paradrepanophorus crassus | HQ848603 |  | BOLD:AEB0246 |  |
| Parahubrechtia peri | MZ772875 |  |  |  |
| Parahubrechtia rayi | EU489499 |  | BOLD:ADW5486 |  |
| Paranemertes aff. peregrina |  | BHAK2478-20 | BOLD:AAL3452 | BHAK-0487 |
| Paranemertes californica | MW396761 | ORNE014-21 | BOLD:AEL4847 | Hoplo_OR002_21III19_5 |
| Paranemertes peregrina | OK081395 | POIMB365-19 | BOLD:ADM2436 | BOIMB_0463 |
| Paranemertes peregrina | OK081435 | ORNE036-21 | BOLD:AEK2895 | Hoplo_OR011_REgg27ii12 |
| Paranemertes peregrina |  | GBMTG1807-16 | BOLD:ADC7199 | NC_014865 |
| Paranemertes sanjuanensis | MK047686 | GBSP17186-19 | BOLD:AAX2483 |  |
| Paranemertes sp. | AJ436916 | GBSP0371-06 | BOLD:AAX2484 |  |
| Paranemertes sp. | KP200118 | GBSP14626-19 | BOLD:ADM0220 |  |
| Paranemertes sp. | KP200127 | GBSP14639-19 | BOLD:ADX3762 |  |
| Paranemertes sp. BOBA009 | KU197625 | ORNE034-21 | BOLD:ADM0221 | Hoplo_OR010_E3D1 |
| Paranemertes sp. OR001 | KU197613 | ORNE004-21 | BOLD:AEK4918 | Hoplo_OR001_338 |
| Parborlasia corrugatus | MH630149 | GBSP12113-19 | BOLD:AAL3220 |  |
| Parborlasia corrugatus |  | INV814-17 | BOLD:AAM1958 | CNP-Inv 2530 |
| Parvicirrus dubius | AJ436940 | GBSP0395-06 | BOLD:AAX1659 |  |
| Parvicirrus sp. | GU227124 |  |  |  |
| Pelagica sp. | HM907538_SUPPRESSED | CMARA058-09 | BOLD:ACX7557 | Ne05.2.1 |
| Pelagica sp. | HQ941674_SUPPRESSED | CMARA045-09 | BOLD:ACX6726 | Ne07.1.1 |
| Pelagica sp. | MZ848450 | GBMNE7011-21 | BOLD:AEM9172 |  |
| Pelagica sp. | MZ848492 | GBMNE7012-21 | BOLD:AEM9786 |  |
| Pelagica sp. | MZ848497 | GBMNE7013-21 | BOLD:AEN8011 |  |
| Pelagica sp. | MZ848595 | GBMNE7010-21 | BOLD:AEN4717 |  |
| Pelagica sp. | OK209471 | GBMNE7014-21 | BOLD:AEN6759 |  |
| Pelagica sp. | OK340102 | GBMNE7016-21 | BOLD:AEN4718 |  |
| Pelagica sp. | OK340103 | GBMNE7017-21 | BOLD:AEN1064 |  |
| Pelagica sp. |  | GBMTG2585-16 | BOLD:AAY2697 | NC_017874 |
| Pelagonemertes sp. | AJ436924 | GBSP0379-06 | BOLD:AAX1425 |  |
| Pilidiophora gen.sp. SMOM093 | PP834855 | NOMAN262-23 | BOLD:AFI9777 | BOMAN-16129 |
| Pilidiophora sp. | MN794590 | ABBAI1830-16 |  | RCMB0388 |
| Pilidiophora sp. | MN794633 | ABBAI2107-17 | BOLD:ADF0176 | RCMBAR1027 |
| Pilidiophora sp. | MN794634 | ABBAI755-16 | BOLD:ADE1649 | RCMBAR183 |
| Pilidiophora sp. | MN794659 | ABBAI839-16 | BOLD:ADE5004 | RCMBAR273 |
| Pilidiophora sp. | MN794679 | ABBAI2421-17 | BOLD:ADE5549 | RCMBAR2059 |
| Pilidiophora sp. | MN794689 | ABBAI757-16 | BOLD:ADE1650 | RCMBAR185 |
| Pilidiophora sp. | MN794693 | ABBAI1732-16 | BOLD:ADF1024 | RCMB0284 |
| Pilidiophora sp. | MN794694 | ABBAI2099-17 | BOLD:ADJ1305 | RCMBAR1019 |
| Pilidiophora sp. | MN794695 | ABBAI1833-16 | BOLD:ADF0860 | RCMB0391 |
| Pilidiophora sp. | MN794711 | ABBAI1626-16 | BOLD:ADF1027 | RCMB0172 |
| Pilidiophora sp. | MN794714 | ABBAI486-15 | BOLD:ACB0097 | PHOTO_GAEZ_0021 |
| Pilidiophora sp. | MN794722 | ABBAI935-16 | BOLD:ADF0858 | RCMBAR375 |
| Pilidiophora sp. | MN794733 | ABBAI1358-16 | BOLD:ADE3503 | RCMBAR828 |
| Pilidiophora sp. | MN794755 | ABBAI1359-16 | BOLD:ADF0298 | RCMBAR829 |
| Pilidiophora sp. | MN794758 | ABBAI1361-16 | BOLD:ADE0959 | RCMBAR831 |
| Pilidiophora sp. | MN794760 | ABBAI270-15 | BOLD:ADF0857 | LV070 |
| Pilidiophora sp. | MN794769 | ABBAI2395-17 | BOLD:ADJ3366 | RCMBAR2033 |
| Pilidiophora sp. | MN794783 | ABBAI758-16 | BOLD:ADE5005 | RCMBAR186 |
| Pilidiophora sp. | MN794790 | ABBAI1364-16 | BOLD:ADE0960 | RCMBAR834 |
| Pilidiophora sp. | MN794791 | ABBAI2103-17 | BOLD:ADK9397 | RCMBAR1023 |
| Pilidiophora sp. | MN794815 | ABBAI1360-16 | BOLD:ADE1178 | RCMBAR830 |
| Pilidiophora sp. | MN794825 | ABBAI534-15 | BOLD:ADE9375 | PHOTO_GAEZ_0069 |
| Pilidiophora sp. | MN794865 | ABBAK016-17 | BOLD:ADF1026 | RCMB0616 |
| Pilidiophora sp. |  | DISCT006-17 |  | LACM:DISCO:6515 |
| Plectonemertidae sp. | KF935526 | GBSP11436-19 | BOLD:ADW7158 |  |
| Plectonemertidae sp. | KF935528 | GBSP11433-19 | BOLD:ADW7160 |  |
| Plectonemertidae sp. | KF935529 | GBSP11429-19 | BOLD:ADW7162 |  |
| Plectonemertidae sp. | KF935530 | GBSP11428-19 | BOLD:ADW6071 |  |
| Plectonemertidae sp. | KF935531 | GBSP11427-19 | BOLD:ADW7161 |  |
| Plectonemertidae sp. | KF935532 | GBSP11430-19 | BOLD:ADW7163 |  |
| Poseidonemertes collaris | KU197629 | ORNE080-21 | BOLD:AAW9336 | Hoplo_OR030_149 |
| Poseidonemertes maslakovae | KP270879 | GBMIN138974-18 | BOLD:ADM1076 |  |
| Poseidonemertes sp. | AJ436918 | GBSP0373-06 | BOLD:AAY3150 |  |
| Poseidonemertes sp. | GU392028 | GBSP4589-12 | BOLD:ACB4845 |  |
| Poseidonemertes sp. | KJ592728 | SDP100031-13 | BOLD:ACM2302 | F12LK02 |
| Poseidonemertes sp. | MK047683 | GBSP17183-19 | BOLD:AEB3654 |  |
| Poseidonemertes sp. | MN794672 | ABBAI1646-16 | BOLD:ADE9512 | RCMB0192 |
| Poseidonemertes sp. | OQ323281 |  |  |  |
| Poseidonemertes sp. | OQ323332 |  |  |  |
| Poseidonemertes sp. |  | BBPS042-19 | BOLD:AEB6995 | 3678_DNA |
| Poseidonemertes sp. BOBA010 | OQ075700 | NONEP014-21 | BOLD:AEK1697 | B14_2019 |
| Poseidonemertes sp. BOBA033 | OQ075699 | NONEP034-21 | BOLD:AEK1698 | BON_35_2020 |
| Poseidonemertes sp. CB186 | N/A | NECOL064-23 | BOLD:AFN3677 | Co_Tet_006 |
| Poseidonemertes sp. CB207 | N/A | CARNE627-23 | BOLD:AFQ9618 | Hoplo_CB207_SMCP1981 |
| Poseidonemertes sp. ETP003 | OK081622 | NOPP003-21 | BOLD:AAY3149 | ETP003_B2_A8 |
| Poseidonemertes sp. ETP044 | OK081513 | NOPP070-21 | BOLD:AEL2282 | ETP044_B1_68 |
| Poseidonemertes sp. ETP044 | OK081790 | NOPP005-21 | BOLD:AEL2281 | ETP004_B1_50 |
| Poseidonemertes sp. ETP079 | N/A | NOPP166-23 | BOLD:AFQ3284 | Hoplo_ETP079_SMPP0749 |
| Poseidonemertes sp. ETP088 | N/A | NOPP176-23 | BOLD:AFR3682 | Hoplo_ETP088_SMPP0755 |
| Poseidonemertes sp. SMOM061 | PP834856 | NOMAN164-23 | BOLD:AFA8707 | BOMAN-10496 |
| Poseidonemertes sp. SMOM062 | PP834857 | NOMAN165-23 | BOLD:AFA8706 | BOMAN-10534 |
| Potamostoma shizunaiense | PP211017 |  |  |  |
| Procephalothrix sp. | GU227123 |  |  |  |
| Prosadenoporus floridensis | EF157596 | GBSP5819-13 | BOLD:AAX2670 |  |
| Prosadenoporus floridensis | OK081531 | CARNE335-21 | BOLD:AEI6081 | Hoplo_CB105_CB105_18_01 |
| Prosadenoporus mooreae | EF157595 | GBSP5820-13 | BOLD:ACH3034 |  |
| Prosadenoporus mortoni | EF157593 | GBSP5822-13 | BOLD:ACH3033 |  |
| Prosadenoporus sp. SMOM010 | PP834858 | NOMAN034-23 | BOLD:AFB0431 | BOMAN-11519 |
| Prosadenoporus sp. SMOM011 | N/A | NERS019-23 | BOLD:AFJ0609 | BOIMB_2762 |
| Prosadenoporus sp. SMOM011 | PP834859 | NOMAN035-23 | BOLD:AFB0430 | BOMAN-11550 |
| Prosadenoporus spectaculum |  | GBMTG4388-16 | BOLD:ADC8690 | NC_023931 |
| Prosadenoporus winsori | EF157594 | GBSP5821-13 | BOLD:ACH3032 |  |
| Prosorhochmus belizeanus | EF157591 | GBSP5824-13 | BOLD:ACH2692 |  |
| Prosorhochmus claparedii | EF157588 | GBSP5827-13 | BOLD:ACH3999 |  |
| Prosorhochmus nelsoni | EF157586 | GBSP5829-13 | BOLD:ACH2880 |  |
| Prosorhochmus sp. ETP073 | N/A | NOPP123-23 | BOLD:AFQ4736 | Hoplo_ETP073_SMPP0750 |
| Prostoma cf. eilhardi | MK047681 | GBSP17181-19 | BOLD:AAG3607 |  |
| Prostoma graecense |  | ANNMO647-20 | BOLD:ACH2584 | BSC-234.1 |
| Prostoma sp. | HQ938796 | CFWIA605-10 | BOLD:AAN8900 | 10-SCCWRP-0605 |
| Protopelagonemertes beebei | LC565011 | GBMNF18519-22 | BOLD:ACB4034 |  |
| Protopelagonemertes sp. | AJ436927 | GBSP0382-06 | BOLD:AAX8574 |  |
| Psammamphiporus elongatus | HQ848609 |  |  |  |
| Pseudomicrura afzelii | GU392012 | GBSP4573-12 | BOLD:ACB4196 |  |
| Pseudomicrura afzelii | KP697775 | NORGE031-14 | BOLD:ACM5493 | NemBar1166 |
| Quasitetrastemma nigrifrons | MZ216528 | GBMNF18572-22 | BOLD:AES1150 |  |
| Quasitetrastemma nigrifrons | MZ580737 | DUTCH209-19 | BOLD:ADX0572 | DUT_1051 |
| Quasitetrastemma stimpsoni | KP270885 | GBMIN138978-18 | BOLD:ADM1957 |  |
| Ramphogordius lacteus | KR606057 | GBMIN138980-18 | BOLD:AAD6274 |  |
| Ramphogordius lacteus | MK047698 | GBSP17198-19 | BOLD:ADW7605 |  |
| Reptantia gen.sp. SMRS023 | N/A | NERS076-23 | BOLD:AFI9659 | BNOM_1286 |
| Reptantia sp. | AJ436929 | GBSP0384-06 | BOLD:AAY0563 |  |
| Reptantia sp. | AJ436930 | GBSP0385-06 | BOLD:AAY0564 |  |
| Reptantia sp. | KP270876 | GBSP11422-19 | BOLD:ADW1311 |  |
| Reptantia sp. | MN205505 | GBMNC35173-20 | BOLD:AEG6849 |  |
| Reptantia sp. | MN205506 | GBMNC35172-20 | BOLD:AEF9856 |  |
| Reptantia sp. | MN205507 | GBMNC35171-20 | BOLD:AEG9829 |  |
| Reptantia sp. | ON021870 |  |  |  |
| Reptantia sp. CB107 | MW021919 | CARNE047-19 | BOLD:AEB6001 | Hoplo_CB107_BdT024_1 |
| Reptantia sp. CB108 | MW021920 | CARNE048-19 | BOLD:AEB6002 | Hoplo_CB108_BdT024_2 |
| Reptantia sp. CB109 | MW021912 | CARNE052-19 | BOLD:AEB4809 | Hoplo_CB109_BdT016_2 |
| Reptantia sp. CB110 | KF935523 | GBSP11457-19 | BOLD:ADW1312 |  |
| Reptantia sp. CB111 | KF935522 | GBSP11475-19 | BOLD:AAZ2976 |  |
| Reptantia sp. CB179 | N/A | CARNE595-23 | BOLD:AFP5303 | Hoplo_CB179_SMCP1404 |
| Reptantia sp. CB179 | N/A | NECOL059-23 | BOLD:AFP5303 | Co_Rep_010 |
| Reptantia sp. CB187 | N/A | NECOL057-23 | BOLD:AFP5302 | Co_Rep_007 |
| Reptantia sp. ETP080 | N/A | NOPP171-23 | BOLD:AFG3208 | Hoplo_ETP080_SMPP0875 |
| Reptantia sp. SMRS003 | N/A | NERS002-23 | BOLD:AFI9661 | BDJRS2016_5570 |
| Reptantia sp. SMRS004 | N/A | NERS066-23 | BOLD:AFI9660 | BOIMB_2833 |
| Riseriellus occultus | HQ848582 |  | BOLD:AAC3225 |  |
| Riserius pugetensis | AJ436941 | GBSP0396-06 | BOLD:AAX8883 |  |
| Riserius sp. | MW000051 | HIMF038-20 | BOLD:AEE1207 | USNM1616792 |
| Riserius sp. BOBA007 | OQ075702 | NONEP049-21 | BOLD:AEJ1230 | BON_65_2020 |
| Riserius sp. OR090 | KU197840 | ORNE387-21 | BOLD:AEL6096 | Pili_OR090_E4B4_R |
| Riserius sp. OR094 | KU197839 | ORNE399-21 | BOLD:AEL6097 | Pili_OR094_E3I4 |
| Sacconemertopsis belogurovi | KP270884 | GBMIN139138-18 | BOLD:ADM0273 |  |
| Siphonenteron bicolor | MK047688 | GBSP17188-19 | BOLD:AAX5106 |  |
| Siphonenteron nakanoi | LC625647 | GBMNF18621-22 | BOLD:AES7690 |  |
| Siphonenteron sp. | N/A | NERS082-23 | BOLD:AFI9572 | BNOM_2188 |
| Siphonenteron sp. BOBA004 |  | DISA802-19 | BOLD:ADR9817 | LACM:DISCO:6549 |
| Siphonenteron sp. SMGM017 | N/A | NEGAM026-24 | BOLD:AGD0886 | GLTZ-2472 |
| Siphonenteron sp. SMOM059 | KY561816 |  | BOLD:AFA4873 |  |
| Siphonenteron sp. SMOM059 | N/A | NEGAM027-24 | BOLD:AGD0885 | GLTZ-2473 |
| Siphonenteron sp. SMOM059 | PP834861 | NOMAN161-23 | BOLD:AFA4873 | BOMAN-10521 |
| Siphonenteron sp. SMOM059 | PP834862 | NOMAN160-23 | BOLD:AFA4874 | BOMAN-8280 |
| Sonnenemertes cantelli | MF512120 | GBSP14798-19 | BOLD:ADW2671 |  |
| Tenuilineus albocinctus | KP697776 | NORGE071-14 | BOLD:AAM2284 | NemBar1446 |
| Tetranemertes aff. rubrolineata | N/A | NERS023-23 | BOLD:AFJ0440 | BOIMB_2781 |
| Tetranemertes aff. rubrolineata | N/A | NERS065-23 | BOLD:AFJ0439 | BOIMB_2831 |
| Tetranemertes aff. rubrolineata | N/A | NERS073-23 | BOLD:AFJ0441 | BNOM_0974 |
| Tetranemertes aff. rubrolineata | OQ321714 | NOMAN046-23 | BOLD:AFA7128 | BOMAN-08053 |
| Tetranemertes antonina | KF935534 | TETRW001-21 | BOLD:AEL7530 | TE4_DR23 |
| Tetranemertes arabica | OQ321709 | NOMAN045-23 | BOLD:AFA7129 | BOMAN-08300 |
| Tetranemertes arabica | OQ321712 | NOMAN041-23 | BOLD:AFA7129 | BOMAN-08030 |
| Tetranemertes bifrost | KF935533 | GBSP11458-19 | BOLD:ADW2421 |  |
| Tetranemertes cf. | N/A | NERS094-23 | BOLD:AFJ0438 | BNOM_2533 |
| Tetranemertes majinbuui | MT578864 | CARNE495-21 | BOLD:AEL3039 | Hoplo_CB056_CB056_18_01 |
| Tetranemertes majinbuui | MW021889 | CARNE025-19 | BOLD:AEA9922 | Hoplo_CB056_1502 |
| Tetranemertes ocelata | MW021887 | CARNE022-19 | BOLD:AEA9519 | Hoplo_CB055_685_061202_1 |
| Tetranemertes pastafariensis | MT578892 | CARNE529-21 | BOLD:AEL2728 | Hoplo_CB153_CB055_18_06 |
| Tetranemertes paulayi | OQ321720 | NOMAN051-23 | BOLD:AFA4223 | BOMAN-08284 |
| Tetranemertes paulayi | OQ321720 | NOMAN051-23 | BOLD:AFA4223 | BOMAN-08284 |
| Tetranemertes sp. |  | INV764-17 | BOLD:ADY0747 | CNP-Inv 2480 |
| Tetranemertes sp. ETP001 | MT578897 | NOPP001-21 | BOLD:AEK7525 | ETP001_SMPP0632 |
| Tetranemertes sp. SMGM035 | N/A | NEGAM047-24 |  | GLTZ-0286 |
| Tetranemertes unistriata | MT578861 | TETRW013-21 | BOLD:AEL9916 | 490_070999_1 |
| Tetranemertes unistriata | OQ321719 | NOMAN037-23 | BOLD:AFA7130 | BOMAN-07032 |
| Tetranemertes unistriata | OQ321719 | NOMAN037-23 | BOLD:AFA7130 | BOMAN-07032 |
| Tetraneuronemertes lovgreni | EF208982 | GBSP5814-13 | BOLD:ACH2412 |  |
| Tetrastemma albidum | EF157598 | GBSP5817-13 | BOLD:ACH3108 |  |
| Tetrastemma albomaculatum | MZ216537 | GBMNF18520-22 | BOLD:AET4649 |  |
| Tetrastemma bilineatum | OK081695 | POIMB230-19 | BOLD:ADW8130 | BOIMB_0429 |
| Tetrastemma coronatum | AY791975 | GBSP0998-06 | BOLD:AAF1557 |  |
| Tetrastemma elegans | AJ436920 | GBSP0375-06 | BOLD:AAW6783 |  |
| Tetrastemma elegans | OQ323274 |  | BOLD:AEB4387 |  |
| Tetrastemma enteroplecta | MZ216546 | GBMNF18530-22 | BOLD:AES2535 |  |
| Tetrastemma flavidum |  | ADMAB248-23 | BOLD:AAF1600 | NHMO-DOT-829 |
| Tetrastemma freyae | MT247877 | GBMND32467-21 | BOLD:AEH8053 |  |
| Tetrastemma herthae | MW021927 |  |  |  |
| Tetrastemma longissimum | AY791981 | GBSP1004-06 | BOLD:AAW6804 |  |
| Tetrastemma melanocephalum | AY791984 | GBSP1007-06 |  |  |
| Tetrastemma merula | MZ216551 | GBMNF18535-22 | BOLD:AET6005 |  |
| Tetrastemma olgarum | MZ216552 | GBMNF18525-22 | BOLD:AES2536 |  |
| Tetrastemma olgarum |  | GBMTG4484-16 | BOLD:ADC8103 | NC_024670 |
| Tetrastemma parallelos | ON098244 | GBMNF77851-22 | BOLD:AEZ2301 |  |
| Tetrastemma peltatum | AY791990 | GBSP1013-06 | BOLD:AAE9423 |  |
| Tetrastemma peltatum | AY791992 | GBSP1015-06 | BOLD:AAE9424 |  |
| Tetrastemma phaeobasisae | OQ318514 |  | BOLD:AES7703 |  |
| Tetrastemma polyakovae | ON021857 |  |  |  |
| Tetrastemma pseudocoronatum | MN205521 | GBMNC35157-20 | BOLD:AEG9893 |  |
| Tetrastemma reticulatum | MZ216556 | GBMNF18539-22 | BOLD:AEM8150 |  |
| Tetrastemma roseocephalum | AB725598 | GBSP8048-13 | BOLD:ACI2181 |  |
| Tetrastemma roseocephalum | KC812592 | GBSP7824-13 | BOLD:ACI0818 |  |
| Tetrastemma roseocephalum | MZ216557 | GBMNF18540-22 | BOLD:AES3848 |  |
| Tetrastemma sp. | AY791985 | GBSP1008-06 | BOLD:AAD1974 |  |
| Tetrastemma sp. | AY791986 | GBSP1009-06 | BOLD:AAD1975 |  |
| Tetrastemma sp. | KF935541 | GBSP11466-19 | BOLD:ADX6709 |  |
| Tetrastemma sp. | KM042057 | GBMAA247-14 | BOLD:ACQ1726 |  |
| Tetrastemma sp. | KM042058 | GBMAA672-14 | BOLD:ACQ1729 |  |
| Tetrastemma sp. | KM042060 | GBMAA1580-15 |  |  |
| Tetrastemma sp. | KM042062 | GBMAA692-14 | BOLD:ACQ3009 |  |
| Tetrastemma sp. | KM042063 | GBMAA673-14 | BOLD:ACQ1727 |  |
| Tetrastemma sp. | KM042064 | GBMAA693-14 | BOLD:ACQ1728 |  |
| Tetrastemma sp. | KM042065 | GBMAA250-14 | BOLD:ACQ3733 |  |
| Tetrastemma sp. | KP697777 | NORGE030-14 | BOLD:ACM5113 | NemBar1160 |
| Tetrastemma sp. | MT247878 | GBMND32468-21 | BOLD:AEE3650 |  |
| Tetrastemma sp. | MT828301 | GBMNC35113-20 | BOLD:AEG7821 |  |
| Tetrastemma sp. | MW000065 | HIMF054-20 | BOLD:AEE8250 | USNM1616771 |
| Tetrastemma sp. | MZ216540 | GBMNF18523-22 | BOLD:AAI0710 |  |
| Tetrastemma sp. | MZ216542 | GBMNF18526-22 | BOLD:AAE9425 |  |
| Tetrastemma sp. | MZ216554 | GBMNF18537-22 | BOLD:ACI0405 |  |
| Tetrastemma sp. | MZ216558 | GBMNF18541-22 | BOLD:AET7391 |  |
| Tetrastemma sp. | MZ216559 | GBMNF18542-22 | BOLD:AET7392 |  |
| Tetrastemma sp. | MZ216561 |  | BOLD:AET7394 |  |
| Tetrastemma sp. | MZ216562 | GBMNF18545-22 | BOLD:AET7393 |  |
| Tetrastemma sp. | MZ216563 | GBMNF18546-22 | BOLD:AET7395 |  |
| Tetrastemma sp. | MZ580814 | DUTCH486-19 | BOLD:AAY6347 | DUT_1207 |
| Tetrastemma sp. | OK081411 | POIMB1006-19 | BOLD:AEA1234 | BOIMB_1442 |
| Tetrastemma sp. | OK414013 |  |  |  |
| Tetrastemma sp. | OQ134403 |  |  |  |
| Tetrastemma sp. |  | ADMAB087-23 | BOLD:AFJ6359 | NHMO-DOT-717 |
| Tetrastemma sp. |  | BBPS738-19 | BOLD:AEB5433 | 3865_DNA |
| Tetrastemma sp. |  | WIZCF100-23 | BOLD:AEK4423 | FHL-2018-2-G1 |
| Tetrastemma sp. IP | MZ216567 | GBMNF18550-22 | BOLD:AET5597 |  |
| Tetrastemma sp. BOBA029 | KU197636 | ORNE053-21 | BOLD:ADW8618 | Hoplo_OR020_E2F2 |
| Tetrastemma sp. CB059 | MW021894 |  | BOLD:AEB6290 |  |
| Tetrastemma sp. CB114 | N/A | CARNE639-23 | BOLD:AEB5008 | Hoplo_CB114_SMCP0373 |
| Tetrastemma sp. CB139 | OK081572 | CARNE374-21 | BOLD:AEI5850 | Hoplo_CB139_CB059_18_01 |
| Tetrastemma sp. CB193 | OK081379 | CARNE476-21 | BOLD:AEI3271 | Hoplo_CB193_SMCP0032 |
| Tetrastemma sp. CB194 | OK081331 | CARNE482-21 | BOLD:AEI9053 | Hoplo_CB194_SMCP0219 |
| Tetrastemma sp. CB195 | OK081772 | CARNE485-21 | BOLD:AEI5854 | Hoplo_CB195_SMCP0183 |
| Tetrastemma sp. CB197 | OK081719 | CARNE489-21 | BOLD:AEI5855 | Hoplo_CB197_SMCP0017 |
| Tetrastemma sp. ETP076 | N/A | NOPP143-23 | BOLD:AFR4064 | Hoplo_ETP076_SMPP0814 |
| Tetrastemma sp. ETP077 | N/A | NOPP162-23 | BOLD:AFQ7534 | Hoplo_ETP077_SMPP0847 |
| Tetrastemma sp. ETP084 | N/A | NOPP173-23 | BOLD:AFR4065 | Hoplo_ETP084_SMPP0021 |
| Tetrastemma sp. F7 | MZ216564 | GBMNF18547-22 | BOLD:AES5107 |  |
| Tetrastemma sp. GA | MZ216565 | GBMNF18548-22 |  |  |
| Tetrastemma sp. GU | MZ216566 | GBMNF18549-22 | BOLD:AET5596 |  |
| Tetrastemma sp. IR | MZ216568 | GBMNF18551-22 | BOLD:AES5108 |  |
| Tetrastemma sp. IT | MZ216569 | GBMNF18552-22 | BOLD:AES5109 |  |
| Tetrastemma sp. J | MZ216570 | GBMNF18553-22 | BOLD:AES5110 |  |
| Tetrastemma sp. M1 | MZ216573 | GBMNF18556-22 | BOLD:AES5111 |  |
| Tetrastemma sp. S | MZ216575 | GBMNF18558-22 | BOLD:AET1612 |  |
| Tetrastemma sp. SMGM004 | N/A | NEGAM006-24 | BOLD:AGD0325 | GLTZ-0254 |
| Tetrastemma sp. SMGM005 | N/A | NEGAM007-24 | BOLD:AGD0324 | GLTZ-0255 |
| Tetrastemma sp. SMGM006 | N/A | NEGAM008-24 | BOLD:AGD0323 | GLTZ-0256 |
| Tetrastemma sp. SMGM008 | N/A | NEGAM012-24 | BOLD:AGD0322 | GLTZ-0264 |
| Tetrastemma sp. SMGM013 | N/A | NEGAM015-24 | BOLD:AGD0321 | GLTZ-0278 |
| Tetrastemma sp. SMGM015 | N/A | NEGAM023-24 | BOLD:AGD0320 | GLTZ-0409 |
| Tetrastemma sp. SMGM018 | N/A | NEGAM029-24 | BOLD:AET5596 | GLTZ-2485 |
| Tetrastemma sp. SMGM020 | N/A | NEGAM033-24 | BOLD:AGD0319 | GLTZ-2504 |
| Tetrastemma sp. SMGM036 | N/A | NEGAM034-24 | BOLD:AGD0318 | GLTZ-0243 |
| Tetrastemma sp. SMGM038 | N/A | NEGAM058-24 | BOLD:AGD0317 | GLTZ-0379 |
| Tetrastemma sp. SMOM005 | PP834864 | NOMAN014-23 | BOLD:AFB4583 | BOMAN-7015 |
| Tetrastemma sp. SMOM006 | N/A | NERS063-23 | BOLD:AFI9429 | BOIMB_2829 |
| Tetrastemma sp. SMOM006 | PP834868 | NOMAN016-23 | BOLD:AFB4584 | BOMAN-8276 |
| Tetrastemma sp. SMOM007 | PP834872 | NOMAN028-23 | BOLD:AFB4585 | BOMAN-10517 |
| Tetrastemma sp. SMOM008 | N/A | NERS026-23 | BOLD:AFJ0141 | BOIMB_2805 |
| Tetrastemma sp. SMOM008 | PP834882 | NOMAN031-23 | BOLD:AFA7772 | BOMAN-8271 |
| Tetrastemma sp. SMOM008 | PP834884 | NOMAN258-23 | BOLD:AFJ0140 | BOMAN-15333 |
| Tetrastemma sp. SMOM008 | PP834885 | NOMAN032-23 | BOLD:AFA7771 | BOMAN-10508 |
| Tetrastemma sp. SMOM009 | PP834888 | NOMAN033-23 | BOLD:AFA7768 | BOMAN-7021 |
| Tetrastemma sp. SMOM012 | PP834889 | NOMAN036-23 | BOLD:AFA7767 | BOMAN-9050 |
| Tetrastemma sp. SMOM018 | PP834890 | NOMAN054-23 | BOLD:AFA7770 | BOMAN-8285 |
| Tetrastemma sp. SMOM019 | N/A | NERS095-23 | BOLD:AFA7764 | BNOM_2860 |
| Tetrastemma sp. SMOM019 | PP834897 | NOMAN064-23 | BOLD:AFA7769 | BOMAN-7022 |
| Tetrastemma sp. SMOM019 | PP834905 | NOMAN067-23 | BOLD:AFA7764 | BOMAN-10516 |
| Tetrastemma sp. SMOM020 | N/A | NERS022-23 | BOLD:AFI9509 | BOIMB_2779 |
| Tetrastemma sp. SMOM020 | PP834915 | NOMAN075-23 | BOLD:AFA7765 | BOMAN-7019 |
| Tetrastemma sp. SMOM020 | PP834916 | NOMAN076-23 | BOLD:AFA7766 | BOMAN-10484 |
| Tetrastemma sp. SMOM020 | PP834919 | NOMAN079-23 | BOLD:AFA7763 | BOMAN-10494 |
| Tetrastemma sp. SMOM033 | PP834923 | NOMAN113-23 | BOLD:AFB3967 | BOMAN-4284 |
| Tetrastemma sp. SMOM034 | PP834924 | NOMAN114-23 | BOLD:AFB3968 | BOMAN-1584 |
| Tetrastemma sp. SMOM063 | PP834925 | NOMAN166-23 | BOLD:AFB3965 | BOMAN-8061 |
| Tetrastemma sp. SMOM077 | PP834926 | NOMAN191-23 | BOLD:AFB3966 | BOMAN-11518 |
| Tetrastemma sp. SMOM083 | PP834927 | NOMAN229-23 | BOLD:AFI9508 | BOMAN-11509 |
| Tetrastemma sp. SMOM095 | PP834928 | NOMAN269-23 | BOLD:AFJ0230 | BOMAN-11556 |
| Tetrastemma sp. SMRS001 | N/A | NERS028-23 | BOLD:AFJ0231 | BOIMB_2818 |
| Tetrastemma sp. SMRS007 | N/A | NERS105-23 | BOLD:AFJ0232 | BOIMB_2820 |
| Tetrastemma sp. SMRS024 | N/A | NERS069-23 | BOLD:AFJ0234 | BNOM_0967 |
| Tetrastemma sp. SMRS029 | N/A | NERS092-23 | BOLD:AFJ0235 | BNOM_2325 |
| Tetrastemma sp. U | MZ216578 | GBMNF18561-22 | BOLD:AET1614 |  |
| Tetrastemma sp. V | MZ216579 | GBMNF18562-22 | BOLD:AET8708 |  |
| Tetrastemma sp. VE | MZ216580 | GBMNF18563-22 | BOLD:AET1613 |  |
| Tetrastemma sp. VI | MZ216581 | GBMNF18564-22 | BOLD:AET8707 |  |
| Tetrastemma sp. VT | MZ216582 | GBMNF18565-22 | BOLD:AET8709 |  |
| Tetrastemma strandae | ON021856 |  |  |  |
| Tetrastemma sundbergi | ON021855 |  |  |  |
| Tetrastemma vermiculus | AY791995 | GBSP1018-06 | BOLD:AAF1647 |  |
| Tetrastemma vermiculus | AY791997 | GBSP1020-06 | BOLD:AAF1649 |  |
| Tetrastemma vermiculus | MZ216584 | GBMNF18570-22 | BOLD:AAF1648 |  |
| Tetrastemma vermiculus |  | ADMAB085-23 | BOLD:AFJ2552 | NHMO-DOT-715 |
| Tetrastemma wilsoni | AJ436921 | GBSP0376-06 | BOLD:AAW6805 |  |
| Tetrastemma_1 sp. | MZ216549 | GBMNF18533-22 | BOLD:AAD1973 |  |
| Tetrastemma_4 sp. | MZ216548 | GBMNF18532-22 | BOLD:AAD1976 |  |
| Tetrastemma_not vittigerum | KF935540 | GBSP11432-19 | BOLD:ADW3250 |  |
| Tetrastemma_not vittigerum | MZ216585 | GBMNF18571-22 | BOLD:ADW3251 |  |
| Tetrastemmatidae sp. | KF935542 | GBSP11468-19 | BOLD:ADX0994 |  |
| Tetrastemmatidae sp. | KP270883 | GBSP11421-19 | BOLD:ADX0993 |  |
| Tetrastemmatidae sp. | MN205519 | GBMNC35159-20 | BOLD:AEG9307 |  |
| Tetrastemmatidae sp. | MN205520 | GBMNC35158-20 | BOLD:AEG9308 |  |
| Tetratemma sp. CB112 | N/A | NECOL067-23 | BOLD:AEB6654 | Co_Tet_009 |
| Tortus sp. | KP270881 | GBMIN139139-18 | BOLD:ADM1086 |  |
| Tortus sp. | ON357601 | GBMNF78009-22 | BOLD:AEZ4889 |  |
| Tortus tokmakovae | MN205527 | GBMNC35151-20 | BOLD:AEF9914 |  |
| Tubulanidae sp. | KP270865 | GBSP10432-18 | BOLD:ADM4275 |  |
| Tubulanidae sp. | KP697721 | NORGE008-14 | BOLD:ACM5270 | NemBar1130 |
| Tubulanidae sp. | KY296908 | GBSP14661-19 | BOLD:ADW5488 |  |
| Tubulanidae sp. | KY296909 | GBSP14671-19 | BOLD:ADW5487 |  |
| Tubulanidae sp. | KY296911 | GBSP14663-19 | BOLD:ADX5760 |  |
| Tubulanidae sp. | MF512114 | GBSP14778-19 | BOLD:ADW5485 |  |
| Tubulanidae sp. | MF512115 | GBSP14779-19 | BOLD:ADW5483 |  |
| Tubulanidae sp. | MF512116 | GBSP14780-19 | BOLD:ADW5484 |  |
| Tubulanidae sp. | MN205494 | GBMNC35184-20 | BOLD:AEG3069 |  |
| Tubulanidae sp. | MN205495 | GBMNC35183-20 | BOLD:AEG3068 |  |
| Tubulanus aff. lacteus | OQ075667 | NONEP030-21 | BOLD:AEJ8707 | BON_24_2020 |
| Tubulanus ambiguus | KP697780 | NORGE058-14 | BOLD:ACM5366 | NemBar1388 |
| Tubulanus annulatus | KP697781 | NORGE016-14 | BOLD:AAW8570 | NemBar1145 |
| Tubulanus cf. aureus | N/A | NERS090-23 | BOLD:AFI9431 | BNOM_2322 |
| Tubulanus cf. aureus | PP834929 | NOMAN121-23 | BOLD:AFA2649 | BOMAN-3161 |
| Tubulanus cf. lutescens | ON021854 |  |  |  |
| Tubulanus ezoensis | MZ772880 |  |  |  |
| Tubulanus ezoensis | MZ772881 |  |  |  |
| Tubulanus linearis | KP697782 | NORGE082-14 | BOLD:ACM6031 | NemBar1458 |
| Tubulanus lutescens | EU489498 |  |  |  |
| Tubulanus misakiensis | LC646454 | GBMNF73693-22 |  |  |
| Tubulanus pellucidus | HQ848625 |  |  |  |
| Tubulanus pellucidus | MW000069 | HIMF081-20 | BOLD:AEE4141 | USNM1616722 |
| Tubulanus pellucidus | OQ323324 |  |  |  |
| Tubulanus polymorphus | KP697783 | NORGE059-14 | BOLD:ACM5406 | NemBar1441 |
| Tubulanus polymorphus | KX853120 | GBSP14670-19 | BOLD:ADW9277 |  |
| Tubulanus polymorphus |  | CMBIA368-11 | BOLD:AAU4209 | MBI-SCCWRP-00368 |
| Tubulanus punctatus | KP270872 | GBSP11423-19 | BOLD:AAW8561 |  |
| Tubulanus rhabdotus | AJ436948 | GBSP0403-06 | BOLD:AAW8548 |  |
| Tubulanus riceae | MW021930 | CARNE070-19 | BOLD:AEA8543 | Palaeo_CB122_BdT035_1 |
| Tubulanus ruber | KU197697 | ORNE159-21 | BOLD:ADW2048 | Palaeo_OR052_E2F5 |
| Tubulanus ruber | KX853122 | GBSP14725-19 | BOLD:ABA9388 |  |
| Tubulanus sexlineatus |  | DISA603-19 | BOLD:ADM0945 | LACM:DISCO:3585 |
| Tubulanus sp. | KP270868 | GBSP11426-19 | BOLD:ADW6842 |  |
| Tubulanus sp. | KP270870 | GBSP10436-18 | BOLD:ADM5010 |  |
| Tubulanus sp. | KP270871 | GBSP10437-18 | BOLD:ADM5011 |  |
| Tubulanus sp. | MT811763 | GBMNE6997-21 | BOLD:AEN8119 |  |
| Tubulanus sp. | MW000067 | HIMF029-20 | BOLD:AEE4001 | USNM1616809 |
| Tubulanus sp. | MW000068 | HIMF041-20 | BOLD:AEE3496 | USNM1616788 |
| Tubulanus sp. | MZ772884 |  |  |  |
| Tubulanus sp. | MZ772885 |  |  |  |
| Tubulanus sp. | MZ772886 |  |  |  |
| Tubulanus sp. | N/A | N/A | N/A | BMOO_09145 |
| Tubulanus sp. |  | ASGRD086-18 | BOLD:ADS4890 | USNM_1512603 |
| Tubulanus sp. |  | BBPS856-19 | BOLD:AEB6539 | 3348_DNA |
| Tubulanus sp. |  | BBPS958-19 | BOLD:ADX2660 |  |
| Tubulanus sp. |  | CMBIA369-11 | BOLD:ACH0545 | MBI-SCCWRP-00369 |
| Tubulanus sp. |  | CMBIA535-12 | BOLD:ACH0595 | MBI-SCCWRP-00471 |
| Tubulanus sp. |  | HCHAR532-19 | BOLD:AFK7947 | CHARS00335-H01 |
| Tubulanus sp. CB120 | MW021928 | CARNE068-19 | BOLD:AEB0341 | Palaeo_CB120_5_2013 |
| Tubulanus sp. CB121 | OK081657 | CARNE352-21 | BOLD:AEI7165 | Palaeo_CB121_CB121_18_01 |
| Tubulanus sp. CB155 | OK081329 | CARNE413-21 | BOLD:AEI7166 | Palaeo_CB155_MCB006_18_03 |
| Tubulanus sp. CB163 | OK081637 | CARNE423-21 | BOLD:AEI7164 | Palaeo_CB163_MCB006_18_02 |
| Tubulanus sp. ETP013 | OK081450 | NOPP011-21 | BOLD:AEL5667 | ETP013_B2_A4 |
| Tubulanus sp. ETP081 | N/A | NOPP184-23 | BOLD:AFR2870 | Palaeo_ETP081_SMPP0687 |
| Tubulanus sp. ETP081 | N/A | NOPP191-23 | BOLD:AFR2869 | Palaeo_ETP081_SMPP0040 |
| Tubulanus sp. ETP082 | N/A | NOPP190-23 | BOLD:AFQ7032 | Palaeo_ETP082_SMPP0802 |
| Tubulanus sp. ETP087 | N/A | NOPP192-23 | BOLD:AFR6412 | Palaeo_ETP087_SMPP0002 |
| Tubulanus sp. OR047 | OK081674 | ORNE142-21 | BOLD:AEL0381 | Palaeo_OR047_RE830 |
| Tubulanus sp. OR048 | KU197709 | ORNE144-21 | BOLD:ADM7498 | Palaeo_OR048_E3C4 |
| Tubulanus sp. OR053 | KU197701 | ORNE160-21 | BOLD:AEL0382 | Palaeo_OR053_E1H3 |
| Tubulanus sp. OR055 | OK081469 | ORNE169-21 | BOLD:AEL0380 | Palaeo_OR055_22VIII16 |
| Tubulanus sp. SMGM032 | N/A | NEGAM085-24 | BOLD:AEE4001 | GLTZ-2499 |
| Tubulanus sp. SMOM037 | PP834930 | NOMAN119-23 | BOLD:AFA6233 | BOMAN-7052 |
| Tubulanus sp. SMOM039 | PP834932 | NOMAN122-23 | BOLD:AFB2986 | BOMAN-3183 |
| Tubulanus sp. SMOM040 | PP834933 | NOMAN123-23 | BOLD:AFB4484 | BOMAN-9051 |
| Tubulanus sp. SMOM041 | PP834934 | NOMAN124-23 | BOLD:AFA6213 | BOMAN-10528 |
| Tubulanus sp. SMOM042 | PP834935 | NOMAN125-23 | BOLD:AFB3846 | BOMAN-10527 |
| Tubulanus sp. SMOM078 | PP834936 | NOMAN192-23 | N/A | BOMAN-10500 |
| Tubulanus superbus | KP697784 | NORGE055-14 |  | NemBar1385 |
| Uniporus alisae | MF512121 | GBSP14769-19 | BOLD:ADW3114 |  |
| Valenciniidae gen. sp. | LC190961 | GBMNF18636-22 | BOLD:AET8733 |  |
| Valencinura bahusiensis | FJ811498 | GBSP2035-10 | BOLD:AAM3030 |  |
| Valencinura bahusiensis | GU392026 | GBSP4587-12 | BOLD:AAM3030 |  |
| Valencinura sp. | LC190964 | GBMNF18638-22 |  |  |
| Vieitezia luzmurubeae | KF935544 | GBSP11453-19 | BOLD:ADW7432 |  |
| Vulcanonemertes rangitotoensis | AB505828 | GBSP14164-19 | BOLD:ADW9543 |  |
| Yamaokanemertes sp. ETP092 | N/A | NOPP228-23 | BOLD:AFR8392 | Pili_ETP092_SMPP0869 |
| Yininemertes pratensis | KY274010 | GBSP14339-19 | BOLD:ADW7921 |  |
| Zygeupolia rubens | EF124982 | GBSP3710-12 | BOLD:ACB0097 |  |
| Zygeupolia rubens | HQ997773 | GBMNA12761-19 | BOLD:ADC8702 |  |
| Zygonemertes simonae | AJ436922 | GBSP0377-06 | BOLD:AAJ9798 |  |
| Zygonemertes sp. | KF935543 | GBSP11441-19 | BOLD:ADX3932 |  |
| Zygonemertes sp. | KF935546 | GBSP11442-19 | BOLD:ADW0956 |  |
| Zygonemertes sp. | MW021931 |  |  |  |
| Zygonemertes sp. | MW021932 |  |  |  |
| Zygonemertes sp. | OQ376694 |  |  |  |
| Zygonemertes sp. |  | BHAK2534-20 | BOLD:AEJ0119 | BHAK-6680 |
| Zygonemertes sp. BOBA012 | OQ075709 | NONEP006-21 | BOLD:ADL9636 | B6_2019 |
| Zygonemertes sp. BOBA013 | OQ075711 | NONEP063-21 | BOLD:ADW7912 | BON_87_2020 |
| Zygonemertes sp. BOBA014 | OQ075713 | NONEP065-21 | BOLD:AEK0256 | BON_91_2020 |
| Zygonemertes sp. BOBA015 | OQ075714 | NONEP047-21 | BOLD:AEJ0120 | BON_63_2020 |
| Zygonemertes sp. BOBA015 |  | DISA805-19 | BOLD:ADR7155 | LACM:DISCO:6565 |
| Zygonemertes sp. CB060 | OK081462 | CARNE323-21 | BOLD:AEA9095 | Hoplo_CB060_SMCP0244 |
| Zygonemertes sp. CB124 | OK081501 | CARNE353-21 | BOLD:AEI6723 | Hoplo_CB124_CB125_18_01 |
| Zygonemertes sp. CB143 | OK081769 | CARNE387-21 | BOLD:AEI6724 | Hoplo_CB143_CBdT0066 |
| Zygonemertes sp. CB164 | MK047684 | GBSP17184-19 | BOLD:AEB1924 |  |
| Zygonemertes sp. CB180 | OK081798 | CARNE461-21 | BOLD:AEI8509 | Hoplo_CB180_SMCP0399 |
| Zygonemertes sp. CB185 | OK081676 | CARNE464-21 | BOLD:AEI6725 | Hoplo_CB185_SMCP0348 |
| Zygonemertes sp. ETP002 | OK081665 | NOPP002-21 | BOLD:AEL1026 | ETP002_SMPP0751 |
| Zygonemertes sp. OR024 | KU197642 | ORNE059-21 | BOLD:ADW7913 | Hoplo_OR024_E3D5 |
| Zygonemertes sp. OR025 | KU197641 | ORNE067-21 | BOLD:AEL1027 | Hoplo_OR025_E3C7 |
| Zygonemertes sp. SMGM022 | N/A | NEGAM046-24 | BOLD:AGC9709 | GLTZ-0281 |
| Zygonemertes sp. SMOM001 | PP834937 | NOMAN001-23 | BOLD:AFB1511 | BOMAN-1259 |
| Zygonemertes sp. SMOM002 | N/A | NEGAM036-24 | BOLD:AEI8509 | GLTZ-0247 |
| Zygonemertes sp. SMOM002 | PP834941 | NOMAN007-23 | BOLD:AEI8509 | BOMAN-11540 |
| Zygonemertes sp. SMOM003 | PP834944 | NOMAN009-23 | BOLD:AFB1509 | BOMAN-10486 |
| Zygonemertes sp. SMOM004 | PP834948 | NOMAN011-23 | BOLD:AFB1510 | BOMAN-8305 |
| Zygonemertes virescens | AJ436923 | GBSP0378-06 | BOLD:AAJ9786 |  |
| Zygonemertes virescens | KX377863 | GBSP14459-19 | BOLD:ADX4365 |  |
| Zygonemertes virescens | OQ322971 |  |  |  |
